# Structural mechanisms of drebrin-mediated F-actin network modulation

**DOI:** 10.1038/s41467-026-74543-6

**Published:** 2026-06-23

**Authors:** W. Zhao, LY Chu, G. Abis, F. Oozeer, T. Mulvaney, N. Nagar, M. Topf, PR Gordon-Weeks, MR Conte, J. Atherton

**Affiliations:** 1 Randall Centre for Cell and Molecular Biophysics, King’s College London - New Hunt’s House, Guy’s Campus, London, UK; 2 Centre for Developmental Neurobiology, King’s College London - New Hunt’s House, Guy’s Campus, London, UK; 3 Centre for Structural Systems Biology (CSSB), Hamburg, Germany; 4 Department of Integrative Virology, Leibniz Institute of Virology (LIV), Hamburg, Germany; 5 Institute for Molecular Virology and Tumour virology, University Medical Center Hamburg-Eppendorf (UKE), Hamburg, Germany

**Keywords:** Cryoelectron microscopy, Cytoskeletal proteins, Molecular conformation

## Abstract

Drebrin modulates F-actin networks and links them to other intracellular components, regulating crucial processes including neuritogenesis, synaptic plasticity, virus internalisation and cancer invasion. Using single-particle cryo-EM we characterise drebrin’s interaction with F-actin through two separate conserved actin binding domains (ABD1 and ABD2), revealing structural bases for its F-actin-modulating properties. We describe a multimodal interaction where drebrin’s ABD1 can adopt two conformations and a long flexible loop connecting to ABD2 allows the two ABDs to occupy multiple relative positions along F-actin. The flexible loop connecting the two ABDs also confers some propensity to loosely bundle F-actin. Drebrin’s ABDs bind across multiple actin protomers and their subdomains and modify the longitudinal inter-protomer interface, explaining its F-actin stabilising properties. Furthermore, we show drebrin’s binding site on F-actin is shared with other critical actin-binding and regulatory proteins, explaining their competitive displacement.

## Abstract (web summary)

Drebrin regulates crucial cellular processes through its F-actin binding and regulating functions. Here, the authors report cryo-EM structures of drebrin’s actin-binding domains complexed with F-actin, giving mechanistic insight into its F-actin regulatory activities.

## Introduction

Filamentous actin (F-actin) dynamically adopts various higher-order architectures to perform defined subcellular functions. Numerous actin-binding proteins (ABPs), which often compete with one another or act cooperatively, modulate the growth and stability of individual filaments or organise multiple filaments into various bundled and branched networks.

Drebrin, a vertebrate actin-depolymerising factor homology (ADFH) protein family member, contains an N-terminal ADFH domain, followed by F-actin binding regions of predicted helical content^1^. The majority of studies find the ADFH domain does not bind F-actin^1,2^, unlike other ADFH protein family members such as cofilin and actin-binding protein 1 (ABP1)^3^. Drebrin’s two isoforms, drebrin-A and drebrin-E, differ in the inclusion or exclusion, respectively, of a 45-amino-acid insert following the F-actin binding region. While drebrin-A is expressed in adult neurons only, drebrin-E is expressed in developing neurons and multiple other cell types.

Drebrin has key functions in specific cellular subdomains. In developing neurons, drebrin-E regulates neuronal migration, process outgrowth and filopodial stability, locating predominantly to growth cone transition zone F-actin arcs and peripheral zone filopodia bases^4–12^. In both neuronal and non-neuronal cell lines, drebrin overexpression induces filopodia formation^11–14^. In mature neurons, drebrin-A is concentrated on dendritic spine F-actin, functioning in synapse formation and remodelling and learning and memory^15–22^. Postsynaptic drebrin expression abnormalities are found in epilepsy^23^, Down syndrome and Alzheimer’s disease (AD)^24–27^, with evidence from AD mouse models suggesting drebrin is a modulator of disease progression^28,29^. Interestingly, neuronal drebrin is also a modulator of opioid addiction^30^.

Drebrin structurally supports other cell-cell junctions such as gap junctions^31–33^ and adherens junctions^34^ through linking F-actin at the recipient side with key structural and signalling proteins. Drebrin recruits CXCR4 to T-cell F-actin at the immune synapse during the immune response^35^ and negatively regulates HIV entry through this pathway^36^. Drebrin’s modulation of endocytotic F-actin networks also affects rotavirus and pseudorabies virus entry^37,38^. In the developing lens, F-actin localised drebrin is required for its proper macro-organisation^39^. Drebrin reduces vascular smooth muscle cell activation, migration and proliferation through F-actin interactions, attenuating atherosclerosis^40,41^. Drebrin is expressed in myofibroblasts in fibrotic heart, lung and liver where it exerts profibrotic affects^42,43^. Finally, drebrin upregulation and F-actin interactions are implicated in the metastasis and invasion of various cancers^44–50^.

Drebrin’s functions are critically linked to F-actin interactions through several potential molecular mechanisms. Firstly, drebrin exerts direct effects on F-actin stability and architecture. For example, drebrin has been reported to bind cooperatively, directly inhibit F-actin depolymerisation and slow barbed end growth, increase filament stiffness, reduce filament curvature and remodel F-actin by increasing the cross-over repeat from 36 to 40 nm^51–57^. Drebrin may have some propensity to cross-link F-actin; however, the literature is conflicting and/or variable on this matter^12,55,56,58,59^. Secondly, drebrin competitively displaces other ABPs from F-actin, with downstream consequences. To date, drebrin has been demonstrated to compete with F-actin disassembly-promoting factor cofilin, F-actin-bundlers fascin and α-actinin, tropomyosin and myosins, suppressing their activities^55,60–64^. Finally, drebrin links the F-actin cytoskeleton to an increasing array of structural and signalling proteins^1^. For example, drebrin links F-actin to microtubules through binding EB3, encouraging microtubule entry/retention in filopodia^7,11^ and promoting prostate cancer cell invasion^65^.

Despite the centrality of drebrin’s interaction with and regulation of F-actin in driving its cellular functions, a structural understanding is lacking. Previously, only >nanometre resolution negative stain electron microscopy (EM) and cross-linking mass-spectrometry (X-MS) have been used to investigate the binding site, reporting globular drebrin densities and inferring major and minor binding sites each spanning F-actin subdomains (SDs) 1 and 2^2^.

Here, we present 2.3 Å resolution cryo-electron microscopy (cryo-EM) reconstructions of drebrin’s actin-binding region bound to F-actin, revealing two conserved chiefly extended α-helical actin-binding domains (ABDs). The two ABDs compete sterically to define drebrin stoichiometry, and each binds across two actin protomers and 3–4 F-actin SDs to stabilise the filament. We observe no large drebrin-induced conformational changes in F-actin. The first ABD adopts two conformations upon F-actin, with implications for binding stoichiometry and ABP competition. Being separated by a flexible region, the two ABDs allow some loose bundling of F-actin. Finally, these ABDs also displace various ABPs via steric competition for F-actin binding sites.

## Results

### Drebrin binds F-actin via two actin-binding domains

We first purified drebrin-E^135-355^, thought to contain the core F-actin binding region, and a longer drebrin-E^1-431^ construct that also contains the ADFH and PP domains (Supplementary Fig. 1a). While circular dichroism (CD) indicated that both constructs are chiefly of random coil and α-helical structure, drebrin-E^1-431^ had slightly increased β-sheet content, consistent with a folded ADFH domain (Supplementary Fig. 1b).

**Fig. 1:**
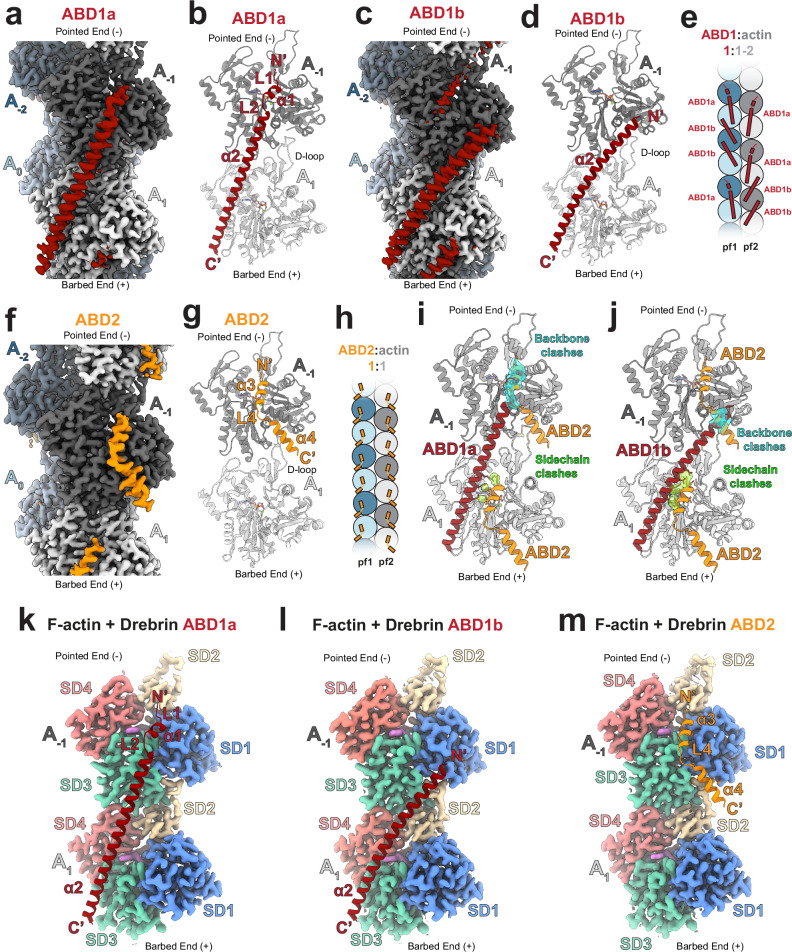
Drebrin’s ABDs each bind across two F-actin protomers and multiple subdomains, with ABD1 adopting two conformations. **a** Cryo-EM reconstruction (from our drebrin-E^135-355^ dataset) and **b** corresponding atomic model for the ABD1a conformation. **c** Cryo-EM reconstruction (from our drebrin-E^135-355^ dataset) and **d** corresponding atomic model for the ABD1b conformation. **e** Schematic model showing ABD1 assuming both ABD1a and ABD1b conformations on F-actin, such that the stoichiometry of ABD1 alone varies between 1:1-2 for F-actin. **f** Cryo-EM reconstruction (from our drebrin-E^135-355^ dataset) and **g** corresponding atomic model for ABD2. **h** Schematic model showing ABD2 alone assuming a single conformation with 1:1 stoichiometry with actin. Superimposition of the ABD2-bound F-actin model on to each protomer of the (**i**) ABD1a and (**j**) ABD1b-bound F-actin models, showing steric clashes (calculated with ChimeraX’s ‘clashes’ tool^101^ using default settings) with drebrin’s peptide backbone (cyan density) or side chains only (green density). Clashing side chains are displayed. Cryo-EM density for the drebrin ABD-bound F-actin protomer pair (A_-1_ and A_1_) is coloured according to actin subdomain (SD1-4 as labelled and Mg^2+^-ADP in magenta), with the (**k**) ABD1a, (**l**) ABD1b and (**m**) ABD2 models shown to indicate the interaction surface. In (**e**,**h**) pf1 and 2 = protofilaments 1 and 2 of F-actin. In all other panels, F-actin protomers are labelled from A_-2_ to A_1_ (from pointed end to barbed end).

Initial cryo-EM processing of F-actin with drebrin-E^135-355^ or drebrin-E^1-431^ revealed indistinguishable additional densities for drebrin-E on F-actin, which were of low resolution and only apparent at less conservative density thresholds (Supplementary Fig. 1c, d). Considering drebrin contains two reported ABDs^12^, we reasoned that if each ABD shares binding sites, their densities would likely be aberrantly averaged during image processing. To overcome this, we performed cryo-EM of F-actin with either purified drebrin-E^135-256^ or drebrin-E^256-355^ constructs that each contain only one of two reported ABDs^12^ (Supplementary Fig. 1a). Image processing including focussed classification confirmed that drebrin-E^135-256^ and drebrin-E^256-355^ each contain an ABD domain which we refer to as ABD1 and ABD2 respectively, with ABD1 adopting two alternative conformations we refer to as ABD1a and ADB1b (Supplementary Fig. 2). These three conformations (two for ABD1 and one for ABD2) were then used as references to guide a focussed supervised classification strategy to identify sites occupied with these ABD1 or ABD2 conformations on drebrin-E^135-355^ and drebrin-E^1-431^-decorated F-actin (Supplementary Figs. 3 & 4). The ABD densities derived from drebrin-E^135-355^-decorated F-actin were of the highest quality and resolution and were conformationally indistinguishable from the corresponding ABD densities in the other datasets, so were used for atomic modelling of assigned ABD sequences and structural analyses unless stated (Fig. 1a–h, Supplementary Fig. 2–6, Supplementary Table 1).

### Each drebrin ABD binds across two F-actin protomers and multiple subdomains

Both ABD1 conformations had roughly equal occupancies in all datasets (Supplementary Fig. 2–4) and partially overlapping F-actin binding surfaces (Fig. 1a–d and Supplementary Fig. 7a). In both conformations, the long helix-α2 straddles two protomers along a single F-actin protofilament (A_-1_ and A_1_) but adopt different trajectories, diverging laterally towards their N-termini (Fig. 1b, d and Supplementary Fig. 7a). ABD1a has an additional ordered N-terminal F-actin binding extension including the short helix-α1 (Fig. 1b and Supplementary Fig. 7a). ABD1 map resolution decreases and model B-factor increases towards the helix-α2 C-terminus, suggesting it has somewhat higher mobility (Supplementary Fig. 6a–c).

Due to steric restraints, a single ABD1a exclusively occupies a single protomer pair along the protofilament (Supplementary Fig. 7b). In contrast, ABD1b’s modified trajectory allows binding of another ABD1’s C-terminal end to protomer A^−1^ (Supplementary Fig. 7c, d), therefore permitting a higher potential occupancy of ABD1 on F-actin (Fig. 1e).

The 2 α-helices and a short loop of ABD2 primarily contact a single actin protomer (A_-1_), with further minor contacts with the D-loop of the next protomer along a protofilament (A_1_, Fig. 1f, g). ABD2 considered alone binds to F-actin with a stoichiometry of 1:1 (Fig. 1h). Due to steric constraints, ABD1 and ABD2 cannot occupy the same protomer pair (Fig. 1i, j).

The binding footprints of ABD1 and ABD2 also span multiple actin subdomains (SDs, Fig. 1k–m). Relative to F-actin, G-actin has a ‘twisted’ conformation where SD1 and SD2 are rotated relative to SD3 and SD4^66,67^. This ‘twisted’ conformation also exists at F-actin’s ultimate pointed end, priming it for dissociation^68,69^. Drebrin’s F-actin binding surfaces are sterically incompatible with the ‘twisted’ conformation due to these relative subdomain movements (Supplementary Fig. 8).

### Comparison of predicted and experimental structures

ABD starting templates for modelling were based on structural predictions of the drebrin-E^1-431^ interaction with AlphaFold3^70^ (Supplementary Fig. 9a). AlphaFold3 predicted the two experimentally observed predominantly α-helical ABDs with relatively high confidence; however, it only modelled the ABD1a conformation. Alphafold3 also erroneously predicted ABD1a’s helix-α2 as 26 amino acids longer C-terminally, such that ABD1a interfaced with three rather than the observed two actin protomers. Interestingly, secondary structure prediction by JPred4^71^ and AlphaFold3 in the absence of F-actin also predicted a longer helix-α2/shorter L3 than observed (Supplementary Fig. 9b, c). Consistent with this region being all a disordered L3, it has low vertebrate sequence conservation, AlphaMissense^66^ predicted substitution pathogenicity scores and AlphaFold3 PAE scores (Supplementary Fig. 9a, b). Instead, the experimentally observed length of helix-α2 and L3 by cryo-EM corresponded well with that observed previously for a drebrin residues 173–238 fragment (inclusive of helix-α2 and the N-terminal portion L3, but not helix-α1, BMRB ID 5272911) by solution-state nuclear magnetic resonance (NMR)^72^. It also agreed well with that observed here for the longer drebrin-E^135-355 15N^ (without F-actin) by solution-state heteronuclear single quantum correlation (^1^H-^15^N HSQC) NMR coupled with TALOS-N v4.21^73^ analysis to predict residue order-disorder probability (Supplementary Fig. 10). This indicates that: 1) F-actin binding does not induce major structural rearrangements to the α-helical organisation in residues 173–317 of drebrin-E^135-355^; and 2) the portions of drebrin’s L3 not resolved in our cryo-EM reconstructions likely remain intrinsically disordered in both apo and F-actin-bound states.

While AlphaFold3 also modelled the ADFH domain and regions downstream of ABD2 in proximity to F-actin, poor PAE and/or pLDDT values gave these interactions very low confidence (Supplementary Fig. 9a), consistent with our cryo-EM results indicating no additional interactions outside of the ABDs.

### F-actin-interacting residues in drebrin ABDs are highly conserved and important for drebrin function

**Fig. 2:**
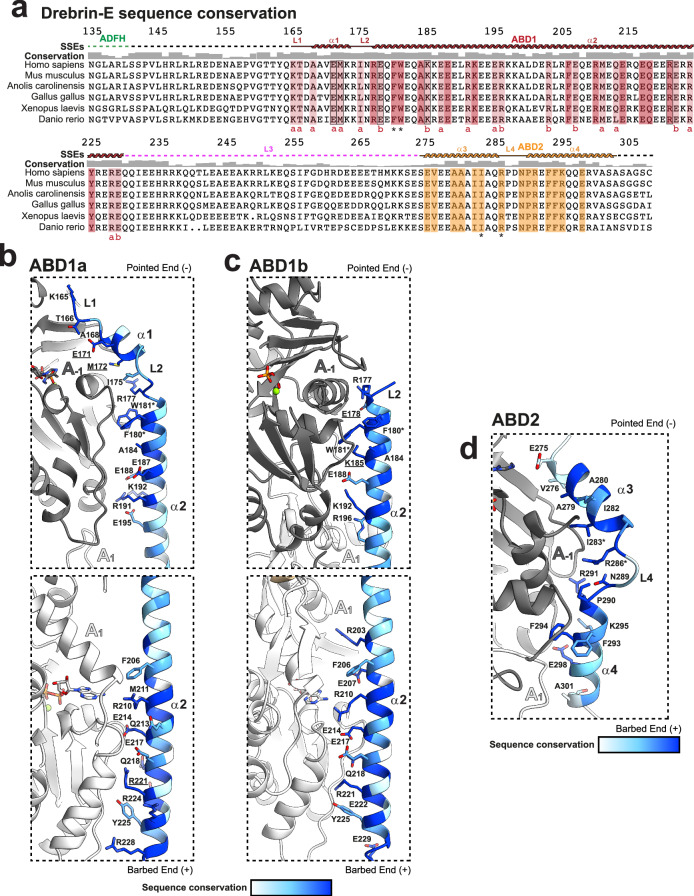
F-actin-interacting residues in Drebrin ABDs are highly conserved and important for drebrin function. **a** A multiple sequence alignment of human drebrin-E residues 135–308 with canonical drebrin orthologue sequences from 113 other vertebrate species (acquired from the Ensembl database^111^) was performed with the MUSCLE algorithm^112^. The *Homo sapiens* sequence alongside a reduced set of five vertebrate class model species (mammals, reptiles, birds, amphibians and fish) is shown, with *Homo sapiens* sequence numbering, secondary structure elements (SSEs) identified by cryo-EM and sequence conservation from the larger 113 species alignment shown above. F-actin-contacting residues are highlighted in red and orange for ABD1 and ABD2, respectively. Those contacting F-actin in both ABD1 conformations are highlighted in dark red; those in one or the other conformation are highlighted in light red, with the conformation indicated below (a = ABD1a, b = ABD1b). Residues within black boxes were mutated for the confocal analysis presented in Supplementary Fig. 12, while those with asterisks below were included in ABD double mutations presented in Supplementary Fig. 11. Our cryo-EM-derived drebrin-E-bound F-actin models are shown for (**b**) ABD1a, (**c**) ABD1b and (**d**) ABD2, with drebrin-E coloured by conservation (114 species alignment) and key F-actin interacting side chains shown/labelled. ABD1 models are broken into two images to accommodate the best views. In (**b–d**), underlined residues were mutated for the confocal analysis presented in Supplementary Fig. 12, while those with asterisks were included in ABD double mutations presented in Supplementary Fig. 11.

High vertebrate sequence conservation, particularly in F-actin-interacting residues, was found in both ABDs, including in ABD1 regions and residues that exclusively interact with F-actin in only one of the two conformations (Fig. 2a–d). Double non-conservative mutations of two neighbouring absolutely conserved hydrophobic residues interacting with F-actin in both ABD1 conformations drastically reduced drebrin-E^135-256^ association by in vitro co-sedimentation (Supplementary Fig. 11a). Similarly, double non-conservative mutations of two proximal F-actin-interacting residues in ABD2 drastically reduced drebrin-E^256-355^ association (Supplementary Fig. 11b).

Transfection of an eYFP-tagged drebrin-E^135-256^ construct (containing ABD1 alone) into COS7 cells induces filopodia when they are usually rare^11,12^. We tested the functional importance of each ABD1 conformation for filopodial induction by transfecting drebrin-E^135-256^eYFP point mutants. Substitutions were targeted to conserved charged residues that clearly either interact with F-actin in ABD1a (E171A and M172S) or ABD1b (K185A and E178A) alone or in both conformations (R221A) (Supplementary Fig. 12a–e). While K185A and M172S mutations had no effect, E171A, E178A and R221A (strongest effect) significantly suppressed filopodial induction compared to wild-type (Supplementary Fig. 12f–m). We also assessed how drebrin-E^135-256^-eYFP mutations affect cellular co-localisation with F-actin, finding M172S and R221A (strongest effect) mutations cause significant reductions (Supplementary Fig. 12n).

### Drebrin modifies the F-actin inter-protomer interface

Actin’s D-loop and C-terminus can adopt various conformations at the inter-protomer interface, associated with F-actin nucleotide state or small molecules such as phalloidin^67,74–76^. In our reconstructions, despite both ABD1 and ABD2 clearly associating with Mg^2^+-ADP-bound F-actin, we observe a closed D-loop and compact C-terminus distinct from Mg^2+^-ADP-bound F-actin alone (PDB: 8a2t^67^, Supplementary Fig. 13a–f, Supplementary Fig. 14a–c). This conformation is instead indistinguishable from that observed in the Mg^2+^-ADP-Pi-bound state of F-actin, including the inter-protomer interaction between Q41 and F375 (PDB: 8a2s^67^, Supplementary Fig. 14d–f). ABD1b and ABD2, but not ABD1a, directly interact with F-actin’s D-loop, coincident with somewhat stronger D-loop densities and lower atomic b-factors, suggesting partial stabilisation (Supplementary Fig. 13d–f).

### Drebrin does not significantly modify F-actin helical rise and twist

We next analysed our cryo-EM data for previously reported drebrin-induced F-actin twist and cross-over repeat length modification^51,52^. We first compared 9-protomer F-actin models fitted into drebrin-E^135-256^, drebrin-E^256-355^, drebrin-E^135-355^ and drebrin-E^1-431^-decorated Mg^2+^-ADP F-actin reconstructions (before focused ABD classification) with Mg^2+^-ADP F-actin alone (PDB: 8a2t^67^). When the central protomers were superimposed, we found negligible structural deviation along the F-actin axis in either direction (Supplementary Fig. 15a–d, corresponding to a cross-over repeat length change of under 3.25 Å).

We also calculated helical parameters (rise and twist) directly from these cryo-EM reconstructions in CryoSPARC v4.6.2^77^, and again the differences between these datasets and that previously reported for Mg^2+^-ADP F-actin alone were negligible (Supplementary Fig. 15e–h, Mg^2+^-ADP F-actin^67^ = 27.50 Å and 166.7°).

### Overlapping ABD1 and ABD2 binding sites and loop 3 length determine multimodal binding patterns along F-actin

**Fig. 3:**
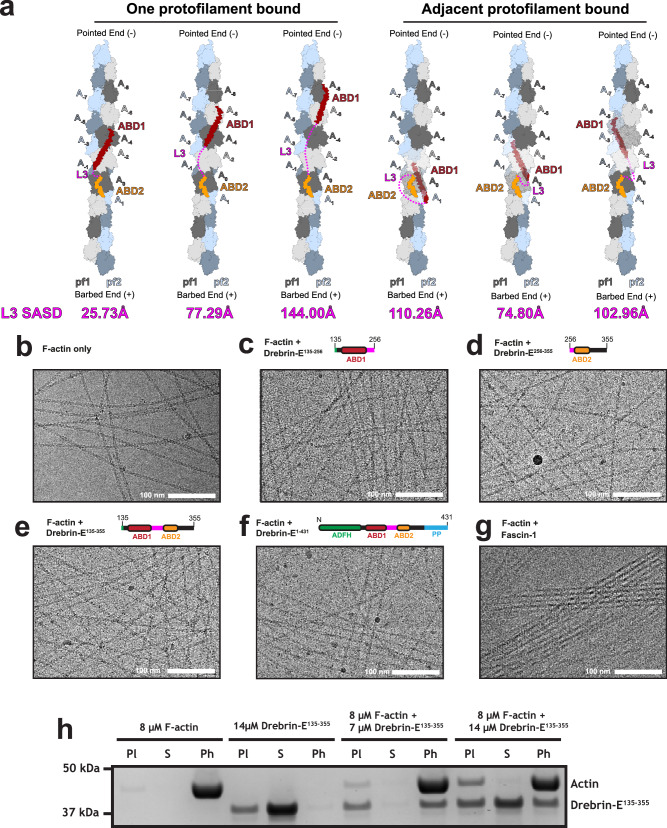
Drebrin’s ABD1 and 2 can adopt a limited number of relative positions along F-actin, or can act to loosely bundle F-actin. **a** Schematic models indicating the 6 permitted positions of ABD1 relative to ABD2 on a single actin filament. Solvent Accessible Surface Distances (SASDs)^116^ determined by JWalk^78^ between the ABD1 C-terminus and ABD2 N-terminus (i.e L3) are shown below each model. L3 length (SASD) cannot exceed a ~ 150 Å physical length limit determined by Cα-Cα distance restraints (~3.5 Å × 43 amino acids) assuming adoption of an extended linear peptide. Micrographs and domain schematics of (**b**) F-actin alone or F-actin decorated with saturating concentrations of (**c**) Drebrin- E^135-256^, (**d**) Drebrin- E^256-355^, (**e**) Drebrin- E^135-355^, (**f**) Drebrin- E^1-431^ or (**g**) bundling protein fascin-1. Representative micrographs are shown from datasets of 100 micrographs. **h** Low-speed co-sedimentation assay with F-actin and Drebrin-E^135-355^, where any bundled F-actin sediments in the low-speed pellet (Pl), unbundled F-actin sediments in the high-speed pellet (Ph) and soluble protein remains in the supernatant (S). This assay was independently repeated three times, with a single representative experiment shown.

We next investigated how ABD1 and ABD2 may be positioned relative to each other on F-actin, considering they are connected by the 43 amino acids-long flexible L3 and are not sterically permitted to bind the same protomers (Fig. 1i, j). In the following analysis we considered ABD1a and ABD1b conformations as one, as L3 emanates from roughly the same location (<2 Å difference) relative to F-actin (Supplementary Fig. 7a). Using Jwalk software^78^ to consider L3’s maximum stretch through solvent across the complex surface, six sterically permitted positions of ABD1 relative to ABD2 were determined, an equal number of which included simultaneous binding to the same or adjacent protofilaments (Fig. 3a). Interestingly, in all six conformations ABD1 always occupies a protomer pair closer to the F-actin pointed end.

Plotting the spatial distribution of 3D classes on micrographs of our drebrin-E^135-355^-decorated F-actin dataset revealed no obvious spatial relationships between or preferences for particular ABDs/conformations on individual filaments (Supplementary Fig. 16a). We next investigated whether a preferred spatial relationship of ABDs exists on neighbouring protomers along a protofilament. ABD occupancy on the adjacent protomer pair to an ABD2 (towards the pointed end) was examined with supervised classification, revealing no clear preference (Supplementary Fig. 16b).

ABD1b’s shifted conformation allows the C-terminus of another ABD1 to bind proximally (~6 Å closest distance, Supplementary Fig.7b, c), so we hypothesised that attractive inter-molecular electrostatics or indirect water-mediated hydrogen bonds between two ABD1s here could represent a cooperativity mechanism. To investigate, we performed a similar supervised classification analysis on the adjacent protomer pair to an ABD1b; however, no large preference for ABD1 at this site was found (Supplementary Fig. 16c). With 61% of ABD1b adjacent sites empty or occupied by ABD2, this analysis also suggests ABD1a is not only adopted to avoid steric clashes with an adjacent ABD1. In summary, drebrin’s two F-actin binding domains can assume up to six relative positions on a single F-actin, with, according to our analysis, no strong preference.

### Drebrin has some propensity to loosely cross-link actin filaments

We hypothesised that drebrin’s ABDs, separated by the long flexible L3, could act to bundle F-actin directly. The extent of bundling in micrographs of F-actin incubated with drebrin constructs containing either a single or both ABDs was visually indistinguishable from F-actin alone (Fig. 3b–f) and in stark contrast to F-actin incubated with tight bundling protein^79,80^ fascin-1 (Fig. 3g). However, while the distribution and relative orientations of filaments do not suggest drebrin tightly cross-links F-actin into organised colinear bundles (like fascin-1), it does not preclude looser cross-linking by a proportion of drebrin molecules. Therefore, we performed low-speed co-sedimentation assays sensitive to such looser cross-linking, which indicated that a proportion of F-actin is indeed bundled by drebrin-E^135-355^ (Fig. 3h, Supplementary Fig. 17, fascin-1 used as a positive control).

Drebrin’s tendency to induce filopodia when overexpressed in cell lines could hypothetically be derived from a bundling propensity. To test this hypothesis, we transfected COS7 cells (which normally lack filopodia) with drebrin-E-eYFP to induce filopodia as previously described^11^, then collected cryo-tomograms of these filopodia targeted by correlative light and electron microscopy (CLEM), to characterise their underlying F-actin networks (Supplementary Fig. 18a). F-actin in drebrin-E-induced filopodia was mainly organised into closely packed, collinear bundles, alongside some unbundled or more loosely bundled filaments, particularly in the lateral filopodial periphery (Supplementary Fig. 18b, c), similar to previous cryo-tomographic observations of filopodia^76,77^. Interfilament distances in bundles in filopodia were, on average, similar to those measured in fascin-linked bundles in vitro (Supplementary Fig. 18d). Considering drebrin’s ABDs are separated by a flexible L3 with a maximum stretched length of ~15 nm, it seems unlikely drebrin is the primary F-actin cross-linker organising these tight bundles, but may be responsible for loose bundles or become recruited to pre-existing tight bundles. Interestingly, in half the drebrin-E-eYFP-transfected filopodia imaged, we observed two or more microtubules (Supplementary Fig. 18c, e), often associated with small organelles, vesicles and ribosomes, which are normally rare features in wild-type filopodia^11,81–83^. This is consistent with previous light microscopy observations showing more common microtubule entry into drebrin-transfected filopodia^11,12^.

### Mechanisms of drebrin-mediated ABP displacement from F-actin

**Fig. 4:**
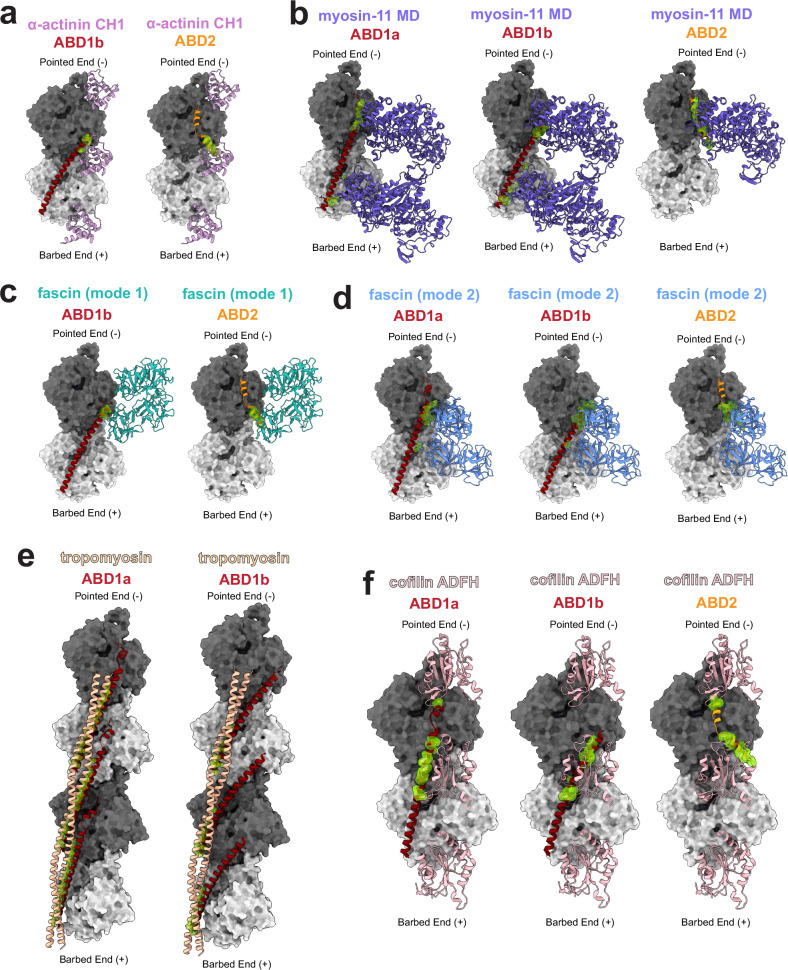
Drebrin sterically competes with key ABPs for F-actin association. Models of F-actin-bound ABPs are superimposed onto our drebrin ABD-bound F-actin models (actin portion as the reference), and resultant clashes (detected with default settings in ChimeraX^101^) are shown as green density. Superimposed ABPs; (**a**) α-actinin CH1 domain (PDB: 3LUE^117^), (**b**) myosin-11 motor domain (MD, PDB: 6BIH^118^), (**c**) fascin binding mode 1 (PDB: 8VO5^119^), (**d**) fascin binding mode 2 (PDB: 8VO6^119^), (**e**) tropomyosin (Tpm1.1, apo-state, PDB: 5JLF^120^) and (**f**) cofilin ADFH domain (PDB: 6UBY^87^). The cofilin model used was the structure of a single cofilin ADFH domain bound to F-actin, representative of an initial association event. The cofilin-bound unmodified ‘straight’ actin protomer in this model was used for superimpositions.

To describe how drebrin displaces ABPs from F-actin^55,60–63^, we superimposed (via F-actin) structures of F-actin associated with the F-actin binding domains of α-actinin, fascin, tropomyosin, myosin and cofilin onto our F-actin-bound drebrin ABD1 and ABD2 models (Fig. 4 and Supplementary Fig. 19). Both ABD1 and ABD2 sterically clashed with these ABPs in all cases apart from tropomyosin, where only ABD1 clashed. Furthermore, for all ABPs apart from α-actinin, both conformations of drebrin’s ABD1 clashed. While our structural data shows drebrin sterically blocks initial access of cofilin to its F-actin binding site, it may additionally inhibit cofilin F-actin interactions through allosteric mechanisms. First, we demonstrate that drebrin’s ABDs stabilise a conformation of F-actin’s D-loop (Supplementary Fig. 14), which may inhibit cofilin binding^74^. Second, cofilin modifies F-actin, inducing a new helical twist via rotation of actin SD3/4 relative to SD1^84–86^ and tight cofilin binding depends on this modification^87^. Therefore, by binding over multiple F-actin subdomains and protomers in unmodified actin (Fig. 1), drebrin’s ABDs may also act to inhibit this conformational change.

## Discussion

In this study, we structurally characterise how drebrin interacts with F-actin via two ABDs. A re-interpretation of previous affinity-binding data of drebrin constructs^2^ suggests that two ABDs confer a 100–1000-fold increase in affinity for F-actin than one (ABD2) alone. Previous stoichiometry measurements based on F-actin co-sedimentation assays have reported stoichiometries of 1:5 for full-length drebrin-E or -A, or ~1:3 for a drebrin-A amino acids 1–300 construct containing both ABDs (except the last three poorly conserved residues of ABD2, Fig. 2a, d)^2,51,55,61^. With two ABDs competing for binding surfaces, our data are consistent with drebrin-E^135-355^ and drebrin-E^1-431^ binding to F-actin protomers with a stoichiometry close to 1:3. Caution should be applied to the previous quantitative interpretation of stoichiometry from co-sedimentation assays, due to challenges in densiometric analyses. Nonetheless, this may indicate drebrin has additional unpredicted F-actin binding sites outside the ABDs, or additional regions that interfere with ABD-F-actin association, which have not been resolved here. Our structures present no evidence that our tested drebrin-E constructs significantly alter F-actin helical parameters, contrasting with previous atomic force microscopy (AFM) measurements using full-length drebrin-A or a truncation to residues 1–300^51,52^.

**Fig. 5:**
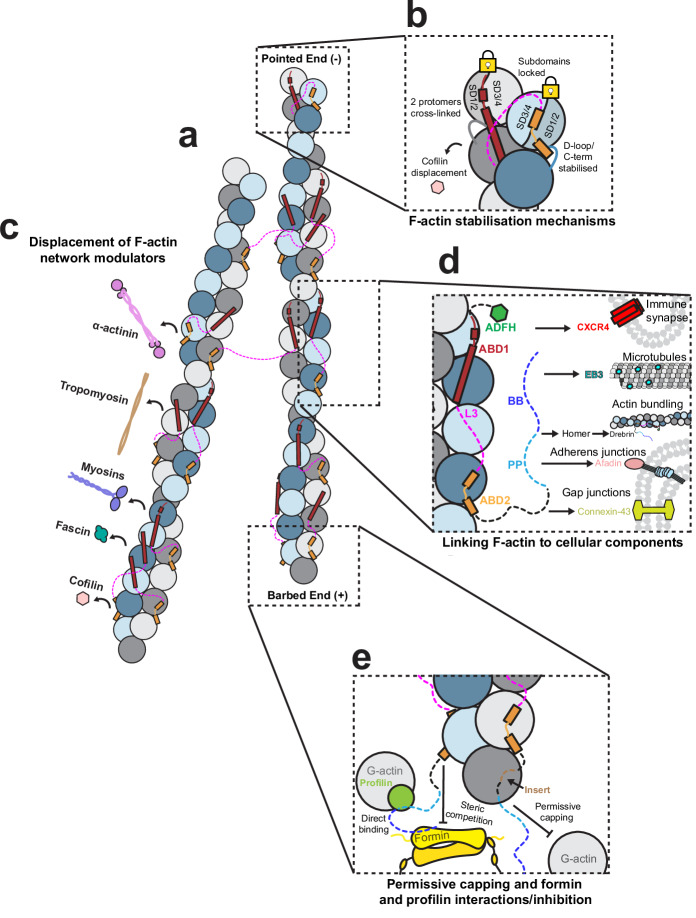
Models of multimodal drebrin-F-actin interactions, F-actin stabilisation and ABP displacement. **a** Drebrin ABD1 and ABD2, separated by a long flexible L3, can assume multiple relative positions on single or adjacent protofilaments on a single actin filament, but can also loosely cross-link two actin filaments. **b** Drebrin ABD binding across two actin protomers and their subdomain pairs SD1 & SD2 and SD3 & SD4 cross-links F-actin and stabilises the ‘flattened’ conformation in the ultimate pointed end protomers, respectively, preventing depolymerisation. **c** ABD association sterically displaces a number of other important ABPs, including cofilin, an F-actin disassembly-promoting factor, further contributing to F-actin stabilisation. **d** By associating with F-actin via a minor portion of the sequence, drebrin’s ADFH domain, L3, PP domain and BB domain are free to link F-actin to other cellular and molecular components. **e** At the F-actin barbed end, drebrin may bind to penultimate or ultimate actin protomers due to their ‘flattened’ conformation. Here, ABDs may sterically compete with formins to inhibit actin promotion of assembly. Mobile regions C-terminal to ABD2 are projected away from the barbed end where they can bind formins and profilin, but also act as a leaky barbed end cap to inhibit protomer addition. Furthermore, the drebrin-A isoform’s insertion in this region may bind the exposed face of the barbed end where protomers are added to inhibit their addition.

Because both ABDs bind axially across two protomers and actin subdomain pairs SD1/2 and SD3/4, which are rotated relative to each other in the ‘twisted’ G-actin conformation^66,67^, drebrin’s ABDs are structurally incompatible with G-actin association. This ‘twisted’ conformation is also observed at pointed (but not barbed) end F-actin protomers, favouring their dissociation^68,69^. Therefore, drebrin may stiffen F-actin and prevent its depolymerisation^51–53^ by both cross-linking protomers together and stabilising ‘flattened’ F-actin at pointed ends (Fig. 5). The observed ABD structures contrast with the previous >nanometre resolution negative stain EM and XL-MS study, which reported globular drebrin densities and major and minor binding sites at different locations on F-actin’s SD1 and SD2^2^.

Although not clarified by this study, drebrin’s ABDs would hypothetically interact with the ‘flattened’ conformations of F-actin’s barbed end ultimate and/or penultimate protomers. Drebrin has a ‘leaky’ F-actin barbed-end capping property that is dependent on flexible regions downstream of ABD2 and stronger for drebrin-A due to this isoform’s insert^56,57^. Our structures suggest these mobile regions, including drebrin-A’s insert, would be directed out from ABD2 bound at the barbed end where they could function in permissive capping, barbed end interactions and profilin/formin interactions (Fig. 5). Drebrin also inhibits barbed end F-actin assembly by some formins and directly interacts with the formin mDia2 and profilin^56,88,89^. Our structures tentatively suggest drebrin’s inhibition of formin-driven F-actin assembly could be achieved by steric competition for barbed-end formin association in addition to presentation of formin-interacting motifs (Fig. 5). Future studies should aim to further explore the structural bases of drebrin’s modulatory properties at both F-actin ends.

Another way drebrin can modify F-actin dynamics and network architectures in cells is through competing for occupancy with other ABPs. For example, competition of drebrin with cofilin^64^ may augment drebrin’s F-actin direct stabilising properties discussed above in a cellular context. Our structures demonstrate that drebrin’s ability to displace various ABPs from F-actin^55,60–63^ occurs chiefly via steric competition. For all ABPs apart from tropomyosin, both drebrin ABDs are structurally competitive, and a single ABD will often obscure ABP binding sites on multiple protomers (myosin, tropomyosin and cofilin) or multiple ABP binding modes (e.g fascin), increasing drebrin’s competitive potency. However, within an ABD-associated protomer pair, some competing ABPs remain sterically permitted to bind one of the two protomers. Therefore, this could allow drebrin to accommodate the binding of specific ABPs at sterically permitted sites and even coordinate with other ABPs to form higher-order F-actin-based macromolecular architectures, somewhat analogous to the ‘scaffolding’ function of tropomyosin in the sarcomeric thin filament^90,91^. Interestingly, like drebrin, tropomyosin is another ABP where long α-helical secondary structure can take multiple paths across F-actin, a feature allowing it to adaptively incorporate other ABPs in multimeric complexes^92^.

The two ABD1 conformations we observed permit different drebrin-E stoichiometries on F-actin and, due to their different F-actin interfaces, have different propensities to sterically compete with various ABPs. Mapping sequence conservation onto our models suggests both conformations may play conserved physiological roles, yet their relative prevalence and functional importance in particular contexts are unclear. Our analyses of the effects of ABD1 mutations on filopodial induction and F-actin co-localisation suggest both conformations could be functionally important, with a mutation targeting both conformations having the strongest effects.

We demonstrate that drebrin’s two ABDs are separated by L3, a loop of 43 amino acids which is disordered both in solution and when drebrin is bound to F-actin. Inter-ABD competition and flexible L3 length allow the two ABDs of a drebrin molecule to associate with F-actin by adopting six relative positions along the same or adjacent protofilaments. Furthermore, we present biochemical evidence that drebrin-E^135-355^ can loosely bundle F-actin without any discernible effect on the relative orientations of cross-linked filaments by cryo-EM. This finding is consistent with our structural data and explains why, whilst some studies find biochemical evidence of drebrin bundling^12,59^, changes in F-actin distribution have generally not been observed by EM or AFM^2,51,55,58^. Drebrin’s propensity to cross-link filaments rather than bind in a tandem ABD arrangement along a single filament likely depends on local actin filament concentrations and organisation. For example, drebrin cross-linking may be particularly prevalent at sites of pre-existing F-actin bundles, to which it is generally localised in cells. While drebrin could promote filopodia formation through such cross-linking, constructs containing a single ABD of drebrin induce filopodia comparably to constructs containing both ABDs^12^. Filopodia induction may instead result from F-actin stabilisation, competition with other F-actin-organising ABPs at filopodial bases/initiation sites and/or increased entry and/or retention of microtubules in filopodia.

Interestingly, high sequence conservation and AlphaMissense^93^ pathogenicity scores are observed in the N-terminal portion of L3 despite our cryo-EM maps and solution-state NMR showing it as disordered. Recent solution-state NMR studies of drebrin constructs containing either ABD1 or ABD2, including the N-terminal portion of L3, also reported this region as disordered^72,94^. This suggests L3’s N-terminus could serve as a protein-binding or post-translational modification site and/or be involved in modifying the F-actin association of ABD1 or its position relative to ABD2. Interestingly, neurofibromin-2 was recently reported to bind to this conserved region of L3, facilitating liver tumorigenesis via the Hippo signalling pathway^46^. Drebrin (or its F-actin binding region alone) binds F-actin cooperatively, accumulating on F-actin in concentrated clusters^51,52,54^.

An inter-molecular cooperativity mechanism could not be determined from our ABD-F-actin structures; however, flexible regions of drebrin not observed in our density maps could interact to mediate cooperativity. Alternatively, while our tested drebrin-E constructs did not affect F-actin twist or rise, cooperativity could be mediated through more subtle modifications to F-actin structure. Interestingly, despite the presence of Mg^2+^-ADP in the nucleotide pocket, F-actin’s D-loop and C-terminus were shown to adopt conformations associated with more stable ‘young’ F-actin prior to P_i_ release^67,74^. These conformations were seen at longitudinal protomer interfaces, both where drebrin-E^135-355^ does or does not contact the D-loop (with higher atomic B-factors in non-contacting sites), suggesting drebrin’s effect on these elements could be transmitted by long-range allosteric communication. Given that drebrin ABDs interact directly with this loop, transmission of D-loop conformation could contribute to a cooperativity mechanism.

Future work should seek to understand how drebrin’s structure and interaction with F-actin and other binding partners are regulated. For example, phosphorylation of S142, located between the ADFH domain and ABD1, regulates the full-length drebrin fold to modulate ABD1 availability and F-actin cross-linking, as well as exposure of the EB3 binding site^12^. In addition to S142, several other phosphosites, including S274, are responsive to chemically induced long-term depression^95^. Although not an F-actin-interacting residue, S274 is located at ABD2’s N-terminus (Fig. 2) such that its phosphorylation could modify ABD2 structure or F-actin binding. Interestingly, binding of drebrin to BTP2, an inhibitor which reduces the F-actin interaction, is eliminated by the mutation of two proximal lysines just upstream of ABD2; K270 and K271^96^.

In summary, here we have structurally detailed the multimodal interaction of drebrin’s ABDs with F-actin and identified mechanisms of drebrin-mediated F-actin-stabilisation and ABP displacement (Fig. 5). Furthermore, by associating with F-actin via a minor portion of the sequence, drebrin can also serve as platform linking F-actin to structural and signalling proteins, or act in barbed end permissive capping, particularly through its remaining flexible or flexibly tethered regions (Fig. 5). The variability in binding modes may add a complexity to drebrin’s regulation of F-actin architecture and the association of other ABPs and off-actin binding partners, a subject that warrants future investigation.

## Methods

### Plasmids and site-directed mutagenesis

His-tagged drebrin-E^256-355^ and drebrin-E^135-355^ constructs for protein expression and purification in *Escherichia coli* were previously described and correspond to regions previously characterised as the ‘Hel’ domain or ‘CC and Hel’ domains, respectively^12^. Briefly, for drebrin-E^256-355^, human drebrin-E residues 256–355 were subcloned into the pET-24a(+) vector (Novagen). For drebrin-E^135-355^, human drebrin-E residues 135-355 were subcloned into the pET-30a(+) vector (Novagen). For drebrin-E^1-431^ (inclusive of the ADFH domain, regions previously referred to as the ‘CC’ and ‘Hel’ domains and the PP domain^12^), human drebrin-E residues 1-431 were subcloned into the pET-24a(+) vector (Novagen). To make drebrin-E^135-256^ (corresponding to a region previously referred to as the ‘CC’ domain^12^), a stop codon was added to drebrin-E^135-355^ after position 256 by site-directed mutagenesis using the manufacturer’s instructions alongside manufacturer-provided primer designer software (Q5® Site-Directed Mutagenesis Kit, NEB). To make drebrin-E^135-256 F180S W181S^ and drebrin-E^256-355 I283S R286A^ double mutants, the Q5® Site-Directed Mutagenesis Kit (NEB) was used with drebrin-E^135-256^ or drebrin-E^256-355^ constructs as backbones, respectively.

The drebrin-E-eYFP (full-length) construct expressed in COS7 cells for CLEM and cryo-electron tomography (cryo-ET) analysis has been previously described^11^. The drebrin-E^135-256^-eYFP construct used for filopodial induction assays in COS7 cells and as a backbone for site-directed mutagenesis has been previously described^12^. Briefly, for drebrin-E-eYFP and drebrin-E^135-256^-eYFP, human full-length drebrin-E or residues 135–256, respectively, were subcloned into the pEYFP-N1 vector (Clontech). Site-directed mutagenesis to produce drebrin-E^135-256^-eYFP mutants for expression in COS7 cells was performed using the drebrin-E^135-256^-eYFP construct as a backbone and the Q5® Site-Directed Mutagenesis Kit (NEB), using the manufacturer’s instructions alongside manufacturer-provided primer designer software.

### Protein expression and purification

All drebrin constructs for cryo-EM were expressed in *Escherichia coli* Rosetta (DE3) in ZYM-5052 auto-induction medium^97^. Fascin-1 was expressed in *Escherichia coli* BL21 (DE3) in ZYM-5052 auto-induction medium. Cultures were grown at 37 °C with shaking at 180 rpm to an initial optical density 600 nm (OD₆₀₀) of 0.1, incubated for a further 4 h, then the temperature was decreased to 18 °C for overnight protein expression.

Drebrin-E^135-355^ for NMR studies was ^15^N-labelled by culturing *Escherichia coli* Rosetta (DE3) in M9 minimal media (50 mM Na_2_HPO_4_, 25 mM KH_2_PO_4_, 10 mM NaCl, 20 mM ^15^NH_4_Cl, 20 mM D-glucose, 0.3 mM CaCl_2_, 1 mM MgSO_4_, 1 mg thiamine hydrochloride, 1 mg biotin). Cells were grown at 37 °C to an optical density 600 nm (OD₆₀₀) of 0.9 then induced with 1 mM IPTG with shaking at 18 °C overnight for protein expression.

Cell pellets were lysed by sonication with buffer containing 20 mM HEPES, pH 7.5, 500 mM NaCl, 10% (w/v) glycerol, and EDTA-free protease inhibitor. For all constructs, lysates were clarified by centrifugation at 48,400 × g for 30 min at 4 °C and loaded onto a HiTrap TALON® crude column (Cytiva) for purification by immobilised metal-ion affinity chromatography (IMAC). The column was washed with IMAC wash buffer (20 mM HEPES, pH 7.5, 500 mM NaCl). Bound protein was eluted with IMAC elution buffer (20 mM HEPES, pH 7.5, 500 mM NaCl, 250 mM imidazole), then buffer-exchanged into ion-exchange (IEX) binding buffer (20 mM HEPES, pH 7.5, 50 mM NaCl, 2 mM DTT) using a HiPrep 26/10 Desalting column (Cytiva). Protein in IEX binding buffer was then loaded onto a HiTrap Q HP anion exchange chromatography column (Cytiva), then eluted in a continuous gradient from IEX binding buffer to IEX elution buffer (20 mM HEPES, pH 7.5, 1 M NaCl, 2 mM DTT). Finally, protein was further purified by size-exclusion chromatography using a Superdex 200 Increase 10/300 GL (Cytiva) in HEPES-KME buffer (10 mM HEPES, pH 7.5, 50 mM KCl, 1 mM MgCl_2_, 1 mM EGTA, 1 mM DTT).

### F-actin co-sedimentation assays

ABPs were clarified by centrifugation at 12,000 × g for 20 min at 4 °C. For low-speed co-sedimentation assays to assess F-actin bundling, polymerised F-actin was mixed with fascin-1 or drebrin-E^135-355^ in HEPES-KME buffer, incubated on ice for 30 min, and centrifuged at 12,000 × g for 20 min at 4 °C to sediment bundled F-actin. Supernatants were then centrifuged at 436,000 × g for 15 min at 4 °C in an S100AT3 rotor (Eppendorf) to sediment the remaining filaments. Pellets from both steps were resuspended in HEPES-KME buffer and analysed together with the corresponding supernatants by SDS–PAGE.

For F-actin binding co-sedimentation assays, wild-type and mutant drebrin-E constructs were mixed with F-actin on ice for 30 min in HEPES-KME buffer and centrifuged at 436,000 × g for 15 min at 4 °C, after which pellets and supernatants were analysed by SDS–PAGE.

### Sample preparation for single-particle cryo-EM

Actin was reconstituted from lyophilised rabbit skeletal muscle actin powder (Cytoskeleton, Inc.) on ice to 10 mg/ml (5 mM Tris-HCl pH 8.0, 0.2 mM CaCl_2_, 0.2 mM ATP, 5% (w/v) sucrose, and 1% (w/v) dextran), then snap frozen with liquid nitrogen and stored at −80 °C. Reconstituted G-actin was centrifuged at 12,000 x g at 4 °C to remove aggregates. To polymerise F-actin, the supernatant was diluted to 1 mg/ml actin with HEPES-KME buffer with the addition of 0.2 mM ATP. Polymerised F-actin was stored at 4 °C for at least 24 h before use.

Immediately before use, F-actin was diluted in HEPES-KME buffer to a final concentration of 8 µM. Drebrin constructs were diluted to 72 µM or fascin-1 diluted to 15uM in the same buffer. 3 µl of F-actin was applied to glow-discharged EM grids and incubated for 1 min at room temperature. Excess buffer was manually blotted away, followed by the addition of 3 µl drebrin or fascin-1 solution and incubation for 1 min at room temperature. Excess solution was again blotted away, and a second 3 µl of drebrin or fascin-1 solution was applied. Grids were then transferred to an EM GP2 plunge freezer (Leica Microsystems), incubated for 1 min at 25 °C and 80% relative humidity, front blotted for 6 seconds and plunge-vitrified in liquid ethane. For preparation of F-actin with drebrin-E^135-355^, Au-flat™ gold EM grids with gold alloy film (1.2 µm holes, 1.3 µm spacing, Electron Microscopy Sciences) were used. For preparation of drebrin-E^135-256^, drebrin-E^256-355^, drebrin-E^1-431^ and fascin-1 with F-actin, C-flat™ carbon-coated copper EM grids (1.2 µm holes, 1.3 µm spacing, Protochips) were used.

### Single-particle cryo-EM data collection

Datasets of drebrin-E^1-431^, drebrin-E^135-256^ and drebrin-E^256-355^ with F-actin were collected using a Krios G3i operating at 300 kV, with a Gatan K3 direct electron detector and a Bioquantum Imaging Filter. Low-dose movies were collected automatically with EPU (ThermoFisher) software at 0.825 Å/pixel, in zero-loss imaging mode with a 20 eV energy-selecting slit. A defocus range of 0.7–2.5 μm was used, with a total dose of 64 e−/Å^2^ spread over 59 frames in electron counting mode (5 e- per pixel per second). Datasets of drebrin-E^135-355^ with F-actin were collected using a Krios III operating at 300 kV, with a Falcon 4i direct electron detector and a SelectrisX energy filter. Low-dose movies were collected automatically with EPU (ThermoFisher) software at 0.931 Å/pixel, in zero-loss imaging mode with a 10 eV energy-selecting slit. A defocus range of 0.7–2.5 μm was used, and the total movie dose was 45 e−/Å^2^ spread over 44 frames in electron counting mode.

### Cryo-EM image processing

Pre-processing steps were identical for all datasets. Movies were gain-corrected, motion-corrected, dose-weighted, and summed using RELIONv5’s implementation of MotionCor2^98^. CTF determination was then performed on micrographs using CTFFIND4^99^, then micrographs and metadata were imported into CryoSPARC v4.6.2^77^ for particle picking and initial refinements.

For all datasets, F-actin filaments were picked with an inter-box distance of 27.6 Å (corresponding to the helical rise of an actin protomer) using CryoSPARC’s filament tracer^77^ in template-free mode. Picked particles were curated based on normalised cross-correlation, power score, filament curvature, and filament sinuosity. For all datasets apart from F-actin with drebrin-E^256-355^ selected particles were extracted and 4× binned at this stage. For the dataset of F-actin with drebrin-E^256-355^ selected particles were extracted unbinned. Particles were then subjected to 2D classification in CryoSPARC v4.6.2^77^ to remove poor particles. Good classes were re-centred, 2× binned for all datasets apart from F-actin with drebrin-E^256-355^ (which was kept unbinned) and used for an initial 3D refinement in CryoSPARC v4.6.2^77^ using the helical refinement job with a helical symmetry order of 1. The 3D refinement reference volume was generated from PDB ID:7BT7^100^ (F-actin-Mg²⁺-ADP) using the molmap command in ChimeraX v1.9^101^ and then low-pass filtered to 20 Å resolution.

Duplicate particles were removed, and particle coordinates were converted into RELIONv5^98^.star files using PyEM (https://github.com/asarnow/pyem). Unbinned (drebrin-E^256-355^ with F-actin dataset) or 2× binned particles (drebrin-E^135-256^, drebrin-E^135-355^ and drebrin-E^1-431^ with F-actin datasets) were then re-extracted in RELIONv5^98^ and locally refined with C1 symmetry to correct potential orientational and translational offsets introduced during metadata conversion.

The drebrin-E^135-256^ and drebrin-E^256-355^ datasets were first examined to identify conformations of ABD1a, ABD1b, and ABD2. Initial explorative processing with unsupervised 3D classification revealed rough densities representing ABD1a and ABD1b in the drebrin-E^135-256^ dataset and ABD2 in the drebrin-E^256-355^ dataset. Based on ABD1 densities, masks and reference volumes for ABD1a and ABD1b were generated by trimming away all but ordered regions of ABD1 from the AlphaFold3-predicted models of drebrin-E F-actin, rigid-fitting these into density alongside F-actin protomer models (PDB:7BT7^100^, F-actin-Mg²⁺-ADP) then using the molmap command in ChimeraX^101^ and low-pass filtering the resulting density to 10 Å resolution. Then for the drebrin-E^135-256^ dataset, ABD1a or ABD1b-occupied actin protomer pairs were separated by focused supervised 3D classification using these references without alignment in RELIONv5^98^ and a T-value of 250. A soft classification mask was made to include both possible ABD1 conformations on the central actin protomer pair. Resulting ABD1a and ABD1b classes were subsequently each locally refined with C1 symmetry using a soft mask encompassing 35% of the filament.

For the drebrin-E^256-355^ dataset, our initial explorative processing with unsupervised 3D classification revealed ABD2 binds F-actin on every actin protomer (a stoichiometry of 1:1). Therefore, unbinned particles extracted in RELIONv5^98^ were not classified but instead subjected to two rounds of local refinement, each followed by CTF refinement and Bayesian polishing, followed by a final local refinement. Local refinements used a soft mask encompassing 35% of the filament.

A similar supervised classification strategy was applied to the drebrin-E^135-355^ and drebrin-E^1-431^ datasets. However, in addition to using the ABD1 reference maps described above for drebrin-E^135-256^ F-actin dataset processing, additional reference maps representing ABD2 bound to the upper, lower, or both actin protomers were created. These references were made from preliminary ABD2 models with actin protomers derived PDB:7BT7^100^ (F-actin-Mg²⁺-ADP) built into the final reconstruction from the drebrin-E^135-256^ dataset, using the molmap command in ChimeraX v1.9^101^ and low-pass filtering to 20 Å resolution. The corresponding reference densities were included within a soft-focused mask. Classes corresponding to ABD1a, ABD1b, or ABD2 bound F-actin in the correct register were selected and unbinned (except for the drebrin-E^1-431^ dataset) and re-centred particles extracted. Each class was then subjected to an initial local C1 3D refinement, followed by CTF refinement and Bayesian polishing, followed by a subsequent C1 local refinement. All local 3D refinements up to this point used a soft Z mask encompassing 35% of the filament. For the ABD1a and ABD1b classes, an additional focused local refinement was performed using a soft mask containing two actin protomers and the corresponding ABD1 density. Local refinements in RELIONv5^98^ used an initial angular search range of ± 3° and an initial offset range of ± 3 pixels.

Global resolutions from independently refined half-maps were estimated using the gold-standard Fourier shell correlation (FSC) 0.143 cutoff criterion (noise-substitution test–corrected^102^) and the soft masks applied during the final refinement steps. As an additional resolution measure, FSCs were calculated between maps and models in Phenix (v1.21.2-5419)^103^ and resolutions were estimated using the FSC 0.5 cutoff criterion. Local resolution was estimated in RELIONv5^98^. Displayed cryo-EM densities are locally sharpened with DeepEMhancer^104^ or local resolution filtering in RELIONv5^98^ as stated.

### Pseudo-atomic model building

Initial models of drebrin ABDs bound to F-actin were generated using AlphaFold3 by providing two copies of human drebrin-E^135-355^ together with six copies of human α-skeletal muscle actin and six copies each of ATP and Mg²⁺ as ligands. Drebrin ABD1a, ABD1b and ABD2 starting models were then produced by adjusting the position, then rigid fitting (using the fit-in-map tool in ChimeraX^101^) regions of the predicted drebrin model into cryo-EM density, then removing parts of the model which lacked corresponding density.

The actin chains in the predicted assembly were replaced by fitting the central two actin protomers from the Mg²⁺-ADP-Pi F-actin structure (PDB 8a2s^67^) into the maps and removing the Pi. Iterative rounds of model adjustment in Coot (v0.9.8.92)^105^ and real-space refinement in Phenix (v1.21.2-5419)^103^ were subsequently performed, using density maps sharpened according to local resolution in RELIONv5^98^.

### Cellular cryo-ET sample preparation

Gold H2 London finder EM grids with overlaid holey carbon (3 μm holes with 3 μm spacing, Quantifoil) were placed on the surface of 35 mm cell culture dishes (Nunc), then sterilised by UV treatment for 20 min on each side.

COS7 cells (ECACC 87021302) were plated in 6-well culture plates (NunclonTM Delta Surface, ThermoScientific 140675) and maintained at 37 °C in 5% CO_2_ in humidified air in Dulbecco’s Modified Eagle Medium + GlutaMax (Gibco, 31966-021) containing 10% heat-inactivated foetal bovine serum (Gibco BRL, 16140-071) and 100 I.U./ml penicillin/100 I.U./ml streptomycin (Sigma, P0781). Cells at >90% confluency were transfected in Opti-MEM I medium (Gibco, 51985-034) using Lipofectamine 2000 (Invitrogen, 11668027) and 4 μg of drebrin-eYFP DNA plasmid^8^ per well. Cells were left for 48 h at 37 °C and 5% CO_2_, released by trypsinisation, then re-plated on the pre-prepared EM grids overlaid on 35 mm cell culture dishes. After another 24 h, cells were vitrified using back-sided blotting (8–12 s blot time) on an automated Leica GP plunge freezing device operating at 37 °C and 75% humidity.

### Cryo-fluorescence microscopy and cryo-ET

Imaging of cellular drebrin-E-eYFP fluorescence was performed on unclipped grids maintained in the vitreous cryogenic state within a Linkam stage (CMS196) operating on a Nikon Eclipse E200 fluorescence microscope. Images were acquired using a 20× air-objective lens and stitched into a full EM grid map using NIS Elements software.

For cryo-ET, grids were then clipped and transferred to a ThermoFisher Titan Krios G3i operating with a Gatan K3 direct detector and BioQuantum energy filter with a 20 eV energy-selecting slit. Cryo-fluorescence grid maps were correlated with low-mag transmission cryo-EM grid maps by manual overlay, and target drebrin-E-eYFP transfected COS7 cell filopodia were identified. Tilt series were acquired at target sites using a dose-symmetric tilt^106^ scheme from −60 to +60° with 3° increments and a total dose of 120 electrons/Å^2^. Each tilt movie with three fractions was acquired in super-resolution mode using a sampling of 2.1 Å/physical pixel with a dose of 2.92 electrons/Å^2^.

Movies of individual tilts were gain-corrected, motion-corrected, dose-weighted, and summed using RELIONv5’s^98^ implementation of MotionCor2^98^. CTF determination was then performed on tilt images using CTFFIND4^99^. Tilt-series were aligned using AreTomo2^107^ wrapper within RELION v5^98^, with an estimated tomogram thickness off 200 nm, and bad tilts excluded by visual inspection. Tomograms were reconstructed using RELIONv5^98^ with dimensions of 4092 × 5760 × 2000 and binned x5 (to 10.5 Å/pixel final pixel size). Tomograms for display were denoised using IsoNET^108^.

#### Circular dichroism (CD) spectroscopy

CD spectra were acquired at 23 °C on Applied Photophysics Chirascan Plus spectrometer (Leatherhead, UK), using 0.5 mm path-length Quartz Suprasil rectangular cells. Data collection parameters were set to 2 nm spectral bandwidth, 1 nm step size, and 1 s instrument time per point. Spectra for drebrin^1-431^, drebrin^135-355^, drebrin^135-355 15^N were collected at concentrations of 0.170, 0.077, 0.107 mg/ml respectively. All spectra were adjusted by light-scattering, buffer subtracted, smoothed, corrected for protein concentration and expressed in terms of mean residue ellipticity (MRE). Secondary structure content was predicted using BeStSel web server^109^.

### Nuclear magnetic resonance (NMR)

NMR studies were performed using a Bruker Avance spectrometer operating at a proton frequency of 800 MHz. Heteronuclear single quantum correlation (^1^H-^15^N HSQC) experiments were conducted at 293.15 K using a ^15^N-labelled sample of drebrin-E^135-355^ (^15^N-drebrin-E^135-355^) at a final concentration of 50 μM in 17 mM NaH_2_PO_4_, 3 mM Na_2_HPO_4_, 50 mM NaCl, pH 6. NMR spectra were processed using TopSpin® (Bruker) and analysed with CcpNmr V3^110^. The assignments of the region between K173 and R317 were transferred from the Biological Magnetic Resonance Data Bank entries 52895 and 52729^72,94^. Order/disorder propensity was predicted with TALOS-N^73^, using the chemical shift perturbations of the NMR assignment. Data were analysed and displayed using Microsoft Excel and Prism 7 GraphPad (Dotmatics).

### Sequence alignment of drebrin’s actin-binding region

131 human drebrin orthologue canonical vertebrate protein sequences were downloaded from Ensembl release 115^111^. 93 sequences have a 1-to-1 relationship with human drebrin, and 38 fish sequences have a 1-to-many relationship with human drebrin. Sequences were iteratively aligned using MUSCLE software, with default parameters^112^, trimmed and curated to *Homo sapiens* drebrin residues 135–308 (common to human isoforms and containing both ABDs). The final sequence alignment retained drebrin sequences from 114 species, including *Homo sapiens*.

### Cell culture and confocal imaging

COS7 cells (ECACC 87021302) were plated in 24-wells plates (Nunclon^TM^ Delta Surface, ThermoScientific) with coverslips and maintained at 37 °C in 5% CO_2_ in humidified air in Dulbecco’s Modified Eagle Medium, no glucose (Gibco, 11966025) containing 10% foetal bovine serum (Gibco, A5256701) and 100 I.U./ml penicillin/100 I.U./ml streptomycin (Sigma, P0781). Media was changed to antibiotic-free media (recipe as above but with antibiotics) before transfection. Cells at >70% confluency were transfected using Lipofectamine 2000 (Invitrogen, 11668027) following the manufacturer’s instructions. A mix of 500 ng of DNA plasmid and 2 µl of Lipofectamine 2000 reagent diluted in Opti-MEM^TM^ (ThermoScientific) was used to transfect each well. Transfected cells were incubated for 18 h, then the Lipofectamine was removed by a change into fresh media containing antibiotics, followed by another 24-h incubation. Coverslips with transfected cells were fixed with 4% paraformaldehyde, then permeabilised with PBS containing 5% (w/v) bovine serum albumin and 0.2% (v/v) Triton X-100. Coverslips were then stained with phalloidin Alexa Fluor™ 568 (Invitrogen, A12380) and mounted on glass slides using ProLong™ Glass Antifade Mountant (Invitrogen, P36982).

Cells were imaged on a Nikon AXR inverted confocal microscope equipped with a 60× Plan Apo Lambda D oil-immersion objective. Filopodia, as finger-like protrusions extending beyond the cell membrane, were identified using FIJI v2.0.0^113^ plugin FiloQuant^114^, and those <1 µm in length (measured from tip to base), in addition to those located along concave regions of the membrane (retraction fibres), were excluded from final quantification. Cell perimeter distances were measured using FIJI. Colocalisation analyses were performed in CellProfiler v4.2.8^115^. Cellular objects were identified by automated segmentation, with saturated pixels excluded from analysis. Data points with Costes P values > 0.95 were averaged for plotting and statistical analysis.

### Reporting summary

Further information on research design is available in the Nature Portfolio Reporting Summary linked to this article.

## Supplementary information

Supplementary Information

Reporting Summary

Transparent Peer Review file

## Source data

Source Data

## Data Availability

Focused reconstructions of ABD1a, ABD1b and ABD2 bound to an F-actin protomer pair from the drebrin-E^135-355^-decorated F-actin data set are deposited in the Electron Microscopy Data Bank (EMDB) alongside corresponding atomic models deposited in the Protein Data Bank (PDB); EMD-55893, 9TG8 (F-actin-ABD1a); EMD-55894, 9TG9. (F-actin-ABD1b); EMD-55895, 9TGA (F-actin-ABD2). Source data are provided with this paper.
