# Supplementary material for: Structural mechanisms of drebrin-mediated F-actin network modulation
Source: Nat Commun. 2026 Jun 23;17:7894. doi: 10.1038/s41467-026-74543-6 (PMC13443164; doi:10.1038/s41467-026-74543-6)
Supplement: Supplementary file 1 [file 41467_2026_74543_MOESM1_ESM.pdf]

Structural mechanisms of drebrin-mediated F-actin network modulation

Supplementary Figures and Figure Legends

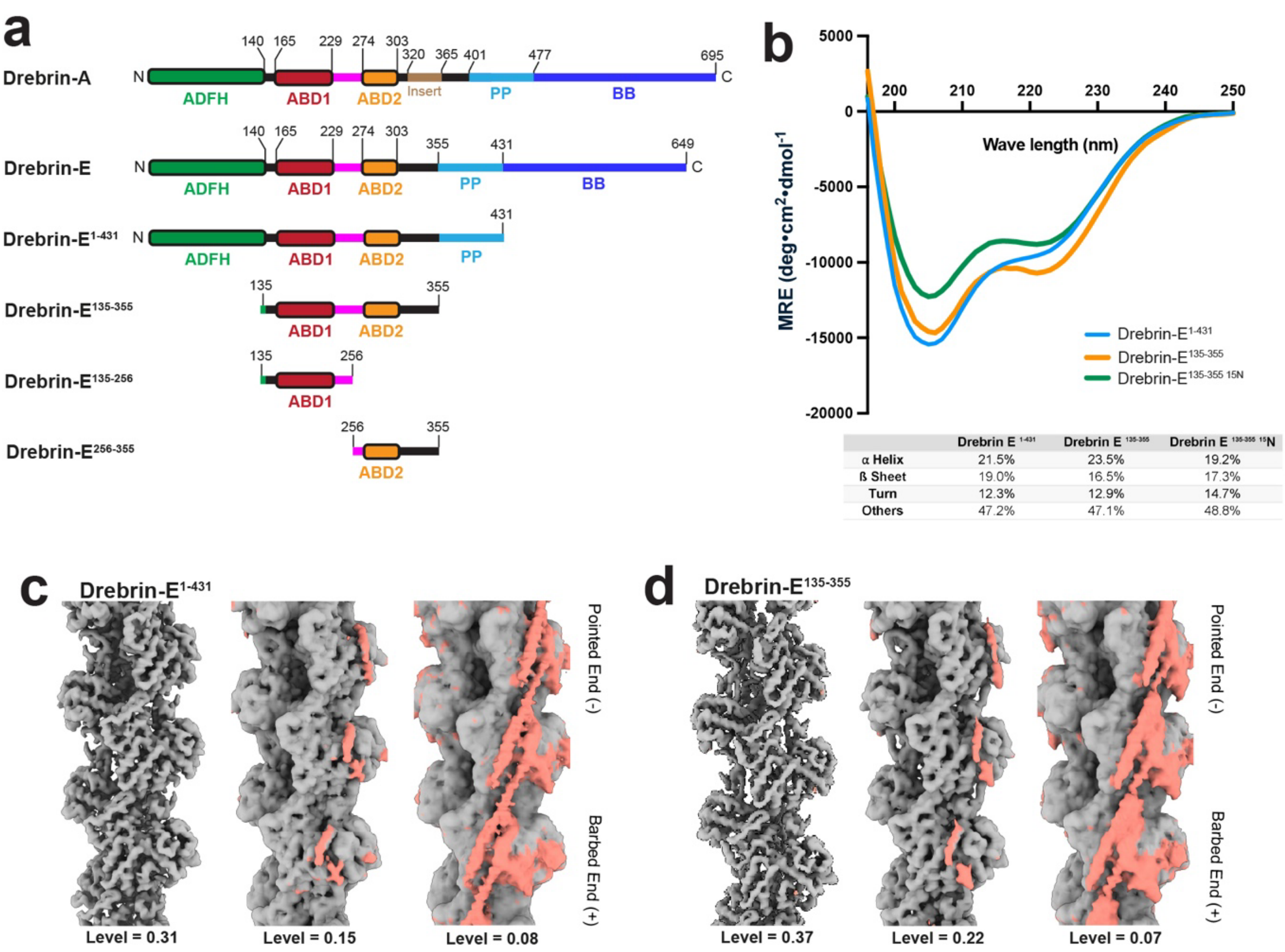

Supplementary Fig. 1. Initial analysis of drebrin-E constructs and F-actin interactions. a) Domain diagram for human drebrin-E and truncated drebrin-E constructs used in this study (human drebrin-A added for comparison). b) Circular dichroism spectra of drebrin-$E^{1\text{-}431}$, drebrin-$E^{135\text{-}355}$ and ^{15}N-drebrin-$E^{135\text{-}355}$ (used in NMR studies). The inset shows a table indicating secondary structure content prediction by the BeStSel server[1]. Initial cryo-EM reconstructions of F-actin with c) drebrin-$E^{1\text{-}431}$ and d) drebrin-$E^{135\text{-}355}$, before image classification. The Mg^{2+}-ADP bound F-actin model (PDB: 8a2t[2]) was fitted into the density and the colour Zone function in ChimeraX[3] used to colour density 5 Å from the fitted model in salmon, indicating density not accounted for by actin. Density threshold levels (indicated) were selected to demonstrate no significant differences between drebrin densities reconstructed from the two datasets.

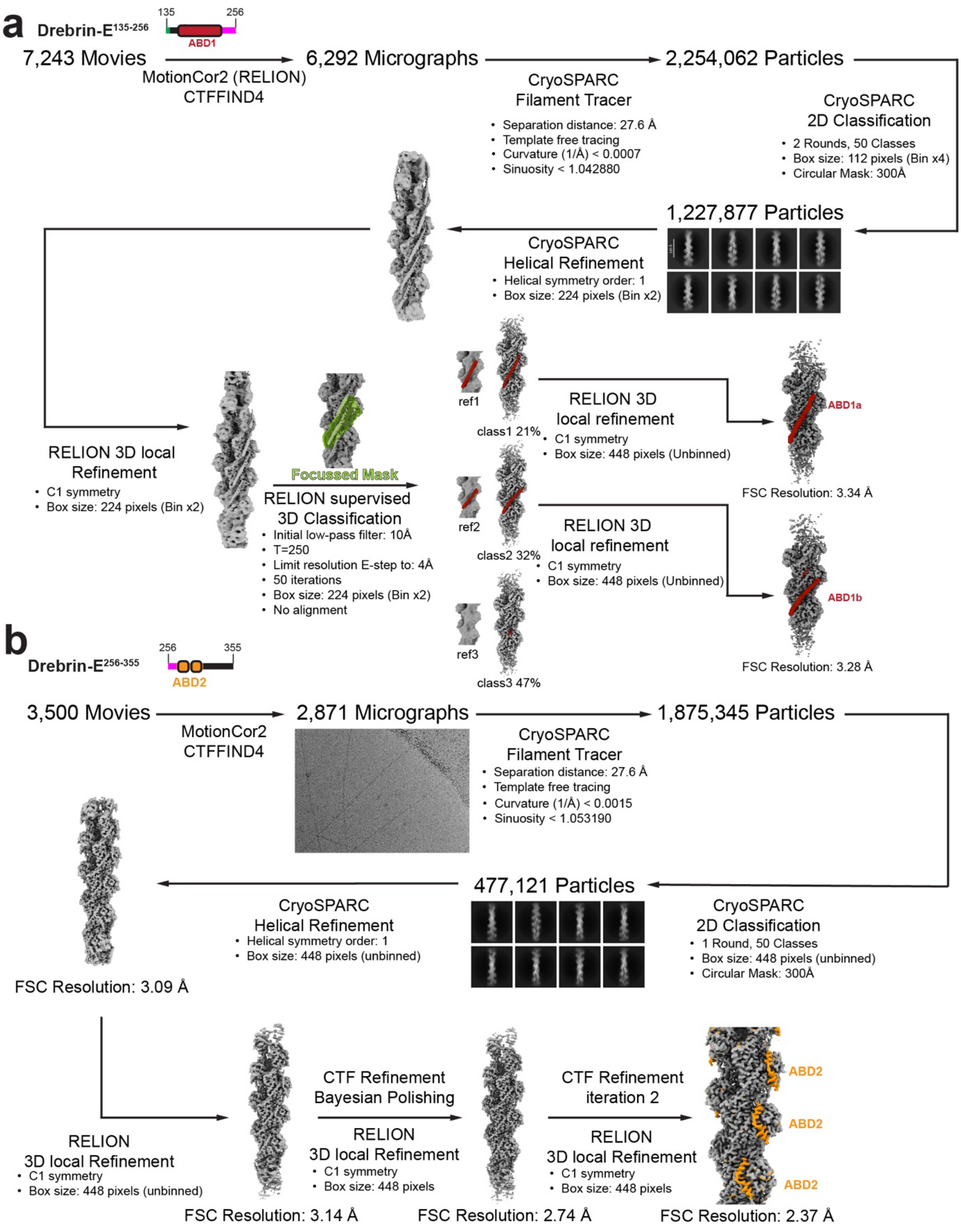

Supplementary Fig. 2. Image processing strategies and ABD1 or ABD2 reconstructions for F-actin decorated with drebrin-E$^{135\text{-}256}$ or drebrin-E$^{256\text{-}355}$ respectively. a) Schematic indicating the image and particle processing strategy for our dataset of F-actin decorated with a) drebrin-E$^{135\text{-}256}$ and b) drebrin-E$^{256\text{-}355}$ including initial steps in CryoSparc v4.6.2[4] and focussed classification and refinement steps in RELIONv5[5]. Cryo-EM reconstructions displayed are unsharpened densities. Class occupancies are rounded to one significant figure.

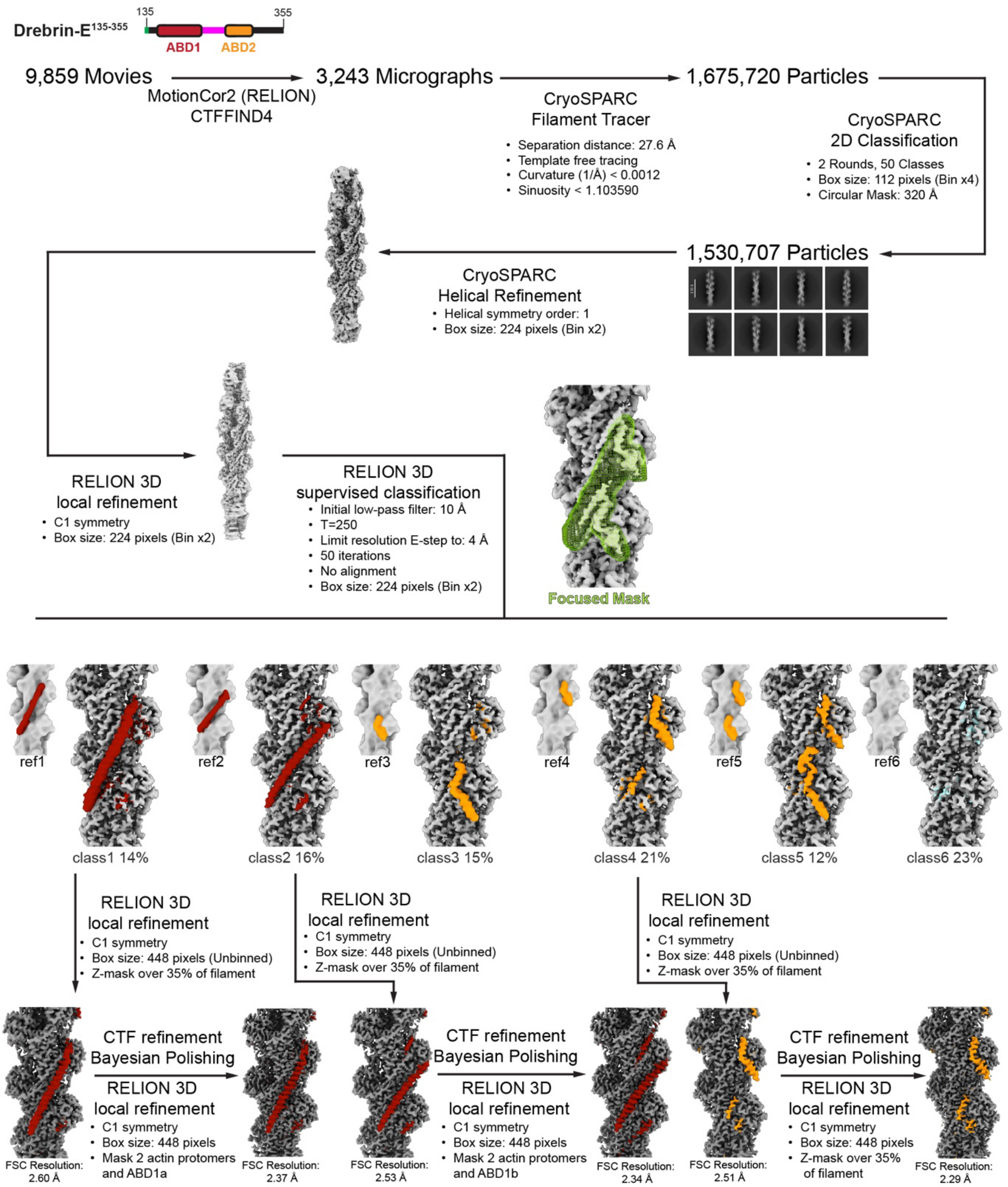

Supplementary Fig. 3. Image processing strategy for F-actin decorated with drebrin-E$^{135\text{-}355}$. Schematic indicating the image and particle processing strategy for our dataset of F-actin decorated with drebrin-E$^{135\text{-}355}$, including initial steps in CryoSparc v4.6.2[4] and focussed classification and refinement steps in RELIONv5[5]. Displayed final reconstructions were locally sharpened according to local resolution with RELIONv5[5], all prior displayed densities are unsharpened. Class occupancies are rounded to one significant figure.

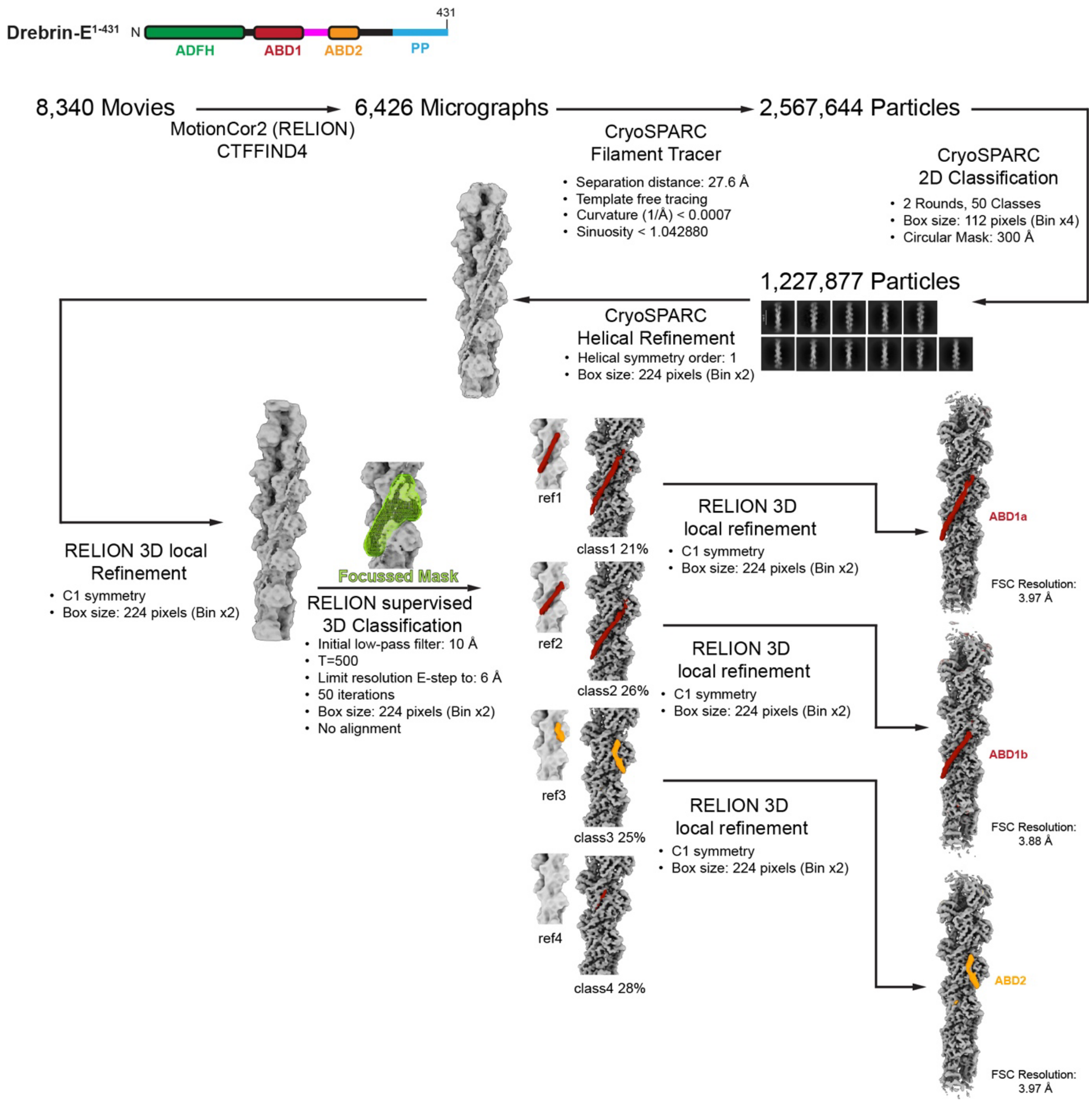

Supplementary Fig. 4. Image processing strategy for F-actin decorated with drebrin-E$^{1-431}$. Schematic indicating the image and particle processing strategy for our dataset of F-actin decorated with drebrin-E$^{1-431}$, including initial steps in CryoSparc v4.6.2[4] and focussed classification and refinement steps in RELIONv5[5]. Displayed final reconstructions were locally sharpened according to local resolution with RELIONv5[5], all prior displayed densities are unsharpened. Class occupancies are rounded to one significant figure.

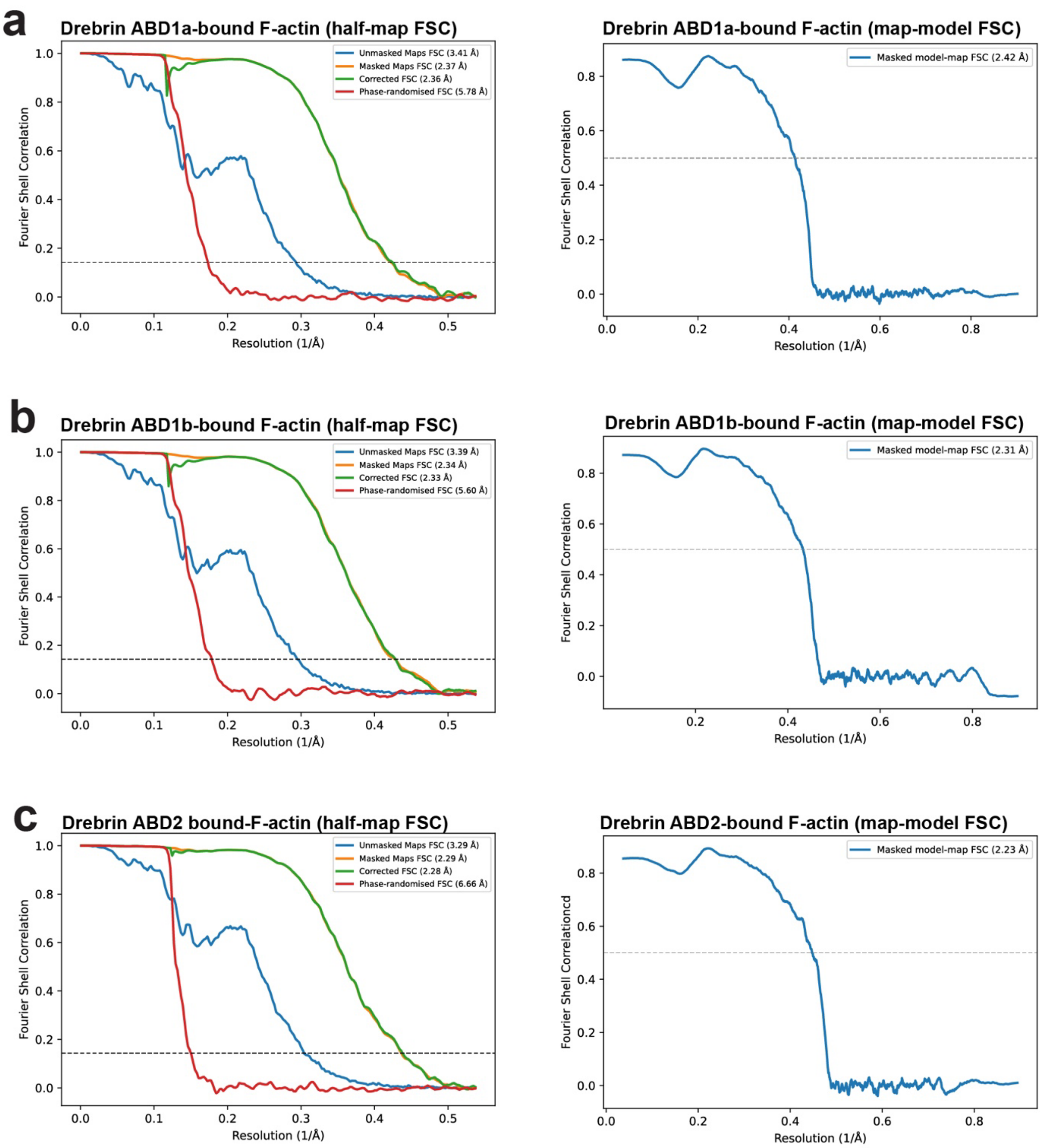

Supplementary Fig. 5. Resolution estimates for ABD1 and ABD2 reconstructions from the drebrin-$E^{135-355}$-decorated F-actin dataset. Gold-standard Fourier shell correlation (FSC) curves between independent half-maps (noise-substitution test corrected[6], calculated in RELIONv5[5], right) and map-model FSC curves (left) are shown for the focussed reconstructions of a) ABD1a, b) ABD1b and c) ABD2-decorated F-actin from our drebrin-$E^{135-355}$ dataset. Resolution estimates are shown above, based on the 0.143 cutoff criterion for half-map FSCs and the 0.5 cutoff criterion for map-model FSCs (cutoffs shown as dashed lines).

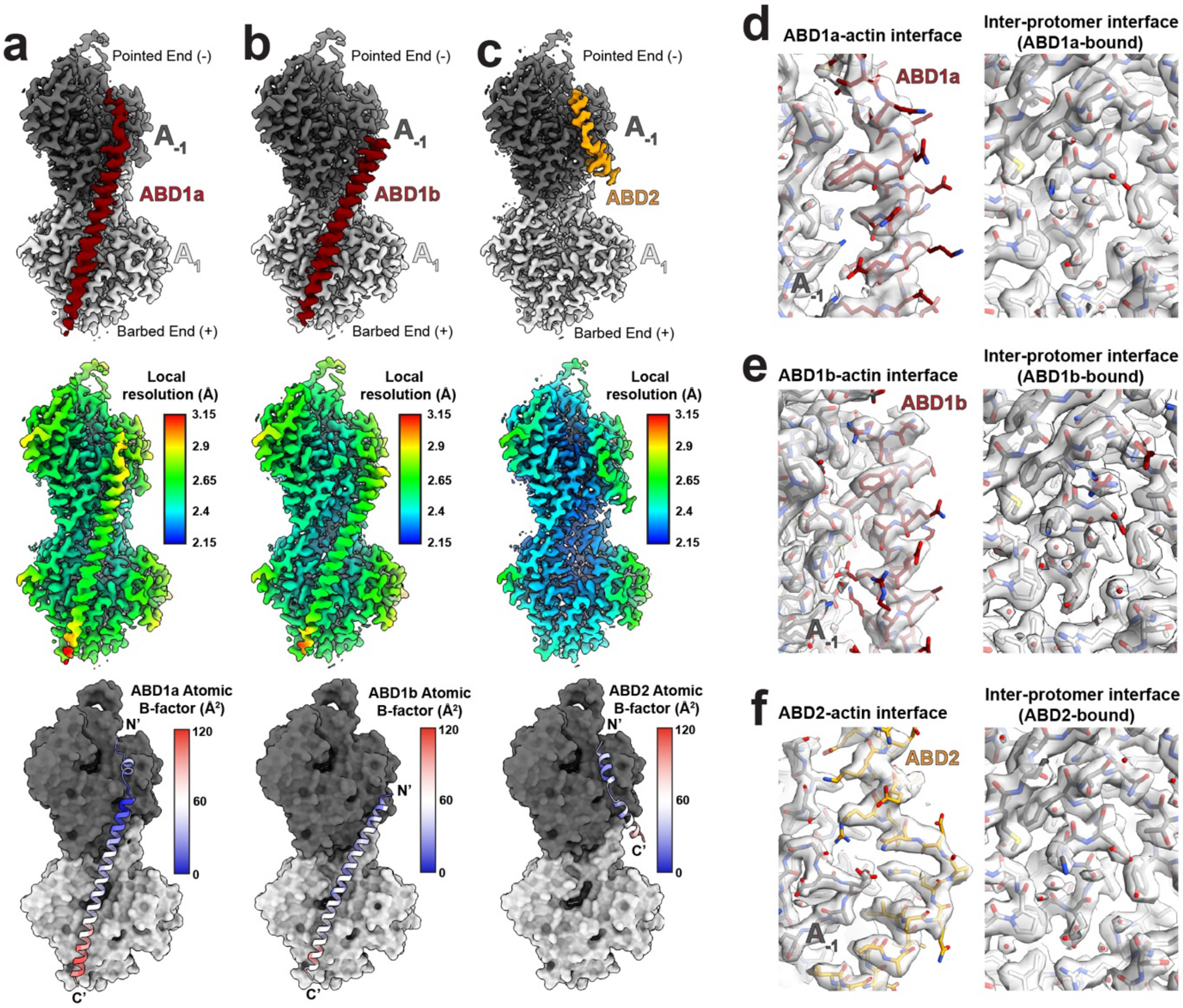

Supplementary Fig. 6. Local resolution estimates and map quality for ABD1 and ABD2 reconstructions from the drebrin-E$^{135\text{-}355}$-decorated F-actin dataset. Focussed reconstructions are shown of a) ABD1a, b) ABD1b and c) ABD2-decorated F-actin from the drebrin-E$^{135\text{-}355}$ dataset, coloured by domain (top panels) or the same view filtered and coloured by local resolution using RELIONv5[5] local resolution estimation (middle panels). For each reconstruction, the respective fitted model is shown in the bottom panels, with F-actin displayed as a surface and the drebrin ABD as a cartoon representation coloured by atomic B-factor. Example densities and fitted models for the drebrin-F-actin interface (left panels) and the central inter-protomer interface in F-actin (right panels) are shown for our d) ABD1a, e) ABD1b and f) ABD2-decorated F-actin reconstructions. All densities are sharpened to local resolutions determined using RELIONv5[5] and displayed at an RMS deviation threshold of 12.

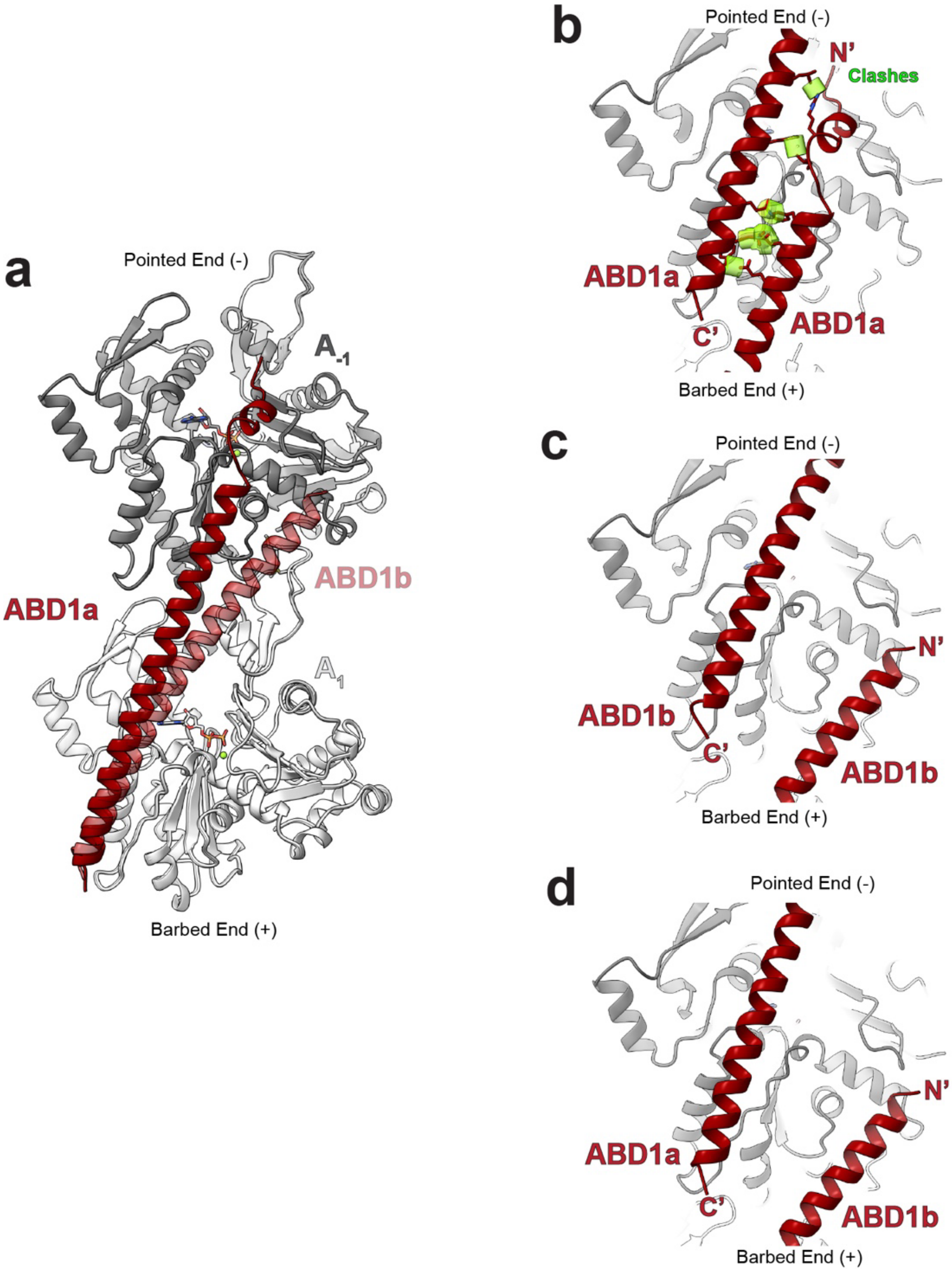

Supplementary Fig. 7. Definition of F-actin binding stoichiometry for ABD1 conformations. a) Models of F-actin with ABD1a and ABD1b (semi-transparent) superimposed via both bound actin protomers (A_1 and A_{-1}). b) Our ABD1a F-actin model is superimposed via protomer A_1 onto another ABD1a F-actin model with its protomer A_{-1} as a reference. c) Our ABD1b F-actin model is superimposed via protomer A_1 onto another ABD1b F-actin model with its protomer A_{-1} as a reference. d) Our ABD1a F-actin model is superimposed via protomer A_1 onto an ABD1b F-actin model with protomer A_{-1} as a reference. Clashes between ABD1s after this superimposition were detected with default settings using ChimeraX's 'clashes' tool[3]) and displayed as transparent green density.

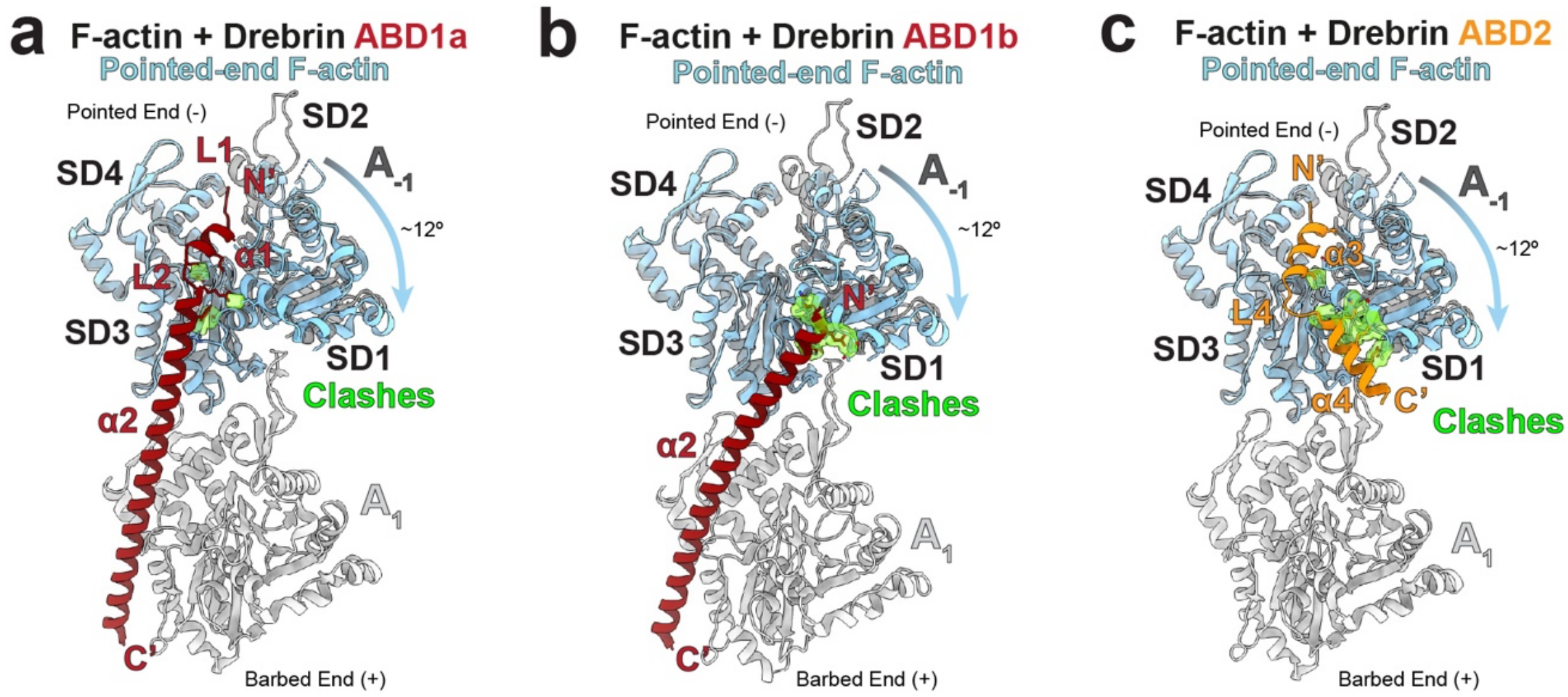

Supplementary Fig. 8. The drebrin binding site is lost in the 'twisted' conformation of actin found in G-actin or at pointed ends. Superimposition of the 'twisted' conformation ultimate pointed end F-actin protomer (light blue, PDB: 9FJO[7]) via SD3 & SD4 onto SD3 & SD4 of actin protomer A_{-1} in our a) ABD1a, b) ABD1b and c) ABD2 models (transparent dark grey). The ~12º rotation of SD1 & SD2 relative to SD3 & SD4 in the 'twisted' conformation is indicated and resultant clashing regions of this conformation (detected with default settings using ChimeraX's 'clashes' tool[3]) with drebrin ABDs displayed as transparent green density. The penultimate protomer from PDB: 9FJO (in the 'flattened' conformation) is also shown in light grey.

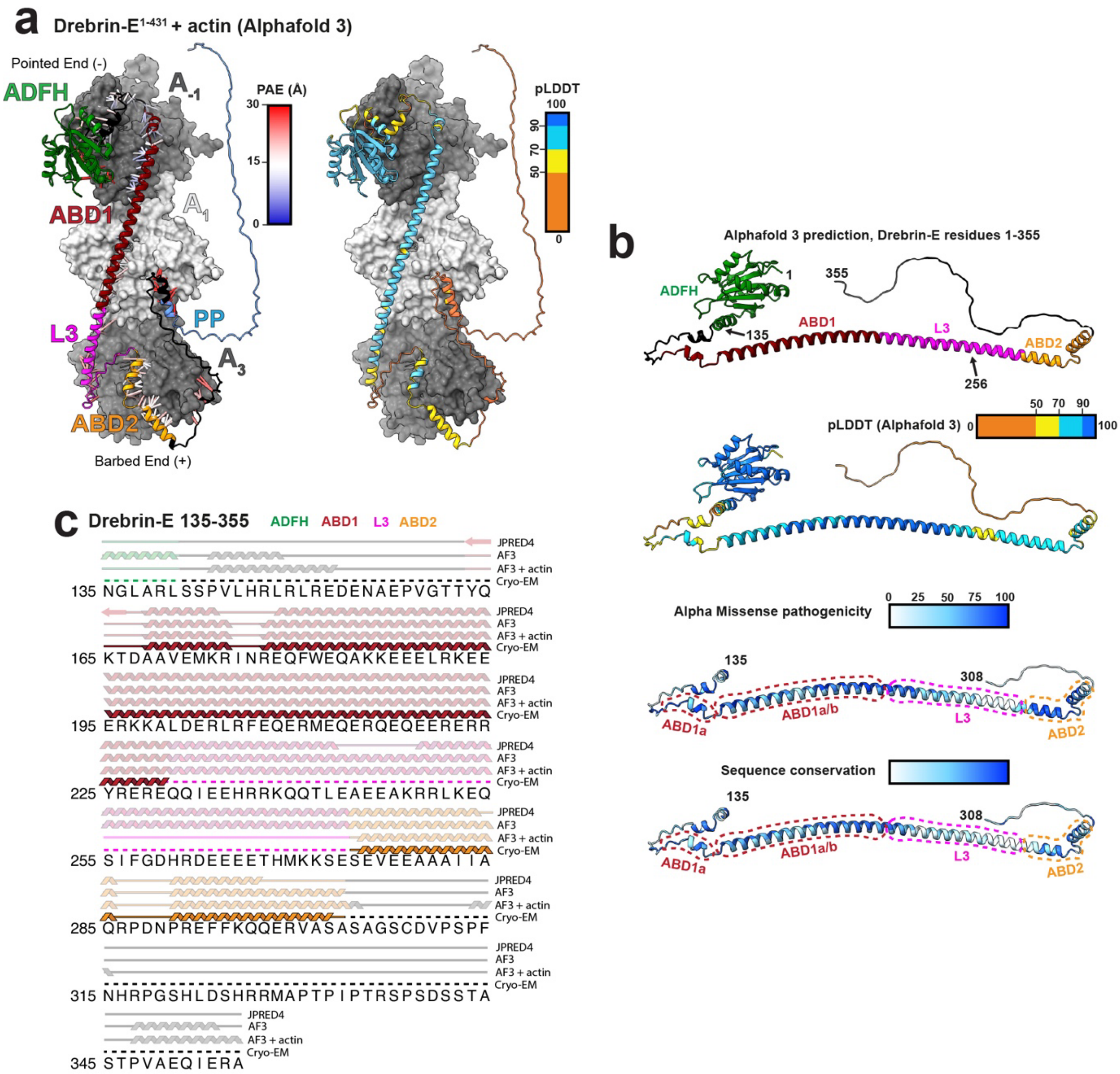

Supplementary Fig. 9. Computational predictions of drebrin structure compared to experimental cryo-EM structures. a) Structural prediction of a drebrin-E$^{1\text{-}431}$ molecule with 6 Mg^{2+}-ADP-bound F-actin protomers with Alphafold3[8] was performed. The drebrin-E$^{1\text{-}431}$ molecule with its three interacting actin protomers are shown, coloured by domain (left) or pLDDT confidence score (right). The predicted aligned error (PAE) for this prediction is shown on the left panel on as pseudobonds coloured according to the shown PAE scale (expected position error in Å). b) Structural prediction of drebrin-E residues 1-355 in the absence of actin with Alphafold3[8], coloured by domain with amino acids at construct boundaries indicated (top panel) or pLDDT confidence score (bottom panel). In the bottom two panels, the above Alphafold3 prediction is trimmed to residues 135-308 and coloured by AlphaMissense[9] pathogenicity scores or sequence conservation calculated from the 114 species sequence alignment introduced in Fig. 2, with ABDs and L3 identified by cryo-EM indicated with dashed lines. c) The sequence for drebrin-E amino acids 135-355 is shown, with secondary structure predicted by JPred4[10] or Alphafold3[8] (in the presence or absence of 6 F-actin protomers) compared with that found by cryo-EM (ABD1a and ABD2) shown above. Predictions are shown as faded (compared to experimental data) and dashed lines indicate regions unresolved due to flexibility in our cryo-EM reconstructions.

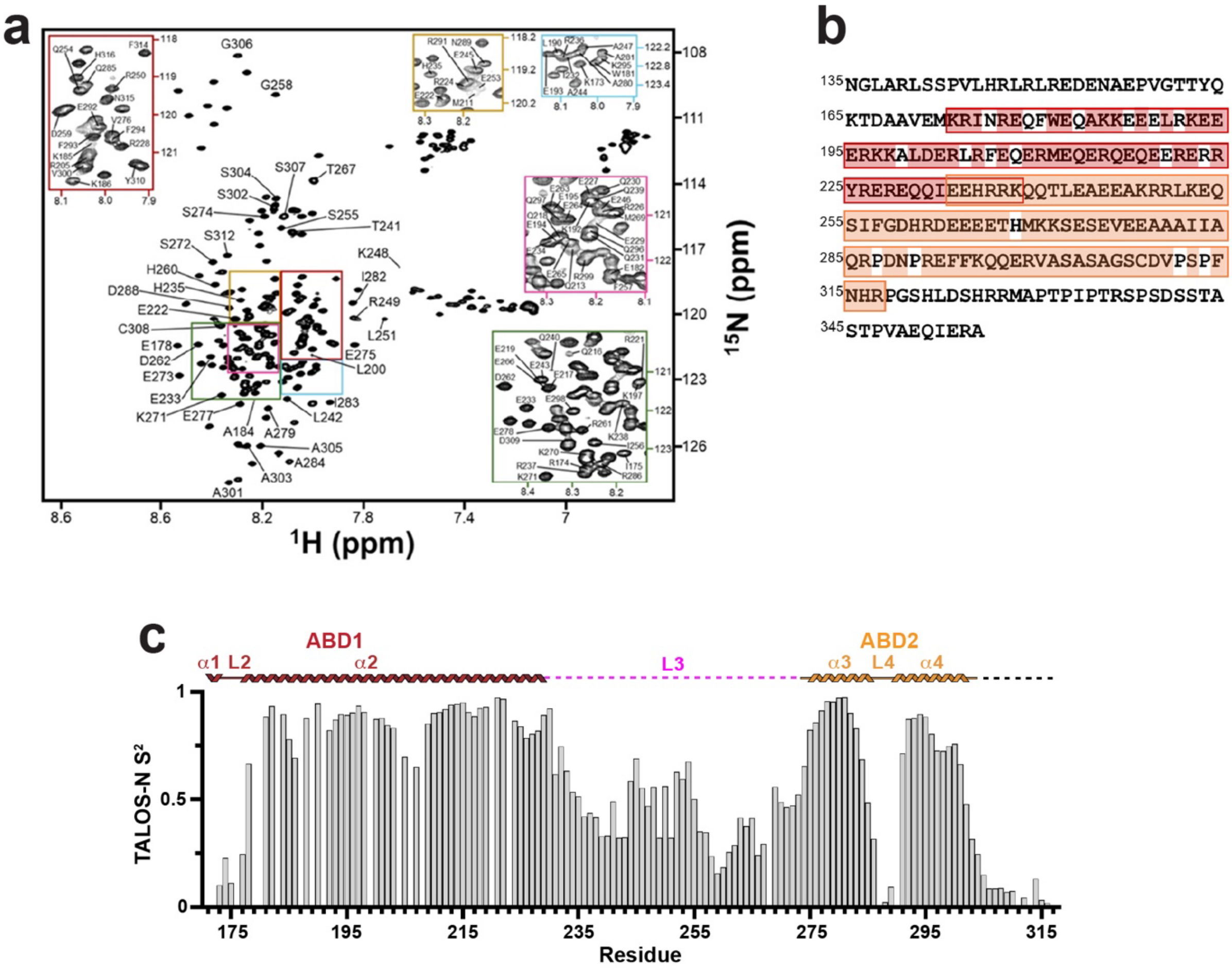

Supplementary Fig. 10. NMR of ^{15}N-drebrin-E$^{135\text{-}355}$. a) Assigned ^{1}H-^{15}N HSQC NMR spectrum of ^{15}N-drebrin-E$^{135\text{-}355}$. The coloured insets are zoomed in crowded regions of the spectrum. b) Assignment transfer of ^{15}N-drebrin-E$^{135\text{-}355}$. The sequence shown in black corresponds to the protein construct used in this study. Red boxes indicate residues covered by previous NMR studies of the drebrin fragment termed single-alpha helix (SAH; residues 173-238; BMRB ID 52729[11] with successfully transferred assignments highlighted in red. Orange boxes indicate residues covered by NMR studies of the Drebrin fragment D233 (residues 233-317; BMRB ID 52895[12]) with successfully transferred assignments highlighted in orange. Overall assignment transfer achieved 94% coverage for the region spanning 173-317. c) Predicted order parameter (S2) for drebrin-E residues 173-317 obtained from the Random Coil Index (RCI) using TALOS-N[13]. Residues with RCI-S^2 values close to 1 are considered structured, while those with $S^2 < 0.6$ are likely to be dynamic. The experimentally observed secondary structure elements (SSEs) derived from our cryo-EM data are displayed above the corresponding residue numbers.

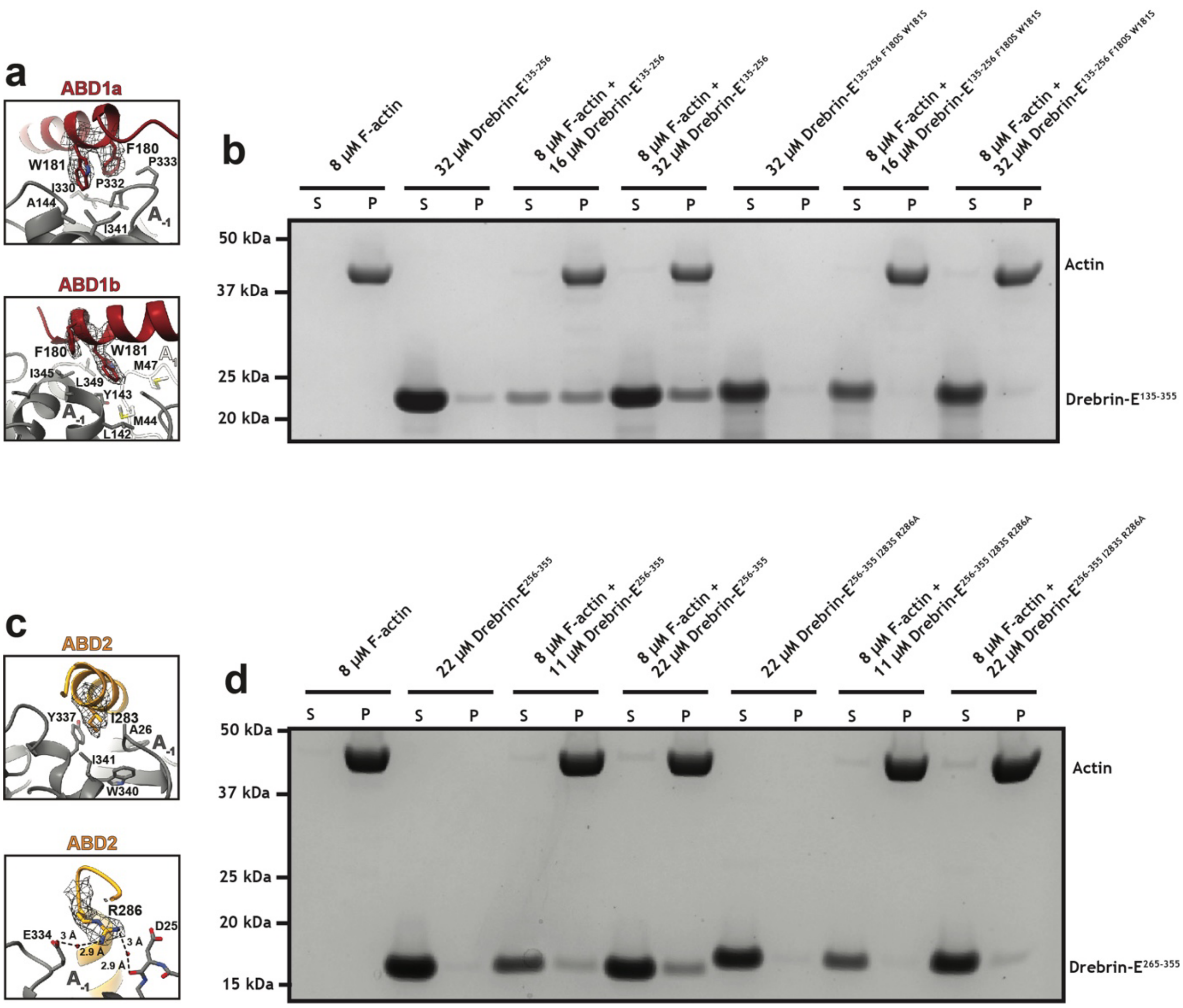

Supplementary Fig. 11. Mutations to key conserved residues at the ABD1 or ABD2 interface with F-actin disrupt binding by co-sedimentation assay. a) Zoomed images of drebrin-E residues W181 and F180, shown in mesh cryo-EM density (sharpened with DeepEMhancer[14]), and interacting residues on F-actin for ABD1a (top) and ABD1b (bottom). b) F-actin co-sedimentation assay with drebrin-E$^{135\text{-}256}$ or the drebrin-E$^{135\text{-}256\ F180S\ W181S}$ mutant at indicated concentrations. c) Zoomed images of drebrin-E ABD2 residues I283 (top) and R286 (bottom), shown in mesh cryo-EM density (sharpened with DeepEMhancer[14]) alongside interacting residues on F-actin and waters. Hydrogen bonding distances are indicated. d) F-actin co-sedimentation assay with drebrin-E$^{256\text{-}355}$ or the drebrin-E$^{256\text{-}355\ I283S\ R286A}$ mutant at indicated concentrations. S= supernatant and P = Pellet. F-actin and bound protein co-sediment in the pellets.

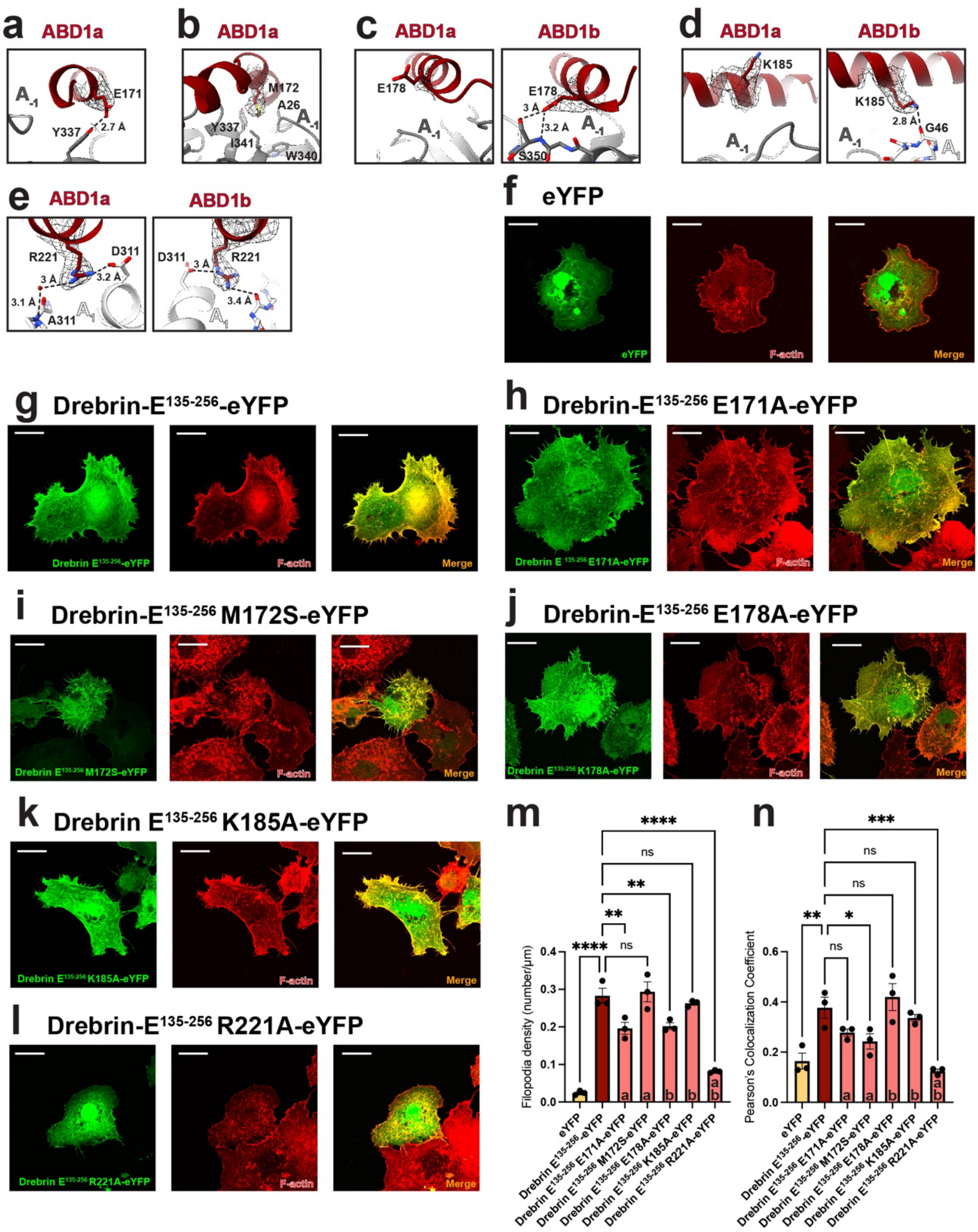

Supplementary Fig. 12. Mutations to F-actin interacting residues reduces ABD1's ability to induce filopodia in COS7 cells. Zoomed images of our ABD1 F-actin models focussed on drebrin-E amino acids a) E171, b) M172 (both residues are in helix-α1 which is only ordered/interacting with F-actin in the shown ABD1a conformation), c) E178, d) K185 or e) R221 (left panels; ABD1a, right panels ABD1b). Cryo-EM density for each mutated side chain is shown in mesh (sharpened with DeepEMhancer[14]). Interacting residues on F-actin and hydrogen bonding distances are indicated. Representative confocal microscopy images of COS7 cells transfected with f) eYFP, g) drebrin-E$^{135\text{-}256}$-eYFP, h) drebrin-E$^{135\text{-}256}$-E171A-eYFP, i) drebrin-E$^{135\text{-}256}$-M172S-eYFP, j) drebrin-E$^{135\text{-}256}$-E178A-eYFP, k) drebrin-E$^{135\text{-}256}$-K185A-eYFP and i) drebrin-E$^{135\text{-}256}$-R221A-eYFP. YFP and phalloidin-AF568 fluorescence channels are shown. Scale bars = 30μm. m) Quantification of filopodia density (number of filopodia per unit length of cell perimeter) in

COS7 cells. For each condition, 3 independent transfections were performed then measurements taken from 10 cells from each transfection. Each data point represents the mean of 10 cells from a single independent transfection. Error bars represent ± standard error of the mean (SEM) for each condition. Ns = not significant, ** = p<0.01, **** = p<0.0001, 1-way ANOVA. n) Quantification of colocalisation between phalloidin-AF568 (F-actin) and eYFP fluorescence for cells transfected with drebrin-$E^{135-256}$ and point mutants. For each condition, 3 independent transfections were performed then measurements taken from cell objects detected by CellProfiler[15]. Each data point represents the mean of the measurement from all the objects in a single independent transfection. Error bars represent ± standard error of the mean (SEM) for each condition. Ns = not significant, * = p<0.05, ** = p<0.01, 1-way ANOVA. In bar graphs, the annotations ‘a’, ‘b’ and ‘ab’ within bars indicates mutations to drebrin residues interacting with F-actin in ABD1a, ABD1b or both conformations respectively.

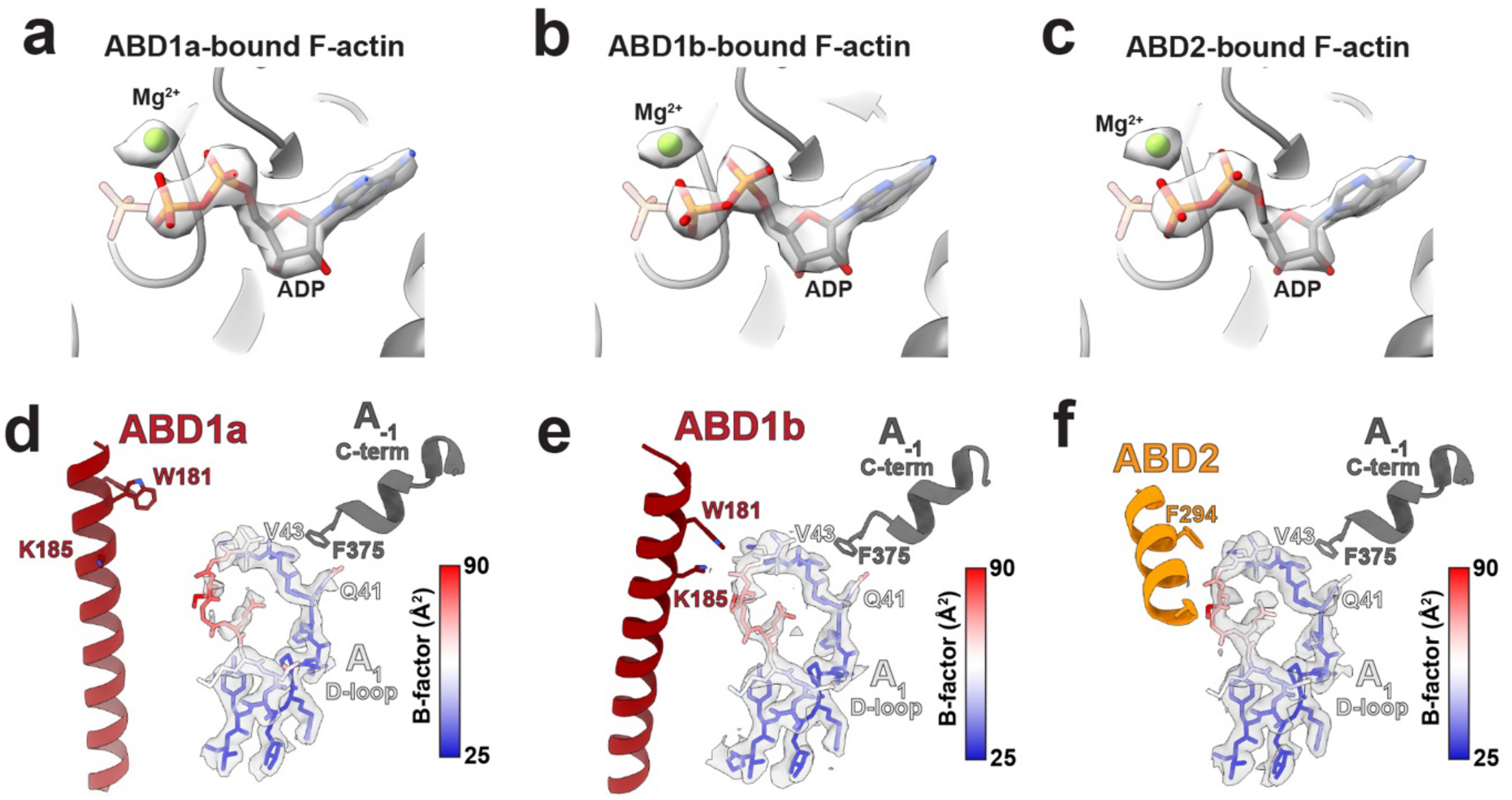

Supplementary Fig. 13. Drebin-E ABDs are bound to Mg^{2+}-ADP F-actin and ABD1b and ABD2 stabilise F-actin's D-loop. Zoomed density and model for Mg^{2+}-ADP associated with the a) ABD1a, b) ABD1b and c) ABD2-bound F-actin reconstructions. The theoretical position of the γ-phosphate if ATP was bound is shown as a faded model. Models for d) ABD1a e) ABD1b and f) ABD2-bound F-actin, showing only the ABD and D-loop and C-terminus in proximal actin protomers. The D-loop only is coloured by atomic b-factor. Local-resolution sharpened density for the D-loop is displayed at matching thresholds (RMS level 10) for all three reconstructions. In all models, key interacting side chains at the inter-protomer and drebrin-D-loop interface are shown (particularly those remodelled in the drebrin-bound state).

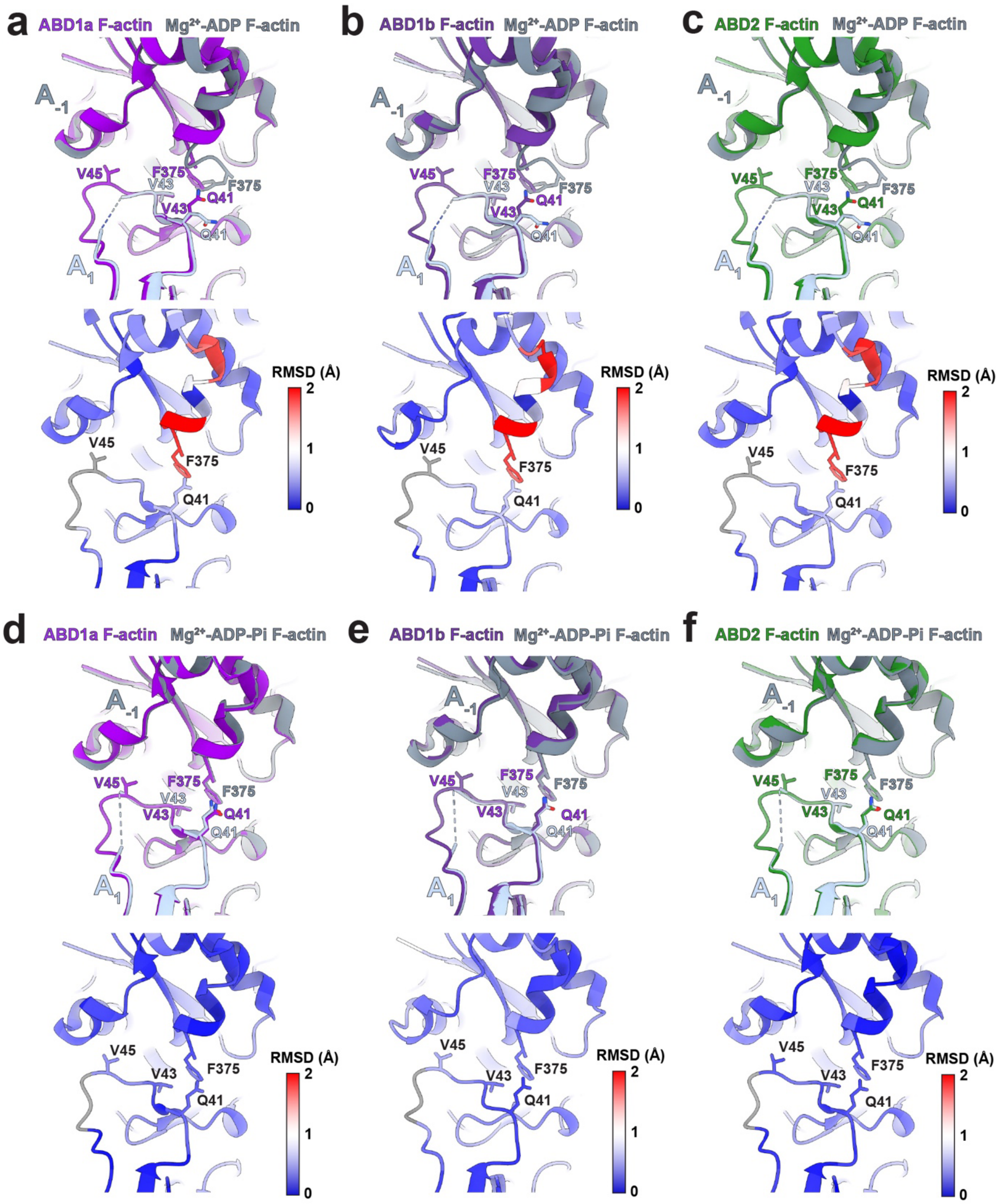

Supplementary Fig. 14. Drebin-E ABDs stabilise a D-loop and C-terminus conformation seen in Mg^{2+}-ADP-Pi-bound F-actin. Superimpositions of Mg^{2+}-ADP F-actin alone (PDB: 8A2T[2]) with a) drebrin ABD1a-bound, b) drebrin ABD1b-bound and c) drebrin ABD2-bound Mg^{2+}-ADP F-actin (top) with corresponding Cα Root Mean Square Deviations (RMSDs, bottom). The view shown is focussed on the D-loop and C-terminus. The same views after superimpositions of our d) ABD1a Mg^{2+}-ADP F-actin, e) ABD1b Mg^{2+}-ADP F-actin and f) ABD2 Mg^{2+}-ADP F-actin models with the model of Mg^{2+}-ADP-Pi-bound F-actin alone (PDB: 8a2s[2]). Cα RMSDs corresponding to the superimpositions are shown below. In all models, key interacting side chains at the inter-protomer and drebrin-D-loop interface are shown (particularly those remodelled in the drebrin-bound state).

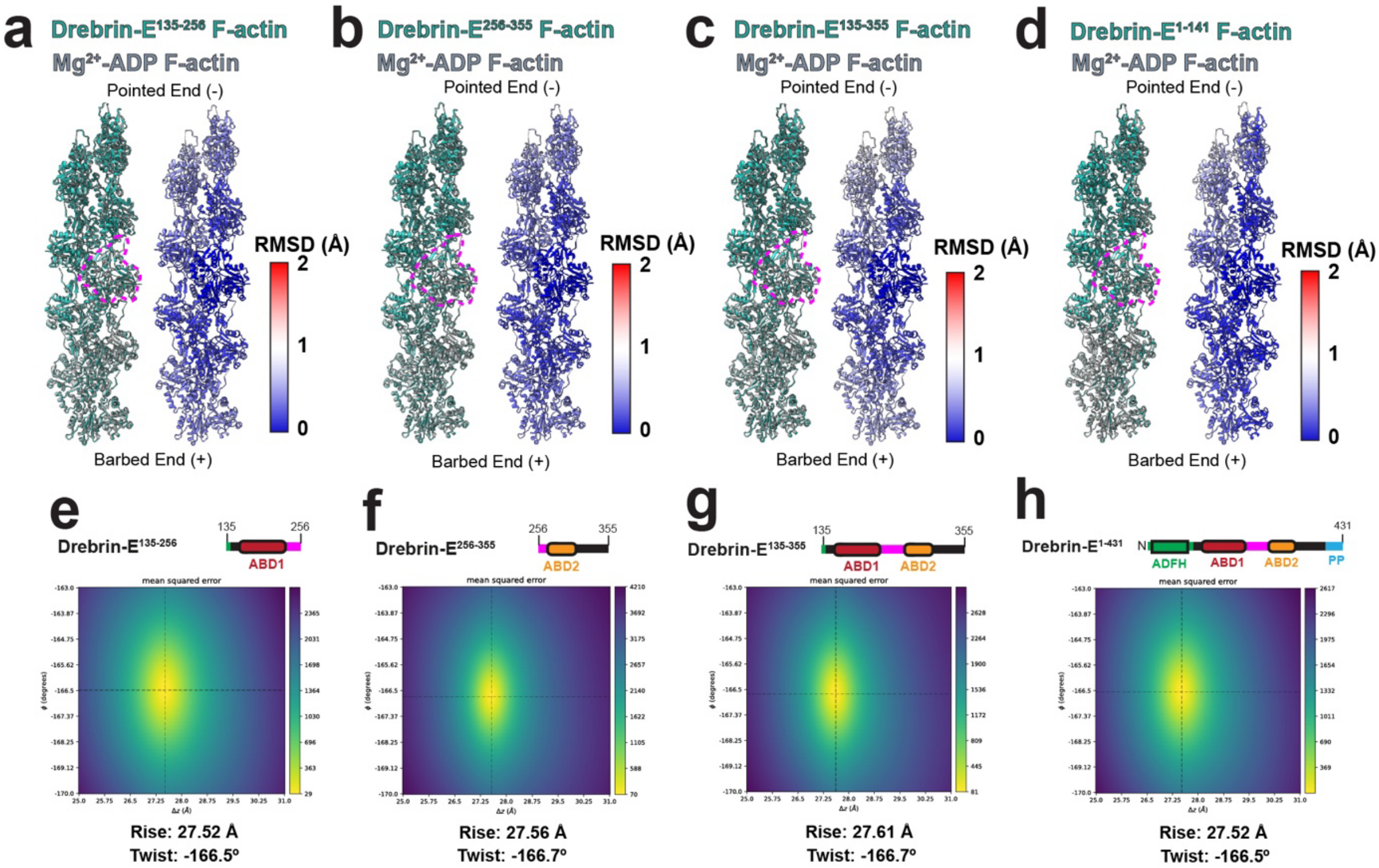

Supplementary Fig. 15. Drebrin does not modify F-actin helical rise and twist. 9 protomer atomic models were built by fitting Mg^{2+}-ADP F-actin protomers (PDB: 8A2T[2]) into drebrin-E$^{135-256}$, drebrin-E$^{256-355}$, drebrin-E$^{135-355}$, drebrin-E$^{1-431}$ or Mg^{2+}-ADP F-actin alone (EMD-15106[2]) reconstructions. A central single protomer of these a) drebrin-E$^{135-256}$, b) drebrin-E$^{256-355}$, c) drebrin-E$^{135-355}$ or d) drebrin-E$^{1-431}$ nine protomer F-actin models was then superimposed on a single central protomer of the Mg^{2+}-ADP nine protomer F-actin model, to indicate any difference in the helical parameters of repeating protomers along the F-actin axis. The superimposed protomers are indicated with dashed magenta lines. Cα RMSDs are calculated and displayed to the right of the corresponding superimpositions. Helical twist and rise calculated from reconstructions of e) drebrin-E$^{135-256}$, f) drebrin-E$^{256-355}$, g) drebrin-E$^{135-355}$ and h) drebrin-E$^{1-431}$-decorated Mg^{2+}-ADP F-actin in CryoSPARC[4], before any focussed classification of drebrin ABD occupancy. Drebrin-E construct domain schematics are shown above.

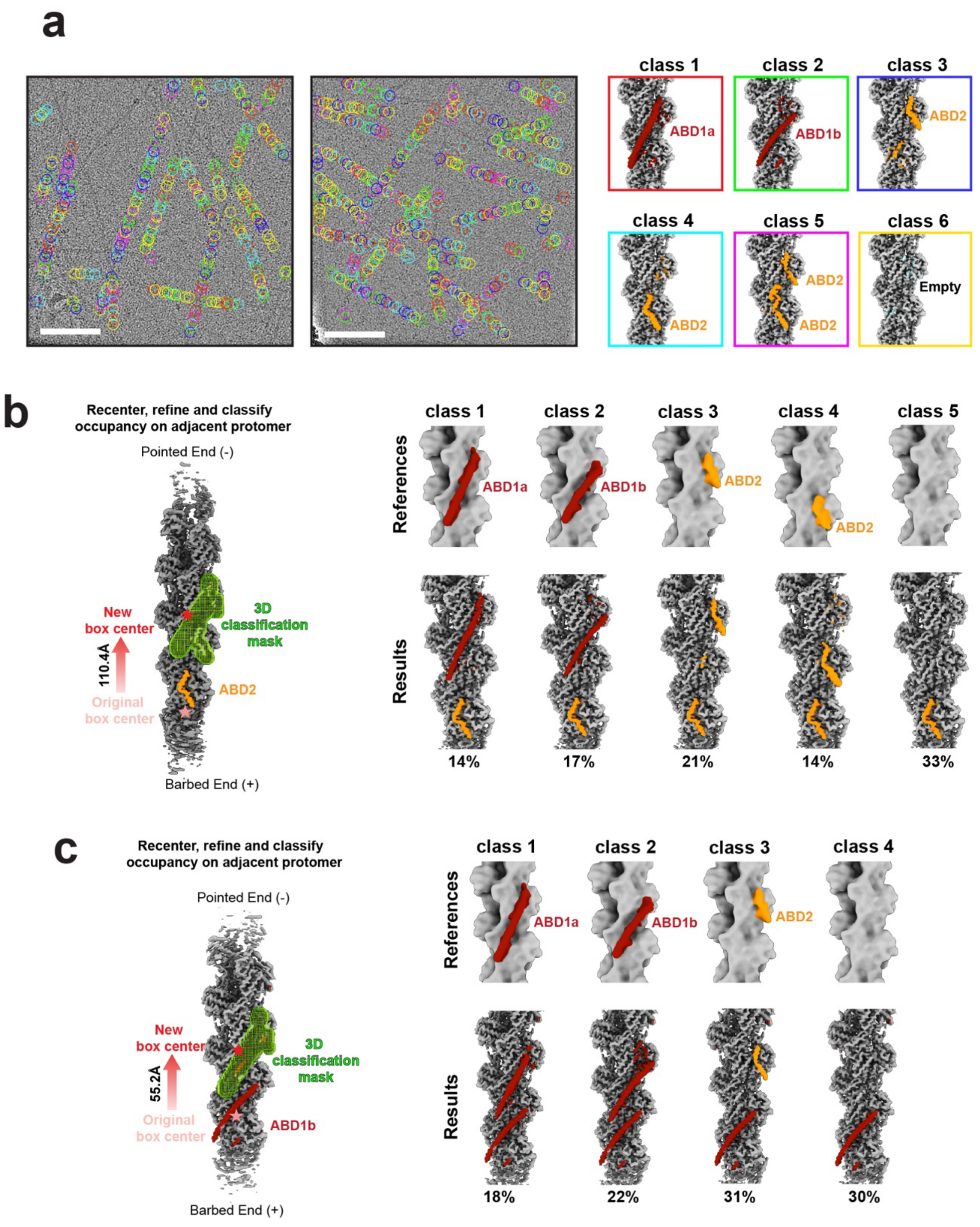

Supplementary Fig. 16. There is no clear spatial relationship between ABDs on an actin filament. a) 3D classes representing different ABD/conformation occupancies (right hand panels) are plotted back to their locations in micrographs (left two panels) of our drebrin-E$^{135\text{-}355}$-decorated F-actin dataset. Colours of boxes around classes on the right correspond to the colours used to illustrate the positions of their constituent particles in the micrographs. Scale bars = 80 nm. Schematic showing classification strategy to assess occupancy of ABDs at F-actin substrate sites adjacent to b) ABD2 or c) ABD1b. Density from the refined reconstruction is shown after re-extracting/recentring 110.4 Å (for ABD2) or 55.2 Å (for ABD1b) towards the pointed end of the original reconstructed ABD2 or ABD1b-bound class (new box centre, red, original box centre, faded red). The focussed 3D classification mask for ABD occupancy is shown in green. Panels to the right show the corresponding supervised 3D classification class

references and results, derived from the strategy indicated to the left. Density is coloured according to molecular identity (F-actin = grey, ABD1 = maroon and ABD2 = orange) and class occupancy % is shown below the results. Class occupancies are rounded to one significant figure.

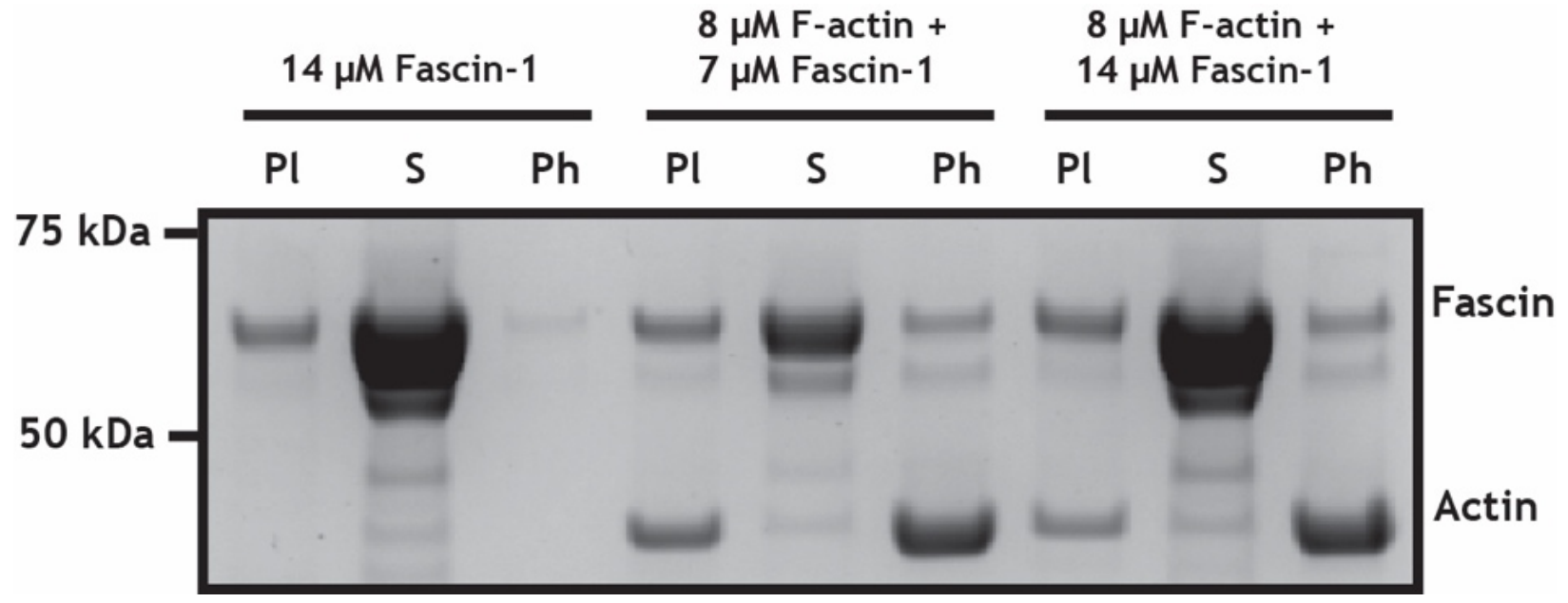

Supplementary Fig. 17. Low-speed co-sedimentation assays of F-actin incubated with fascin-1. Low-speed co-sedimentation assay, with F-actin and fascin-1 as a positive control, where any bundled F-actin sediments in the low-speed pellet (Pl), unbundled F-actin sediments in the high-speed pellet (Ph) and soluble protein remains in the supernatant (S). Concentrations used are indicated.

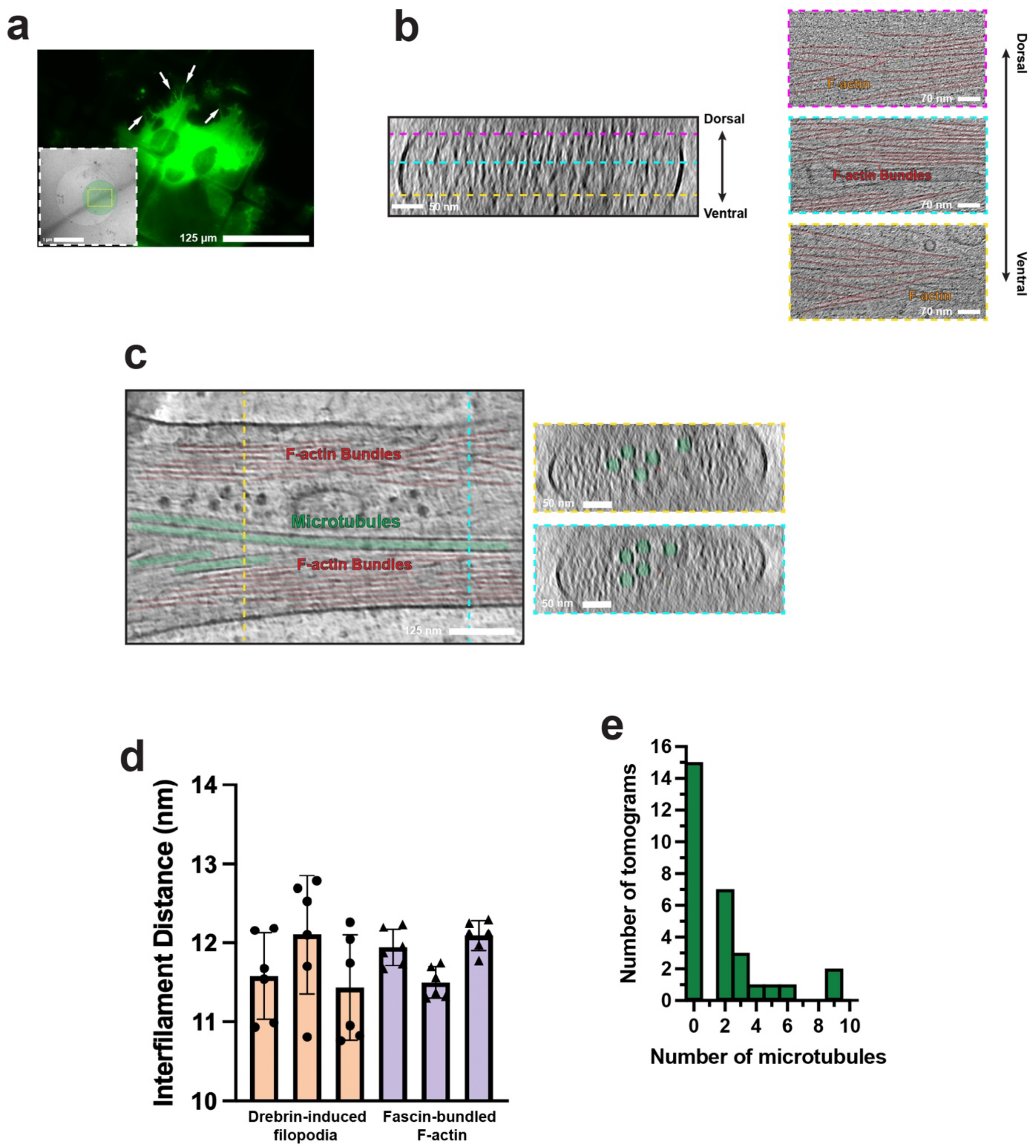

Supplementary Fig. 18. Characterisation of F-actin networks in drebrin-eYFP induced filopodia in COS7 cells by cryo-ET. a) Light microscopy image of vitrified drebrin-E-eYFP-transfected COS7 cells on an EM grid surface, with arrows indicating drebrin-E-eYFP-overexpressing filopodia. Inset shows a low-mag TEM image of a drebrin-E-eYFP- overexpressing filopodium extending over a hole in the carbon, with the yellow rectangle and green circle indicating the imaged and exposed areas respectively where a tilt series was collected. b) Transverse cryo-electron tomographic section through a drebrin-E-eYFP-overexpressing filopodium (left) with lines indicating the positions of corresponding longitudinal slices through the same tomogram (right) moving from ventral to dorsal positions. c) Longitudinal cryo-electron tomographic section through the medial portion of another drebrin-E-eYFP-overexpressing filopodium (left panel). Transverse sections through the same filopodium, at locations corresponding to the coloured dashed lines in the left panel are shown to the right. In panels b and c microtubules are false coloured in green and an indicative portion of F-actin in

red in longitudinal sections. d) Quantification of distance in nm between actin filaments in drebrin-transfected filopodia compared to F-actin incubated with fascin-1. Three tomograms were analysed for each condition. Each data point represents the mean centre-to-centre distance from the central actin filament to the six neighbouring filaments within a bundle. Five measurements were taken from each tomogram at 85-nm intervals. e) Histogram showing the number of microtubules found in 30 tomographic volumes of drebrin-E-eYFP-overexpressing filopodia.

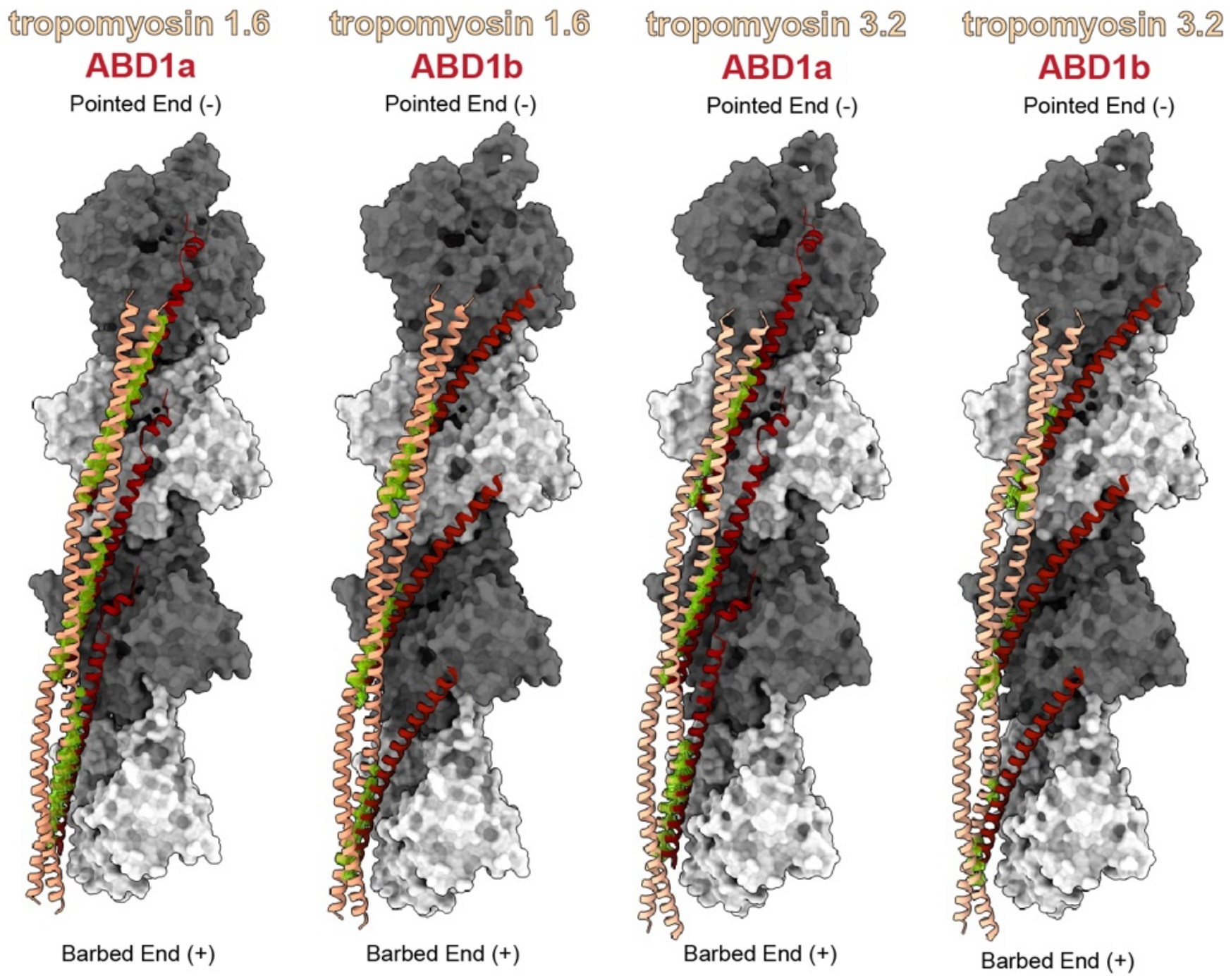

Supplementary Fig. 19. Drebrin sterically competes with non-muscle tropomyosin isoforms. Models of F-actin-bound non-muscle tropomyosin Tpm1.6 and non-muscle tropomyosin 3.2 (apo-states, PDBs: 7ZTC and 7ZTD respectively[16]), are superimposed onto our drebrin ABD-bound F-actin models (actin portion as the reference), and resultant clashes (detected with default settings in ChimeraX[3]) shown as green density.

Supplementary Table 1. Cryo-EM data collection, refinement and validation statistics

	Drebrin-E[135-355] ABD1a F-actin (EMD-55893, PDB: 9TG8)	Drebrin-E[135-355] ABD1b F-actin (EMD-55894, PDB: 9TG9)	Drebrin-E[135-355] ABD2 F-actin (EMD-55895, PDB: 9TGA)
**Data collection and processing**			
Magnification (actual)	150,376	150,376	150,376
Voltage (kV)	300kv	300kv	300kv
Electron exposure (e^-/Å^2)	45	45	45
Defocus range (μm)	-0.7 to -2.5μm	-0.7 to -2.5μm	-0.7 to -2.5μm
Pixel size (Å)	0.931	0.931	0.931
Symmetry imposed	C1	C1	C1
Initial micrographs (#)	3243	3243	3243
Final single particles used (#)	102,155	115,898	149,259
Map resolution (Å)	2.37	2.34	2.29
FSC threshold	0.143	0.143	0.143
Map resolution range (Å)	2.36-3.33	2.30-3.22	2.16-3.00
Measured helical symmetry			
Helical rise (Å)	27.6	27.6	27.6
Helical twist (º)	-166.653	-166.653	-166.653
**Refinement**			
Initial model used	PDB: 8a2s (actin)	PDB: 8a2s (actin)	PDB: 8a2s (actin)
Model resolution (Å)	2.43	2.31	2.23
FSC threshold	0.5	0.5	0.5
Map sharpening B factor (Å^2)	-32.29	-30.28	-30.87
Model composition			
Non-hydrogen atoms	6701	6534	6432
Protein residues	805	794	770
Waters	265	184	352
Ligands	4	4	4
B factors (Å^2)			
Protein	44.26	43.54	34.26
Waters	41.93	41.36	34.05
Ligand	36.01	33.09	24.78
R.m.s. deviations			
Bond lengths (Å)	0.003	0.004	0.004
Bond angles (°)	0.573	0.965	1.009
Validation			
MolProbity score	1.16	1.00	1.15
Clashscore	3.76	2.22	3.57

Poor rotamers (%)	0.15	0.44	0.15
Ramachandran plot			
Favored (%)	98.99	99.23	99.08
Allowed (%)	1.01	0.77	0.92
Disallowed (%)	0.00	0.00	0.00
Occupancy			
Mean	0.76	0.79	0.77
Occ = 1 (%)	1.01	0.40	0.51
0 < Occ < 1(%)	98.96	99.56	99.49
Occ > 1(%)	0.00	0.00	0.00

References

1. Kardos, J. et al. Guide to the structural characterization of protein aggregates and amyloid fibrils by CD spectroscopy. *Protein Sci* **34**, e70066 (2025).
2. Oosterheert, W., Klink, B.U., Belyy, A., Pospich, S. & Raunser, S. Structural basis of actin filament assembly and aging. *Nature* **611**, 374-379 (2022).
3. Meng, E.C. et al. UCSF ChimeraX: Tools for structure building and analysis. *Protein Sci* **32**, e4792 (2023).
4. Punjani, A., Rubinstein, J.L., Fleet, D.J. & Brubaker, M.A. cryoSPARC: algorithms for rapid unsupervised cryo-EM structure determination. *Nat Methods* **14**, 290-296 (2017).
5. Burt, A. et al. An image processing pipeline for electron cryo-tomography in RELION-5. *FEBS Open Bio* **14**, 1788-1804 (2024).
6. Chen, S. et al. High-resolution noise substitution to measure overfitting and validate resolution in 3D structure determination by single particle electron cryomicroscopy. *Ultramicroscopy* **135**, 24-35 (2013).
7. Boiero Sanders, M., Oosterheert, W., Hofnagel, O., Bieling, P. & Raunser, S. Phalloidin and DNase I-bound F-actin pointed end structures reveal principles of filament stabilization and disassembly. *Nat Commun* **15**, 7969 (2024).
8. Abramson, J. et al. Accurate structure prediction of biomolecular interactions with AlphaFold 3. *Nature* **630**, 493-500 (2024).
9. Tordai, H. et al. Analysis of AlphaMissense data in different protein groups and structural context. *Sci Data* **11**, 495 (2024).
10. Drozdetskiy, A., Cole, C., Procter, J. & Barton, G.J. JPred4: a protein secondary structure prediction server. *Nucleic Acids Res* **43**, W389-94 (2015).
11. Varga, S. et al. Dynamic Interchange of Local Residue-Residue Interactions in the Largely Extended Single Alpha-Helix in Drebrin. *Biochem J* **482**, 383-99 (2025).
12. Varga, S., Kaasen, J.M., Gaspari, Z., Peterfia, B.F. & Mulder, F.A.A. Resonance assignment of the intrinsically disordered actin-binding region of Drebrin. *Biomol NMR Assign* (2025).
13. Shen, Y. & Bax, A. Protein backbone and sidechain torsion angles predicted from NMR chemical shifts using artificial neural networks. *J Biomol NMR* **56**, 227-41 (2013).
14. Sanchez-Garcia, R. et al. DeepEMhancer: a deep learning solution for cryo-EM volume post-processing. *Commun Biol* **4**, 874 (2021).
15. Stirling, D.R. et al. CellProfiler 4: improvements in speed, utility and usability. *BMC Bioinformatics* **22**, 433 (2021).
16. Selvaraj, M. et al. Structural basis underlying specific biochemical activities of non-muscle tropomyosin isoforms. *Cell Rep* **42**, 111900 (2023).
