# Supplementary material for: Structural mechanisms of drebrin-mediated F-actin network modulation
Source: Nat Commun. 2026 Jun 23;17:7894. doi: 10.1038/s41467-026-74543-6 (PMC13443164; doi:10.1038/s41467-026-74543-6)
Supplement: Supplementary file 3 [file 41467_2026_74543_MOESM3_ESM.pdf]

nature portfolio

Peer Review File

Structural mechanisms of drebrin-mediated F-actin network modulation

Corresponding Author: Dr Joseph Atherton

This manuscript has been previously reviewed at another journal. This document only contains information relating to versions considered at Nature Communications.

This file contains all reviewer reports in order by version, followed by all author rebuttals in order by version.

Version 0:

Reviewer comments:

Reviewer #1

(Remarks to the Author)
I co-reviewed this manuscript with one of the reviewers who provided the listed reports. This is part of the Nature Communications initiative to facilitate training in peer review and to provide appropriate recognition for Early Career Researchers who co-review manuscripts.

Reviewer #2

(Remarks to the Author)

This paper by Zhao et al. represents a solid and important contribution. Using primarily cryo-EM, the authors elucidate how drebrin interacts with F-actin. Drebrin is an actin-binding protein with well-documented roles in regulating actin filament organization and dynamics, particularly in dendritic spines. The work is technically sound, and the cryo-EM structures are of excellent quality and relatively high resolution.

The main findings can be summarized as follows:
(1) Drebrin contains two actin-binding domains, ABD1 and ABD2, with ABD1 adopting two distinct binding conformations on F-actin.
(2) The two binding domains, whose structures were determined independently using non-overlapping drebrin constructs, each contact two actin protomers on F-actin. Their binding surfaces partially overlap and therefore are expected to interact with different actin protomers along the filament.
(3) The combined drebrin binding footprint on F-actin partially overlaps with those of several other actin-binding proteins, predicting competitive binding to the filament.
(4) Despite containing two actin-binding domains, drebrin does not appear to bundle actin filaments in vitro; nevertheless, it induces filopodia in COS7 cells, and intriguingly these filopodia frequently contain a microtubule running along their length, a feature not typically observed in conventional filopodia.

Based on the technical quality of the work and these novel observations, I support publication of this paper in Nature Communications.

Major criticism

1. My main concern is that the results presented here could likely be conveyed effectively in two to three figures and described in a much shorter text. Instead, the paper is excessively long, with seven main figures and ten supplementary figures, several of which appear repetitive, trivial, or both. The writing is often overly technical and not particularly accessible to the general Nature Communications readership. Overall, the manuscript gives the impression of having been artificially inflated to create a longer story, whereas a more focused and concise presentation would be far more effective and scientifically compelling. Several examples illustrate this issue. The D-loop analysis in Fig. 5, for instance, could easily be

incorporated into Fig. 1, while the helical rise and twist analysis would be more appropriate for the Supplementary Information. Figures 2 and 3 convey largely overlapping information and could be merged without loss of clarity. Similarly, the model presented in Fig. 7 could be combined with Fig. 6, which illustrates the various protein–protein competitions inferred from structural superpositions. These analyses of competition are predictable and seem rather trivial for a paper in Nature Communications, and they do not warrant the extensive presentation they receive.

2. Another concern relates to the interpretation of the two binding conformations of ABD1. Could these conformations arise artificially from competition on oversaturated F-actin? Just as the authors conclude that ABD1 and ABD2 interfere with each other's binding on the filament, preventing their simultaneous structural analysis, it is plausible that the shifted helix α2 observed in the ABD1b conformation, which allows the C-terminus of another ABD1 to bind, is also a consequence of such collusion. No functional significance is assigned to the two ABD1 conformations, and in my view this technical explanation is likely the true origin of the observed heterogeneity. This point must be addressed explicitly. Moreover, the exact ratio of ABD1a versus ABD1b should be reported. Were different ABD1 concentrations tested, and if so, did they yield similar ABD1a/ABD1b ratios?

Minor comments

1. In the cartoon in Fig. 1a, ABD1 and ABD2 are depicted as two separate segments, presumably because each consists of two helices. Without clear numbering and labeling, this nuance may be more confusing than helpful. It may also be useful to include drebrin-A for comparison and to highlight a conserved F- actin-binding mechanism. The legend should explicitly describe the domains shown in Fig. 1a.

2. Several panels in Fig. 1 (c, d, and h) display actin side chains, but these are not labeled, and it is unclear why they are shown.

3. It would be interesting to discuss why drebrin's ADFH domain does not bind actin, unlike other members of this protein family.

4. Referring to cofilin as a "depolymerase" (Introduction, fifth paragraph) is inaccurate. While cofilin promotes filament disassembly, it does so primarily through severing; depolymerization occurs largely in conjunction with CAP.

5. The Discussion could speculate on how drebrin overexpression induces filopodia in COS7 cells despite the apparent lack of actin-bundling activity.

6. The sentence "no significant drebrin-induced remodelling of F-actin is observed" at the end of the Introduction is potentially confusing. "Actin remodeling" is often interpreted in a cellular context; here it appears to refer specifically to the absence of major structural changes in F-actin and should be reworded.

7. In Fig. 6e, tropomyosin is shown in a single conformation, whereas it can adopt three different positions on F-actin. This should be explained more clearly, although in my opinion this entire figure is either unnecessary or better placed in the Supplementary Information. Also, was competition with cofilin tested in the context of the cofilactin twisted filament?

8. The statement "Considering drebrin's F-actin binding domains are separated by a flexible L3 with a maximum stretched length of ~15 nm, it seems unlikely drebrin is the core F-actin cross-linker organising these tight bundles" (Results, page 6) would benefit from quantitative support. What is the measured distance between filaments in the filopodia-like structures formed in COS7 cells upon drebrin-E–eYFP expression? I also wonder whether the lack of bundling observed in vitro could result from oversaturation of binding sites. Were lower drebrin concentrations tested?

9. The sentence "Interestingly, in half the drebrin-E–eYFP-transfected filopodia imaged we observed two or more microtubules..." (Results, page 6) highlights what I consider one of the most intriguing findings of this study. Unfortunately, this observation is neither pursued nor explained and represents a missed opportunity to expand the biological significance of the work.

10. HowwerefilopodiaquantifiedinExtendedDataFig.7f?Howwerefilopodiadistinguishedfrom microspikes? Are these bona fide, myosin-10–positive filopodia, or simply membrane projections?
11. RegardingthediscussionofdrebrinbindingtoF-versusG-actin,didtheauthorsexperimentallytest whether ABD2 binds G-actin? Based on the structure it seems plausible. If this was not tested, the authors should avoid overinterpretation.

Reviewer #3

(Remarks to the Author)
NCOMMS-25-102218-T: Structural mechanisms of drebrin-mediated F-actin network modulation

In the manuscript by Zhao et al., the authors structurally investigate the interaction of drebrinE with F-actin using a combination of cryo-EM, cryo-ET, NMR spectroscopy, and Al-phaFold 3-based structural prediction. Filamentous actin (F-actin) is a central regulator of subcellular organization in many cell types and plays a particularly important role in neu-ronal growth and intracellular transport from the soma to synapses. drebrinE is predomi-nantly expressed during early neuronal development, whereas in mature neurons the splice variant drebrin-A is more abundant.

drebrinE is known to be an important modulator of multiple F-actin-binding proteins, in-cluding the actin depolymerase cofilin, the bundling proteins fascin and α-actinin, as well as tropomyosin and myosins. These regulatory effects have been demonstrated by several research groups using in vitro assays with either embryonic rat brain-derived drebrinE or full-length recombinant protein expressed in E. coli. The present study now provides a structural framework that helps explain how drebrinE modulates the binding of these F-actin-associated proteins. The findings are well aligned with recent cryo-EM studies inves-tigating F-actin interactions with coronin, cofilin, AIP1(https://doi.org/10.1016/j.cell.2025.09.016) , fascin (https://doi.org/10.1038/s41594-024-01477-2), and tropomyosin (https://doi.org/10.1038/nature14033), thereby placing the work in a broader and timely structural context.

Major Part I: AlphaFold3 and data interpretation:

A key strength of the study is the thoughtful integration of AlphaFold 3 (AF3) with cryo-EM data to validate formation of the F-actin-drebrin-E135-355 complex. As I understand the workflow, the authors first generated AF3 models using six F-actin protomers and two drebrinE 135-355 proteins and then used these predictions to guide construct design and structural interpretation.
1) Running AF3 with 6 actin and 1 drebrinE-1-431 reveals a structural interaction model of the f-actin drebrin complex including the ADFH domain, which helps the reader to understand the construct design process (new AF3 model: Figure 1a). The authors never stated, why did not created an AF3 model of the drebrinE-1-431?
2) drebrinE-135-355: Removal of the ADFH domain (residues 1-134), which would otherwise contribute additional density (~15 kDa) and potentially complicate cryo-EM refinement and is explainable with new AF3 model.
3) drebrinE-135-355: The new AF3 model (Figure 1a) predicts an additional α-helix ex-tending from approximately V348 to T360 (P347-S360). Truncation at residue 355 re-moves several acidic residues (D/E), which may destabilize this helix and result in in-creased flexibility of the C-terminal region.
4) In Extended Data Fig. 1c the alpha-helical content of AF3 is not agreeing with the secondary elements show the AF3 model of Extended Data Fig. 1f. In the AF3 model the L3 shows no-alpha-helical content, but in Extended Data Fig. 1c AF3 secondary structure prediction contains alpha-helical content similar to JPRED4, which shows a continuous alpha-helical content between L3 and ABD2.

Taken together, it would be helpful to rephrase the description of construct design of drebrin-E135-355, drebrinE-135-255, and drebrinE-256-355 to place less emphasis on sequence-based secondary-structure prediction and more emphasis on the new AF3 complex model. In addition, giving the AF3 model of the f-actin-drebrinE1-431 complex a more prominent position in the manuscript (rather than limiting it to Extended Data Fig. 1f)

Major Part II: Cryo-EM evaluation:

Initially, the cryo-EM data for the F-actin-drebrinE 1-431 and F-actin-drebrinE 135-355 com-plexes were processed, as shown in Extended Data Fig. 1d and e. The cryo-EM density of drebrinE 1-431 displays more continuous and better-resolved α-helical features than that of drebrinE 135-355 (Fig. 1c,d). The authors decided to proceed with the drebrinE-135-355 dataset based on the absence of detectable binding contributions from the ADFH or PP domains, a choice that can be rationalized by the new AF3 model.

5) The α-helical region of drebrinE 1-431 appears better resolved, which may reflect a transient interaction of the ADFH domain with F-actin that stabilizes a single ABD1a conformation. In this context, it would be informative to also perform focused classification on the drebrinE 1-431 dataset.
6) In contrast, the α-helical density of drebrinE 135-355 shows breaks or weakened regions, consistent with the presence of two conformations (ABD1a and ABD1b).

The subsequent cryo-EM data processing using focused classification is well executed and described in sufficient detail to allow the reader to follow the workflow. The authors first re-solve ABD1a and ABD1b maps from the F-actin-drebrinE-135-255 dataset and subsequently apply the same strategy to the F-actin-drebrinE 256-355 dataset, yielding a reconstruction corresponding to ABD2.
7) Please report the FSC@0.143 resolution for the focused-classification reconstructions shown in Extended Data Fig. 2a to better quantify map quality.
8) if feasible, it would be informative to use model_angelo to build atomic models for ABD1a and ABD1b from the drebrinE 135-255 dataset and for ABD2 from the drebrinE 256-355 dataset. For ABD2, the presence of clear side-chain densities suggests that de novo atomic model building should be possible.
9) A comparison of these newly built models with the existing refined structures (ABD1a, ABD1b, and ABD2) would help assess whether any structural differences exists based on the constructs.

The authors then use the local information obtained for ABD1a/b (135-255) and ABD2 (256-355) to reprocess the f-actin-DrebrinE-135-355 dataset and determine high-resolution structures of ABD1a/b (135-355) and ABD2 (135-355).
10) Please consider adding overlay figures of the cryo-EM maps and corresponding atomic models for ABD1a and ABD1b. At present, the manuscript mainly presents side-by-side views (Fig. 1b-d, Fig. 2a,b,d,e, and Fig. 3a,b), which makes it difficult for the reader to directly appreciate subtle structural differences.
11) In Extended Data Fig. 4a-c, please include the phase-randomized FSC@0.143 curves.
12) In addition, for Extended Data Fig. 4a and b, there is a pronounced dip in the FSC@0.143 curve at ~0.1 $Å^{-1}$ (corresponding to ~10 Å). In amyloid fibril reconstructions (typically around 4.7 Å), such features can sometimes arise from imperfect half-map alignment prior to post-processing.

The authors further discuss possible binding sites and positional relationships of ABD1a, ABD1b, and ABD2 on F-actin based on the shifted-particle cryo-EM reconstructions ap-proach and propose schematic models illustrated in Fig. 1f and Fig. 3c. While the conceptual approach is compelling, additional quantitative analyses based on _rlnHelicalTubeIDs could further strengthen the conclusions, assuming ABD1a, ABD1b, and ABD2 originate from the same filament-picking job.
13) As a first step, reporting how many particles of each class (ABD1a, ABD1b, ABD2) populate individual _rlnHelicalTubeIDs would be informative. A histogram of particle counts per HelicalTubeIDs could help define a meaningful cutoff (e.g., filaments containing at least seven particles). Using this filtered set, minimal distances between adjacent particles of each class (using the refined particles position for ABD1a, ABD1b, ABD2) along a filament could then be calculated and plotted as distance dis-tributions.
14) In a second step, analyzing class combinations within individual filaments (e.g. filaments containing ABD1a/ABD2, ABD1b/ABD2, or ABD1a/ABD1b/ABD2) could provide additional insight into binding-site preferences and occupancy patterns. Measuring inter-particle distances within these combinations may reveal spatial correlations that further support the proposed schematic models.

Major Part III: NMR interpretation:

The NMR experiments were conducted using drebrinE 135-355 in the apo state and in the absence of F-actin, as also stated by the authors.
15) Based on the BMRB entries, the N-terminal region of the sequence in BMRB 52895 overlaps with the C-terminal region of BMRB 52729. It would be helpful if the authors could indicate in the 15N-HSQC spectrum which residues originate from which assignment.
16) Typically, CheZOD Z-scores of ≥8 are interpreted as indicative of well-ordered regions, values ≥3 as partially ordered, and values <3 as disordered. In Extended Data Fig. 8, most residues show Z-scores distributed around ~2, while residues in the L3 region cluster around ~0.5. Given these values, it is not clear how the apo-state NMR data support structural agreement with the cryo-EM observations.

Minor I:
To date, drebrin has been demonstrated to compete with F-actin depolymerase cofilin, F-actin-bundlers fascin and α-actinin, tropomyosin and myosins, suppressing their activities 55,59-62.
17) Please add the cofilin citation.

Minor II: Structural competition between drebrinE and fascin, cofilin, α-actinin, tropomyosin and myosins.
18) Most of the work cited by the authors for the competition assay of full-length drebrinE used extracts from embryonic rat brains (some E. coli-expressed) and studied the interaction via spin-down assays. Could the floppy tail of ~346 residues and the ADHF domain also influence binding?

Reviewer #4

(Remarks to the Author)
Drebrin is one of the key regulators of actin cytoskeleton in neuronal and non-neuronal cells. High resolution structural information on drebrin complexes with the interacting partners could provide valuable new insights on some of its documented cellular activities and biochemical properties. Filamentous actin is recognized as a main drebrin binding factor, thus, this research direction is important and timely. Structural work on F-actin-drebrin complex is complicated by the binding stoichiometry (drebrin binds to multiple actin protomers). This work presents cryoEM reconstruction of two drebrin fragments with actin filaments, combined with the modeling of their relative positions on actin lattice. This resulted in identification of 6 different modes of binding with each class representing 14-33% of total particles. Based on the structure presented, drebrin-bound F-actin closely resemble ADP-Pi state ("young filaments"), which is interesting and novel. Another interesting observation made is that drebrin binding interface is a better match for actin subunits within a polymer (flattened) as opposed to G-actin. This provides a structural explanation for drebrin's binding preference to actin polymer vs monomers, which is novel and valuable to the field. My main concerns regarding this study include insufficient description of drebrin-actin interface, lack of in vitro validation of the structure, as well as clarity of presentation/writing (detailed below).
Major issues (scientific):
The description of the F-actin-drebrin interface is lacking. The authors identify the drebrin residues involved in its interaction with actin but the actin's side of the interface is unclear as well as the chemical nature of the bonds stabilizing it (salt bridges, H-bonds, hydrophobic interactions etc). A complete list of actin and drebrin residues involved in the interaction and the types of bonding should accompany the description of different binding modes. Moreover, key molecular details of the interface (including individual side chains) should be shown in the main figures. Comparison of the structure with the Alphafold-generated model of the F-actin-drebrin complex (Extended Fig. 1) would be also helpful. The related text needs to be revised.
Validation of the structure would be important especially since there are multiple modes of binding. I disagree with the use of filopodia formation in COS7 cells as a readout of drebrin binding (Extended Data Fig. 7d-l). Filopodia formation is a complex process, and drebrin was shown to influence multiple proteins potentially involved in this process (fascin, Arp2/3, formins). Thus, in my opinion, alterations of the filopodia phenotype may not necessarily report on inhibition of drebrin-F-actin interaction. Microscopy or biochemical assays comparing a WT drebrin construct with the site-specific mutants designed to weaken the interface would strengthen the paper. I do find the Extended Data Fig. 7d-l interesting, especially the results with the R221A mutant However, in my opinion, it would be important to compliment this cellular work with the in vitro assays.
General comments:

Introduction provides an extensive survey of drebrin functions and activities. However, the available low resolution structural information on drebrin-F-actin complex is not mentioned (Ref 2). Authors should revise the Introduction to include the available structural information including the previously reported multiple binding modes.
ADP-Pi-bound-like conformation in the drebrin decorated F-actin is interesting and novel.
Incompatibility of drebrin interface with G-actin (as opposed to F-actin) is interesting and important.
I'd like to bring to the authors' attention an issue of the binding stoichiometry which is ~1:5 (full length drebrin : actin) based on data from multiple groups. Citing Ref 55 in context of the 1:3 (drebrin:actin) binding stoichiometry is not factual (the stoichiometry 1:5 was reported in that study for the endogenous drebrin E). This means that a part of F-actin-drebrin interaction interface is still unknown. The authors should discuss how this affects their main conclusions.
My understanding is that it is accepted that drebrin is not a strong actin bundling protein. However, it was suggested that drebrin's bundling activity is regulated in the phosphorylation-dependent manner. With the new structural information obtained, the authors should discuss the drebrin phosphorylation pattern in the context of their cryoEM structure. Are any of the phospho-regulated residues directly involved in actin-drebrin interaction? If so, will phosphorylation affect this interaction, potentially allowing only a subset of the possible binding states?
Based on the biochemical data, drebrin has stronger effect on the barbed end of F-actin (compared to the pointed end). The model proposed in Fig. 7 is not consistent with biochemical data which needs to be discussed.
Fig. 3e - % occupancy for all the classes combined does not add up to 100%.
Fig. 4j (microtubule entry). Missing control. Although filopodia will be much less common in the cells transfected with eYFP, the authors should present the results of the data analysis with eYFP alone. The frequency of the microtubule entry is not meaningful without a reference point.
One of my main points of criticism is that the paper (in my opinion) could be written more clearly. Many figure panels appear to be redundant, unnecessary or difficult to understand. Colored letters layered onto the structures obscure the structural features.
This manuscript is very difficult to follow. It needs to be restructured and edited for clarity to be accessible to the general audience. Some of the examples (among many others) are below:
P.3 Unclear. "Picked particles corresponding to decorated segments of F-actin filaments were subjected to image processing including focused classification such as to identify different conformations of drebrin-E135-256 or drebrin-E256-355 if present"
P.9 Unclear. "Furthermore, even within an F-actin protomer pair bound by an ABD, binding of a particular ABP can be sterically permitted only to one of the two protomers."

Version 1:

Reviewer comments:

Reviewer #1

(Remarks to the Author)
I co-reviewed this manuscript with one of the reviewers who provided the listed reports. This is part of the Nature Communications initiative to facilitate training in peer review and to provide appropriate recognition for Early Career Researchers who co-review manuscripts.

Reviewer #2

(Remarks to the Author)
I think the authors' responses and the changes made to the manuscript and figures are appropriate, and I have no further comments or concerns regarding publication of this paper in Nature Communications.

Reviewer #3

(Remarks to the Author)
Dear Editor,

Thank you for sharing the authors' response. I have only two minor points that I would ask the authors to consider before publication.

First, regarding the response to points 8 and 9, I appreciate that the authors tested ModelAngelo model building and compared the single domain datasets with the deposited ABD1 and ABD2 models. However, I would still suggest modifying the figure presentation. In my view, it would be clearer to show all densities in the postprocessed form, since this corresponds to the input format used for ModelAngelo, and then fit the atomic model into the density while displaying the residues explicitly rather than using a cartoon representation. This would make the quality of the density and the model fit easier to assess. I agree that the comparison between the newly built or fitted models and the existing refined structures, ABD1a, ABD1b, and ABD2, is useful.

For the NMR section (16), I remain concerned that the CheZOD analysis may be misleading for readers if the authors did not use the full set of chemical shifts normally required for a meaningful CheZOD calculation. The authors themselves indicate that the relatively low scores likely arise from using only amide nitrogen and proton assignments rather than a full backbone

assignment. I would therefore suggest that the CheZOD analysis be removed, and that the authors instead explicitly state that the alpha-helical section of drebrin was already estimated by Varga et al.

Overall, these are minor points, but I believe addressing them would improve clarity and avoid potential overinterpretation of the density fitting and NMR analyses.

Reviewer #4

(Remarks to the Author)
This manuscript was extensively revised, and the data presentation was significantly improved. A few important points need to be addressed before the publication:
(1) Regarding the validation of the structural work, the biochemical data presented in Supplementary Fig. 11 is convincing. However, a subset of mutants tested in biochemical experiments (283A/286A and 180S/181S) is different from that tested in COS7 cells. Therefore, the statement "We further validated the drebrin-F-actin interface…" (line 199-200) should be removed and replaced with "We tested the functional importance of each ABD1 conformation for filopodial induction…" (line 199-200, P4) to make it factual. The presented cellular work has its own independent value but as the authors recognize in their response, filopodia formation is a complex process and its induction cannot serve as a direct validation of a structure. Thus, only the data shown in Supplemental Figure 11 is connected to such validation.
(2) It should be clear from the text of the Discussion that this work was done with the truncated drebrin constructs. Thus, the authors should not use a term "drebrin" while describing their data with such constructs (examples include lines 317, 324, 329, 380, 410, and 424). Instead, they should indicate a construct utilized for a specific experiment (such as drebrin135-355 for the bundling data in Fig. 3h (line 380) etc).

Open Access This Peer Review File is licensed under a Creative Commons Attribution 4.0 International License, which permits use, sharing, adaptation, distribution and reproduction in any medium or format, as long as you give appropriate credit to the original author(s) and the source, provide a link to the Creative Commons license, and indicate if changes were made.
In cases where reviewers are anonymous, credit should be given to 'Anonymous Referee' and the source.
The images or other third party material in this Peer Review File are included in the article's Creative Commons license, unless indicated otherwise in a credit line to the material. If material is not included in the article's Creative Commons license and your intended use is not permitted by statutory regulation or exceeds the permitted use, you will need to obtain permission directly from the copyright holder.
To view a copy of this license, visit https://creativecommons.org/licenses/by/4.0/

Reviewer #2 (remarks to the author):

This paper by Zhao et al. represents a solid and important contribution. Using primarily cryo-EM, the authors elucidate how drebrin interacts with F-actin. Drebrin is an actin-binding protein with well-documented roles in regulating actin filament organization and dynamics, particularly in dendritic spines. The work is technically sound, and the cryo-EM structures are of excellent quality and relatively high resolution.

The main findings can be summarized as follows:
(1) Drebrin contains two actin-binding domains, ABD1 and ABD2, with ABD1 adopting two distinct binding conformations on F-actin.
(2) The two binding domains, whose structures were determined independently using non-overlapping drebrin constructs, each contact two actin protomers on F-actin. Their binding surfaces partially overlap and therefore are expected to interact with different actin protomers along the filament.
(3) The combined drebrin binding footprint on F-actin partially overlaps with those of several other actin-binding proteins, predicting competitive binding to the filament.
(4) Despite containing two actin-binding domains, drebrin does not appear to bundle actin filaments in vitro; nevertheless, it induces filopodia in COS7 cells, and intriguingly these filopodia frequently contain a microtubule running along their length, a feature not typically observed in conventional filopodia.

Based on the technical quality of the work and these novel observations, I support publication of this paper in Nature Communications.

We thank the reviewer for their positive evaluation of the technical quality and scientific importance of our work.

Major criticism

1. My main concern is that the results presented here could likely be conveyed effectively in two to three figures and described in a much shorter text. Instead, the paper is excessively long, with seven main figures and ten supplementary figures, several of which appear repetitive, trivial, or both. The writing is often overly technical and not particularly accessible to the general Nature Communications readership. Overall, the manuscript gives the impression of having been artificially inflated to create a longer story, whereas a more focused and concise presentation would be far more effective and scientifically compelling. Several examples illustrate this issue. The D-loop analysis in Fig. 5, for instance, could easily be incorporated into Fig. 1, while the helical rise and twist analysis would be more appropriate for the Supplementary Information. Figures 2 and 3 convey largely overlapping information and could be merged without loss of clarity. Similarly, the model presented in Fig. 7 could be combined with Fig. 6, which illustrates the various protein–protein competitions inferred from structural superpositions. These analyses of competition are predictable and seem rather trivial for a paper in Nature Communications, and they do not warrant the extensive presentation they receive.

When writing the manuscript, we attempted to identify and address the key questions in the field (see introduction) regarding drebrin structure and function in our text and figures. In revising the manuscript, we have considered all reviewers, with some reviewer comments asking for some additional or expanded analyses. Therefore, our revised manuscript represents a balance between these requests and an effort to make it more concise;

1) We undertook a major rewrite and reordering of the results section to make them more concise, less deeply descriptive and more easily interpretable to a broad set of readers.

2) Despite including more analyses in response to reviewer comments, we have shortened the main text (Introduction, Results and Discussion) by over ~600 words to 4248 words, which is now well under the Nature Communications suggested limit of 5000 words.

3) We have now reduced the number of main figures to 5 (instead of 7, with Nature Communications having a 10 main figure limit) and moved much data, which we feel are still important results and central to the narrative, to supplementary figures.

2. Another concern relates to the interpretation of the two binding conformations of ABD1. Could these conformations arise artificially from competition on oversaturated F-actin? Just as the authors conclude that ABD1 and ABD2 interfere with each other's binding on the filament, preventing their simultaneous structural analysis, it is plausible that the shifted helix α2 observed in the ABD1b conformation, which allows the C-terminus of another ABD1 to bind, is also a consequence of such collusion. No functional significance is assigned to the two ABD1 conformations, and in my view this technical explanation is likely the true origin of the observed heterogeneity. This point must be addressed explicitly. Moreover, the exact ratio of ABD1a versus ABD1b should be reported. Were different ABD1 concentrations tested, and if so, did they yield similar ABD1a/ABD1b ratios?

We also considered the possibility that the ABD1b conformation could be artificially induced to avoid steric clashes due to high occupancy of drebrin ABD1 on the filament. We believe this not to be the case because;

1) Several conserved residues in ABD1 were found to only interact with F-actin in the ABD1b conformation, suggesting the ABD1b conformation is functional physiologically (Figure 2).
2) Mutations to conserved residues that interact with F-actin in either only the ABD1a or ABDb conformation had physiological effects in a cellular filopodia induction assay (Supplementary Figure 12). A mutation that was designed to impact both conformations had the strongest effect.
3) Classification revealed that 61% of ABD1b does not adopt this conformation in order avoid steric clashes with another neighbouring ABD1 towards the pointed end (Classes 3 & 4 in Supplementary Figure 16c).
4) Our F-actin preparations for cryo-EM do not appear to be saturated by drebrin as a number of ABD binding sites on drebrin are unoccupied after classification (Supplementary Figures 3,4 and 16).

We have now explicitly presented point 3) in the description of Supplementary Figure 16.

Minor comments

1. In the cartoon in Fig. 1a, ABD1 and ABD2 are depicted as two separate segments, presumably because each consists of two helices. Without clear numbering and labeling, this nuance may be more confusing than helpful. It may also be useful to include drebrin-A for comparison and to highlight a conserved F- actin-binding mechanism. The legend should explicitly describe the domains shown in Fig. 1a.

We appreciate this suggestion and have modified the domain schematic, now Supplementary Figure 1a to;
1) Remove the secondary structure representations from ABD1 and ABD2.
2) Add drebrin-A for comparison.

We've also removed secondary structure representations from ABD1 and ABD2 in all domain schematics and other supplementary figure panels.

2. Several panels in Fig. 1 (c, d, and h) display actin side chains, but these are not labelled, and it is unclear why they are shown.

The side chains have been hidden as suggested.

3. It would be interesting to discuss why drebrin's ADFH domain does not bind actin, unlike other members of this protein family.

We agree this is an interesting subject. While the overall fold of ADFH domains are similar between the different ADFH protein family members, the sequences are divergent in key actin-binding regions identified in actin-binding members (PMID: 21850706; PMID: 24836399; PMID: 19768801). However, we felt properly addressing this subject would require an in-depth sequence/structure analysis which is outside of the scope of the manuscript, particularly in the context of trying to keep it concise.

4. Referring to cofilin as a "depolymerase" (Introduction, fifth paragraph) is inaccurate. While cofilin promotes filament disassembly, it does so primarily through severing; depolymerization occurs largely in conjunction with CAP.

We agree the cofilin does not actively depolymerise F-actin but instead promotes its severing in conjunction with other proteins. We have reworded from 'depolymerase' to 'disassembly-promoting factor' when describing cofilin.

5. The Discussion could speculate on how drebrin overexpression induces filopodia in COS7 cells despite the apparent lack of actin-bundling activity.

We have now performed low-speed co-sedimentation assays, which suggest that drebrin's two ABDs linked by a 43 amino acid long flexible region can loosely bundle F-actin in a manner not visualisable by cryo-EM.

We have added further discussion regarding this matter and about how drebrin expression might induce filopodia to the fifth discussion paragraph. While we would like to discuss this in more depth, this is outside of the scope of this manuscript, particularly given our attempts to condense it.

6. The sentence “no significant drebrin-induced remodelling of F-actin is observed” at the end of the Introduction is potentially confusing. “Actin remodelling” is often interpreted in a cellular context; here it appears to refer specifically to the absence of major structural changes in F-actin and should be reworded

We have reworded from ‘remodelling’ to ‘conformational changes’ to refer more to structure than cellular architecture.

7. In Fig. 6e, tropomyosin is shown in a single conformation, whereas it can adopt three different positions on F-actin. This should be explained more clearly, although in my opinion this entire figure is either unnecessary or better placed in the Supplementary Information. Also, was competition with cofilin tested in the context of the cofilactin twisted filament?

Drebrin’s competitive displacements of a number of actin-binding proteins, such as cofilin and myosin, are important to its cellular functions, for example, in neuronal growth cones and synapses (PMID: 34685706; PMID: 19222710; PMID: 38271699). Therefore, we feel it is important to present the mechanisms behind such competitive displacement as a main figure.

In our structural superimpositions of drebrin and tropomyosin, we utilised a structure of a tropomyosin isoform (tpm1.1) abundant in smooth and striated muscles in the absence of accessory proteins, therefore representing an apo-state (PDB: 5JLF). The ‘blocked’, ‘closed’ and ‘open’ states are dependent on complex formation and conformational regulation by troponin (and calcium) and myosin. However, tropomyosin, troponin, myosin are not found in the sarcomeric Z-disc (PMID: 33765442; PMID: 19830582), where drebrin is localised in striated muscle (PMID: 34502296). Myosin and drebrin themselves compete for F-actin binding. Therefore, we did not consider blocked, open and closed states of tropomyosin in this study. We have made clear in the text and figure legends what states and isoforms we are referring to. We have also added comparisons with two further available structures of non-muscle tropomyosins in the apo-state, found on actin in non-muscle tissues such as neuronal growth cones and synapses where drebrin is a regulatory protein (Supplementary Figure 19).

As mentioned briefly at the end of the results, the twisted F-actin induced by cofilin is not compatible with drebrin’s binding with a non-twisted filament. As we discussed, F-actin conformational twist may contribute to the competition of the two proteins for F-actin association (PMID: 25047716). For the structural superimpositions shown, we have now instead superimposed ABD1 and ABD2-bound F-actin to the structure of F-actin bound to a single cofilin (PDB: 6UBY, PMID: 31900364). This approximates the initial binding of cofilin to its binding site on two actin protomers, where only the bound protomer to the pointed end is twisted by the presence of cofilin. We therefore performed our superimpositions instead via the ‘straight’ cofilin-bound actin protomer towards the barbed end. As mentioned in the

results, this superimposition suggests drebrin would sterically block initial access of cofilin to its binding site.

8. The statement “Considering drebrin’s F-actin binding domains are separated by a flexible L3 with a maximum stretched length of ~15 nm, it seems unlikely drebrin is the core F-actin cross-linker organising these tight bundles” (Results, page 6) would benefit from quantitative support. What is the measured distance between filaments in the filopodia-like structures formed in COS7 cells upon drebrin-E–eYFP expression? I also wonder whether the lack of bundling observed in vitro could result from oversaturation of binding sites. Were lower drebrin concentrations tested?

As suggested, we have measured inter-filament distances between filaments in tomograms of drebrin-E–eYFP expressing COS7 cell filopodia, with the analysis shown in Supplementary Figure 18. As we cannot compare with control COS7 cell filopodia because they are absent when drebrin is not transfected, we have plotted inter-filament distances measured from *in vitro* fascin-bundled F-actin for comparison. We have now also performed follow up low-speed co-sedimentation assays which show that drebrin-$E^{135\text{-}355}$ (which contains both ABDs) does bundle F-actin (Figure 3h), but that this is not visualisable by cryo-EM (Figure 3b-g). Considering the ~43 amino acid length and flexibility of loop3 which links the two ABDs, we interpret this as a proportion of drebrin molecules loosely cross-linking F-actin. It is possible therefore, that in cellular filopodia where tight colinear bundles are formed by other F-actin bundlers such as fascin, that drebrin can act as additional cross-links on these filaments, or replace existing cross-links.

9. The sentence “Interestingly, in half the drebrin-E–eYFP-transfected filopodia imaged we observed two or more microtubules...” (Results, page 6) highlights what I consider one of the most intriguing findings of this study. Unfortunately, this observation is neither pursued nor explained and represents a missed opportunity to expand the biological significance of the work.

We agree it is an intriguing finding that we felt worth reporting, however the mechanism of filopodial microtubule entry/retention regulation is outside of the scope of this study. Several studies have investigated this subject, particularly the contribution of drebrin’s interaction with microtubule end-binding protein EB3 (e.g PMID: 23979715; PMID: 34478582; PMID: 18806788). We did not clearly observe F-actin microtubule cross-links in the tomography data.

10. How were filopodia quantified in Extended Data Fig.7f? How were filopodia distinguished from microspikes? Are these bona fide, myosin-10–positive filopodia, or simply membrane projections?

The differences between microspikes and filopodia are somewhat debatable and indeed these structures are related with interconversion reported between the two (e.g PMID: 12566431). However, a key defining feature is their length, with microspikes being shorter and more embedded in the cell cortex/leading edge. Filopodia are commonly described as being microns, rather than nanometres in length. Therefore, we only quantified/imaged protrusions extending at least 1 µm from the lamellipodium edge and further excluded those originating from concave regions of the membrane (considered retraction fibres) as described in the methods.

Both microspikes and filopodia are positive for Myosin-10, where this myosin plays key roles (e.g PMID: 36598940; PMID: 17954606).

11. Regarding the discussion of drebrin binding to F-versus G-actin, did the authors experimentally test whether ABD2 binds G-actin? Based on the structure it seems plausible. If this was not tested, the authors should avoid overinterpretation.

The reviewer is correct to point out that neither this work nor previous studies have experimentally proven drebrin does not bind to G-actin. Therefore, we have toned down our discussion to state that ‘drebrin is structurally incompatible with G-actin association’ even if there is no evidence if it does or does not bind G-actin.

Reviewer #3 (remarks to the author):

NCOMMS-25-102218-T: Structural mechanisms of drebrin-mediated F-actin network modulation

In the manuscript by Zhao et al., the authors structurally investigate the interaction of drebrinE with F-actin using a combination of cryo-EM, cryo-ET, NMR spectroscopy, and AlphaFold 3-based structural prediction. Filamentous actin (F-actin) is a central regulator of subcellular organization in many cell types and plays a particularly important role in neuronal growth and intracellular transport from the soma to synapses. drebrinE is predominantly expressed during early neuronal development, whereas in mature neurons the splice variant drebrin-A is more abundant. drebrinE is known to be an important modulator of multiple F-actin-binding proteins, including the actin depolymerase cofilin, the bundling proteins fascin and α-actinin, as well as tropomyosin and myosins. These regulatory effects have been demonstrated by several research groups using in vitro assays with either embryonic rat brain-derived drebrinE or full-length recombinant protein expressed in E. coli. The present study now provides a structural framework that helps explain how drebrinE modulates the binding of these F-actin-associated proteins. The findings are well aligned with recent cryo-EM studies investigating F-actin interactions with coronin, cofilin, AIP1(https://doi.org/10.1016/j.cell.2025.09.016) , fascin (https://doi.org/10.1038/s41594-024-01477-2), and tropomyosin (https://doi.org/10.1038/nature14033), thereby placing the work in a broader and timely structural context.

We thank the reviewer for recognising the broader relevance and timeliness of this work.

Major Part I: AlphaFold3 and data interpretation:

A key strength of the study is the thoughtful integration of AlphaFold 3 (AF3) with cryo-EM data to validate formation of the F-actin-drebrin-E135-355 complex. As I understand the workflow, the authors first generated AF3 models using six F-actin protomers and two drebrinE 135-355 proteins and then used these predictions to guide construct design and structural interpretation.

Alphafold3 (AF3) was certainly useful in providing initial models for modelling into cryo-EM densities. However, as discussed, our experimental structural data showed

there was also some errors and omissions made by AF3. The combination of experimental cryo-EM and AF3 both helped us with image processing approaches and modelling and as the reviewer says, AF3 provided some validation of our structures. However, our construct boundaries were designed based on structural predictions and experimental work that predated AF3 (PMID: 23979715, PMID: 18806788). The AF3 predictions were only used to aid interpretation and in generating initial models for model building.

1) Running AF3 with 6 actin and 1 drebrinE-1-431 reveals a structural interaction model of the f-actin drebrin complex including the ADFH domain, which helps the reader to understand the construct design process (new AF3 model: Figure 1a). The authors never stated, why did not created an AF3 model of the drebrinE-1-431?

As mentioned above, we did not design our constructs based on AF3 predictions.

We previously performed (but did not present) AF3 of F-actin with drebrin-E$^{1\text{-}431}$, finding that regions outside of the two ABDs have poor pLDDT scores (prediction of fold is low confidence) and/or PAE scores (prediction of residue position is inaccurate). Therefore, whilst these regions are modelled in proximity to F-actin, the predicted structure and/or interaction is very low confidence. These predictions supported our cryo-EM data showing no interaction of the ADFH or other drebrin-E$^{1\text{-}431}$ regions with F-actin. We originally felt therefore that we should present predictions with the shorter drebrin-E$^{135\text{-}355}$ to avoid overwhelming/distracting the reader with information on drebrin domains that are irrelevant to F-actin binding. However, in retrospect we feel it is important to show the predictions of F-actin with drebrin-E$^{1\text{-}431}$ as they provide additional information and supporting evidence that regions outside the two ADBs do not interact with F-actin (see new Supplementary Fig 9a).

2) drebrinE-135-355: Removal of the ADFH domain (residues 1-134), which would otherwise contribute additional density (~15 kDa) and potentially complicate cryo-EM refinement and is explainable with new AF3 model.

As discussed above, the AF3 prediction of drebrin-E$^{1\text{-}431}$ with F-actin agrees with our cryo-EM results, which show no extra density for an ADFH domain of drebrin-E$^{1\text{-}431}$ (Supplementary Fig. 1c and Supplementary Fig. 9a), suggesting no interaction of the ADFH domain with F-actin. We have now further processed the F-actin with drebrin-E$^{1\text{-}431}$ with focussed classification in a manner similar to F-actin with drebrin-E$^{135\text{-}355}$, more clearly demonstrating how no additional densities are found on F-actin bound by the drebrin-E$^{1\text{-}431}$ construct (Supplementary Fig. 4). This supports the majority of research on the F-actin-drebrin interaction which find the ADFH domain does not bind F-actin. Therefore, we focussed our work mostly on F-actin with the drebrin-E$^{135\text{-}355}$ construct in which additional non-binding regions are removed, which gave the best sample quality, micrograph signal-to-noise and eventual reconstruction resolution and quality.

3) drebrinE-135-355: The new AF3 model (Figure 1a) predicts an additional α-helix ex-tending from approximately V348 to T360 (P347-S360). Truncation at residue 355 re-moves several acidic residues (D/E), which may destabilize this helix and result in in-creased flexibility of the C-terminal region.

This region is downstream of ABD2 at the edge of the PP domain. As mentioned above, while an interface is modelled in the AF3 prediction, the pLDDT and PAE scores are very poor suggesting poor fold prediction confidence and no F-actin interaction prediction confidence (Supplementary Fig. 9a). Additionally, we see no density corresponding to an interaction of this short α-helix with F-actin in our cryo-EM maps of drebrin-E^{1-431} (Supplementary Fig. 4). Various biochemical and cellular studies suggest this region downstream of ABD2 does not contribute to F-actin side-binding in vitro or in cells (e.g PMID: 20347847; PMID: 23979715; PMID: 10585290).

4) In Extended Data Fig. 1c the alpha-helical content of AF3 is not agreeing with the secondary elements show the AF3 model of Extended Data Fig. 1f. In the AF3 model the L3 shows no alpha-helical content, but in Extended Data Fig. 1c AF3 secondary structure prediction contains alpha-helical content similar to JPRED4, which shows a continuous alpha-helical content between L3 and ABD2.

This discrepancy is due to the secondary structure content in the original Extended Data Fig. 1c (now Supplementary Figure 9c) being shown for the AlphaFold model in the absence of F-actin (relating to panel b). We have updated the text referring to this panel and labelling in the new Supplementary Figure 9c to be more clear and have also added in the AF3 prediction with F-actin.

Taken together, it would be helpful to rephrase the description of construct design of drebrin-E135-355, drebrinE-135-255, and drebrinE-256-355 to place less emphasis on sequence-based secondary-structure prediction and more emphasis on the new AF3 complex model.

As mentioned above, we did not design our constructs based on AF3 predictions.

In addition, giving the AF3 model of the f-actin-drebrinE1-431 complex a more prominent position in the manuscript (rather than limiting it to Extended Data Fig. 1f)

The key findings of this work are experimentally determined structures of drebrin bound to F-actin, which we want to present to the reader as soon as possible in Figure 1. We feel that also presenting an AF3 prediction (which predicted some regions of the interface incorrectly and only predicted one conformation of ABD1) in Figure 1 would distract from the experimentally determined structures and potentially confuse the reader as to which is the true structure.

Major Part II: Cryo-EM evaluation:

Initially, the cryo-EM data for the F-actin-drebrinE 1-431 and F-actin-drebrinE 135-355 complexes were processed, as shown in Extended Data Fig. 1d and e. The cryo-EM density of drebrinE 1-431 displays more continuous and better-resolved α-helical features than that of drebrinE 135-355 (Fig. 1c,d). The authors decided to proceed with the drebrinE-135-355 dataset based on the absence of detectable binding contributions from the ADFH or PP domains, a choice that can be rationalized by the new AF3 model.

5) The α-helical region of drebrinE 1-431 appears better resolved, which may reflect a transient interaction of the ADFH domain with F-actin that stabilizes a single

ABD1a conformation. In this context, it would be informative to also perform focused classification on the drebrinE 1-431 dataset.

6) In contrast, the α-helical density of drebrinE 135-355 shows breaks or weakened regions, consistent with the presence of two conformations (ABD1a and ABD1b).

The following is in relation to points 5 and 6. The densities accountable to drebrin before 3D classification were essentially indistinguishable for Drebrin-$E^{1\text{-}431}$ and Drebrin-$E^{135\text{-}355}$, however we acknowledge that this is not particularly clear from the way we presented the original Extended Fig. 1d-e. We originally decided to present comparisons between the densities from the two datasets at identical RMS levels, which does not well account for differences in data quality and drebrin occupancy derived from sample preparation (Drebrin-$E^{135\text{-}355}$ had a higher occupancy in our datasets, reflected as more pronounced drebrin density on F-actin at the same absolute threshold). We have therefore adjusted this figure to compare at similar drebrin density thresholds rather than using absolute RMS levels (now Supplementary Figure 1c and d).

As suggested, we have now also performed 3D classification followed by class 3D refinement for our Drebrin-$E^{1\text{-}431}$ dataset (Supplementary Figure 4), as we previously performed on Drebrin-$E^{135\text{-}355}$. This analysis demonstrates no significant differences between Drebrin-$E^{1\text{-}431}$ and Drebrin-$E^{135\text{-}355}$ datasets; Drebrin-$E^{1\text{-}431}$ has classes for ABD1a, ABD1b and ABD2-bound protomer pairs with no additional densities observed that could account for a bound ADFH domain. Nonetheless, data quality was higher for our presented Drebrin-$E^{135\text{-}355}$ dataset, therefore this data remains central in our presented structural work.

7) Please report the FSC@0.143 resolution for the focused-classification reconstructions shown in Extended Data Fig. 2a to better quantify map quality.

We further processed the ABD1 classes from the drebrin-$E^{135\text{-}256}$ dataset to unbinned reconstructions and have reported their resolutions in the new Supplementary Figure 2a.

8) if feasible, it would be informative to use model_angelo to build atomic models for ABD1a and ABD1b from the drebrinE 135-255 dataset and for ABD2 from the drebrinE 256-355 dataset. For ABD2, the presence of clear side-chain densities suggests that de novo atomic model building should be possible.

9) A comparison of these newly built models with the existing refined structures (ABD1a, ABD1b, and ABD2) would help assess whether any structural differences exist based on the constructs.

The following is in response to points 8 and 9. We have now tested building models with modelAngelo into density for these two datasets. The software did a good job with ABD2 (from drebrin-$E^{256\text{-}355}$ dataset) however the ABD1 density from the drebrin-$E^{135\text{-}256}$ dataset was not of sufficient quality for accurate and comprehensive model construction.

To examine differences between ABDs from our drebrin-$E^{135\text{-}355}$ and single domain datasets, the deposited ABD1 and ABD2 models were fitted into

unsharpened densities derived from drebrin-E$^{135-256}$ drebrin-E$^{256-355}$ datasets respectively (see figure for reviewer below). As shown below, the models fit well, consistent with there being no significant difference in the fold or interaction of ABDs with F-actin in the context of single compared tandem-domain constructs. As we mention in the second paragraph of results, we only present density derived from drebrin-E$^{135-355}$-decorated F-actin in the main figures, as it was of the highest quality and resolution and ABD densities were conformationally indistinguishable from the corresponding densities in the drebrin-E$^{135-256}$ drebrin-E$^{256-355}$ datasets.

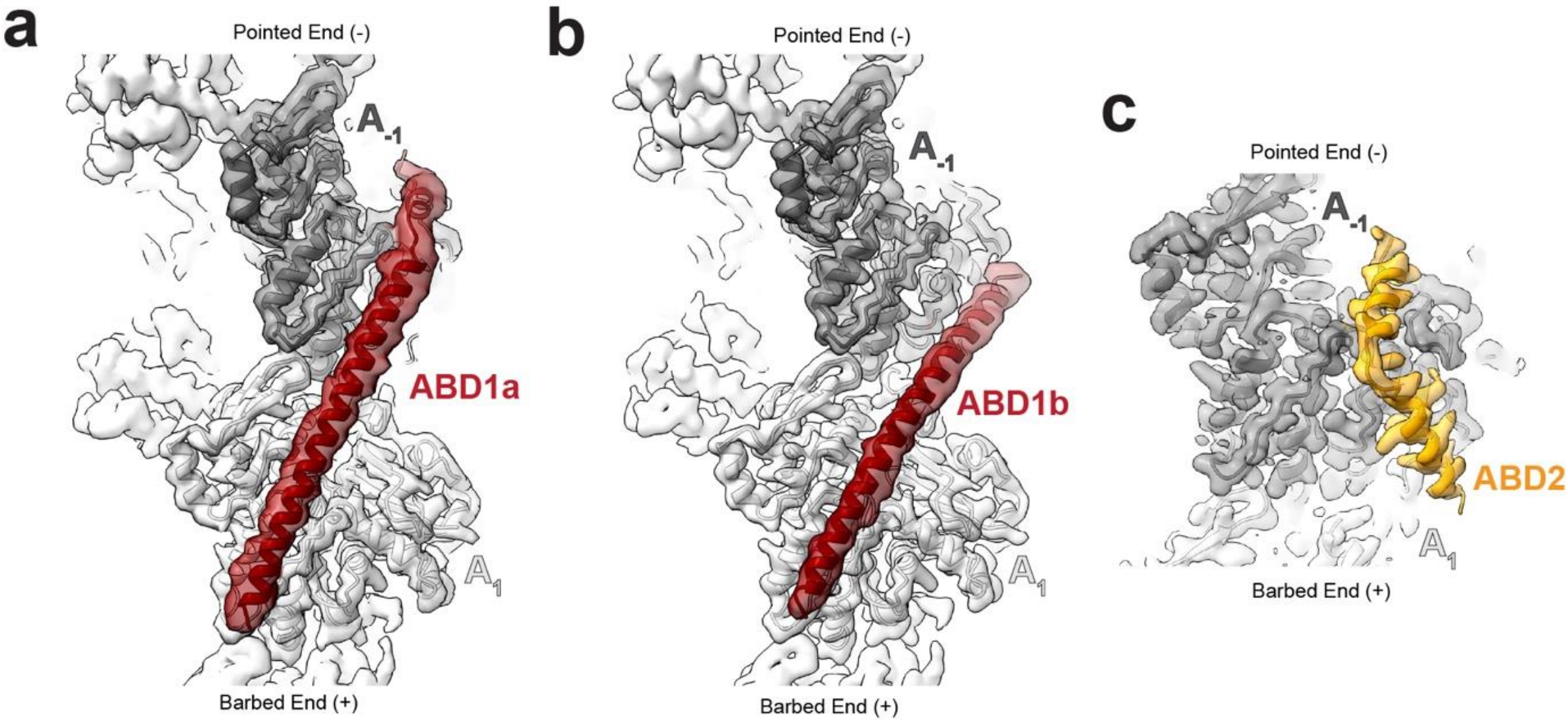

10) Please consider adding overlay figures of the cryo-EM maps and corresponding atomic models for ABD1a and ABD1b. At present, the manuscript mainly presents side-by-side views (Fig. 1b-d, Fig. 2a,b,d,e, and Fig. 3a,b), which makes it difficult for the reader to directly appreciate subtle structural differences.

We have prepared Supplementary Figure 7a which shows overlaid ABD1a and ABD1b to show the conformational deviation. We feel that also adding cryo-EM density would overcomplicate the figure panel, so show models only.

11) In Extended Data Fig. 4a-c, please include the phase-randomized FSC@0.143 curves.

The new Supplementary Figure 5 includes more FSC information, including the phase-randomised FSCs.

12) In addition, for Extended Data Fig. 4a and b, there is a pronounced dip in the FSC@0.143 curve at ~0.1 $Å^{-1}$ (corresponding to ~10 Å). In amyloid fibril reconstructions (typically around 4.7 Å), such features can sometimes arise from imperfect half-map alignment prior to post-processing.

In our case, we believe this dip at ~0.1 $Å^{-1}$ to be an artefact of the noise-substitution test implemented in Relion where phases were randomised past ~10 Å, as it is present in our corrected FSC curves, but not in our masked FSC curves without correction. This is much clearer to readers now that phase-randomised and corrected FSCs are shown overlaid.

The authors further discuss possible binding sites and positional relationships of ABD1a, ABD1b, and ABD2 on F-actin based on the shifted-particle cryo-EM reconstructions approach and propose schematic models illustrated in Fig. 1f and Fig. 3c. While the conceptual approach is compelling, additional quantitative analyses based on _rlnHelicalTubeIDs could further strengthen the conclusions, assuming ABD1a, ABD1b, and ABD2 originate from the same filament-picking job.

13) As a first step, reporting how many particles of each class (ABD1a, ABD1b, ABD2) populate individual _rlnHelicalTubeIDs would be informative. A histogram of particle counts per HelicalTubeIDs could help define a meaningful cutoff (e.g., filaments containing at least seven particles). Using this filtered set, minimal distances between adjacent particles of each class (using the refined particles position for ABD1a, ABD1b, ABD2) along a filament could then be calculated and plotted as distance distributions.

14) In a second step, analyzing class combinations within individual filaments (e.g. filaments containing ABD1a/ABD2, ABD1b/ABD2, or ABD1a/ABD1b/ABD2) could provide additional insight into binding-site preferences and occupancy patterns. Measuring inter-particle distances within these combinations may reveal spatial correlations that further support the proposed schematic models.

This response is regarding comments 13) and 14). We have added an extra figure panel plotting the distribution of 3D classes representing different ABDs/conformations back onto original micrographs, revealing no particular spatial relationship on filaments (Supplementary Figure 16a). We reasoned against further quantitative analysis as 1) a proportion of particles are removed earlier in processing pipeline after particle picking and 2D classification steps (so not all binding sites on a filament are included) and 2) we have been asked by the other reviewers to scale back the paper and make it more concise.

Major Part III: NMR interpretation:

The NMR experiments were conducted using drebrinE 135-355 in the apo state and in the absence of F-actin, as also stated by the authors.
15) Based on the BMRB entries, the N-terminal region of the sequence in BMRB 52895 overlaps with the C-terminal region of BMRB 52729. It would be helpful if the authors could indicate in the 15N-HSQC spectrum which residues originate from which assignment.

We have prepared a panel which indicates which residue assignment transfers originate from each BMRB entry (Supplementary Figure 10b).

16) Typically, CheZOD Z-scores of ≥8 are interpreted as indicative of well-ordered regions, values ≥3 as partially ordered, and values <3 as disordered. In Extended Data Fig. 8, most residues show Z-scores distributed around ~2, while residues in the L3 region cluster around ~0.5. Given these values, it is not clear how the apo-state NMR data support structural agreement with the cryo-EM observations.

We believe our relatively low CheZOD scores are likely due to us only performing

amide nitrogen and proton assignment in this study and using these for our CheZOD profile. The values that the reviewer mention would refer to CheZOD scores obtained using a full backbone assignment. As an independent measure of secondary structure probability, we therefore also calculated RCI-S^2 (order parameter) values from our data using TALOS-N (PMID: 23728592). This is a method that uses database of proteins with known structures and assigned chemical shifts values; an S^2 value below 0.6 is considered as an indication of disorder. The TALOS-N analyses were largely in agreement with both our relative distribution of CheZOD scores and our cryo-EM structures (Supplementary Figure 10c-d).

Minor I:
To date, drebrin has been demonstrated to compete with F-actin depolymerase cofilin, F-actin-bundlers fascin and α-actinin, tropomyosin and myosins, suppressing their activities 55,59-62.
17) Please add the cofilin citation.

Thanks for alerting us to the missing citation. We have added in the citation for PMID: 25047716.

Minor II: Structural competition between drebrinE and fascin, cofilin, α-actinin, tropomyosin and myosins.

18) Most of the work cited by the authors for the competition assay of full-length drebrinE used extracts from embryonic rat brains (some E. coli-expressed) and studied the interaction via spin-down assays. Could the floppy tail of ~346 residues and the ADHF domain also influence binding?

There is a possibility that the ADFH domain and unstructured regions C-terminal to ABD2 (the tail) have an impact on competition. However, we show clearly that drebrin's ABDs are sufficient to explain steric competition with fascin, cofilin, α-actinin, tropomyosin and myosins. We also now more comprehensively demonstrate that there is no density found on F-actin for other regions of drebrin-$E^{1\text{-}431}$ outside of the two ABDs (new Supplementary Figure 4 and Supplementary Figure 1c-d). These points, when considered concomitantly with studies demonstrating no interaction of other drebrin-E regions with F-actin (e.g PMID: 23979715; PMID: 20347847; PMID: 10585290) and our Alphafold3 predictions suggesting the same (Supplementary Figure 9a), would propose the ABDs are chiefly responsible for steric competition with these ABPs.

Reviewer #4 (Remarks to the Author):

Drebrin is one of the key regulators of actin cytoskeleton in neuronal and non-neuronal cells. High resolution structural information on drebrin complexes with the interacting partners could provide valuable new insights on some of its documented cellular activities and biochemical properties. Filamentous actin is recognized as a main drebrin binding factor, thus, this research direction is important and timely. Structural work on F-actin-drebrin complex is complicated by the binding stoichiometry (drebrin binds to multiple actin protomers). This work presents cryoEM reconstruction of two drebrin fragments with actin filaments, combined with the modeling of their relative positions on actin lattice. This resulted in identification of 6 different modes of binding with each class representing 14-33% of total particles.

Based on the structure presented, drebrin-bound F-actin closely resemble ADP-Pi state ("young filaments"), which is interesting and novel. Another interesting observation made is that drebrin binding interface is a better match for actin subunits within a polymer (flattened) as opposed to G-actin. This provides a structural explanation for drebrin's binding preference to actin polymer vs monomers, which is novel and valuable to the field. My main concerns regarding this study include insufficient description of drebrin-actin interface, lack of in vitro validation of the structure, as well as clarity of presentation/writing (detailed below).

We thank the reviewer for recognising the novelty and value of this work.

Major issues (scientific):
The description of the F-actin-drebrin interface is lacking. The authors identify the drebrin residues involved in its interaction with actin but the actin's side of the interface is unclear as well as the chemical nature of the bonds stabilizing it (salt bridges, H-bonds, hydrophobic interactions etc). A complete list of actin and drebrin residues involved in the interaction and the types of bonding should accompany the description of different binding modes. Moreover, key molecular details of the interface (including individual side chains) should be shown in the main figures.

We feel that in the interest of keeping the paper concise and accessible for the broad readership of Nature Communications (a key request of the other reviewers), attempting to show and describe the many hundreds of inter-residue interactions in the three structures is implausible. Readers interested in analysing specific interaction sites in the structure will be able to download the atomic coordinates deposited in the protein database (PDB), from which residue-by-residue interaction analysis software such as PISA can be run.

Nonetheless, we have expanded our presentation of specific interaction sites in our figures. We have now moved panels describing F-actin interfacing residues in drebrin's ABDs and their conservation to main Figure 2. Attempting to further show actin side chains in such images results in overcrowded and uninterpretable figure panels. We have also assessed the contribution of key conserved residues in the ABD1 and ABD2 interfaces with F-actin binding by making additional point/double point mutants of drebrin-$E^{125-256}$ and drebrin-$E^{256-355}$ and analysing their F-actin binding propensity by 1) co-sedimentation (Supplementary Figure 11) or 2) F-actin co-localisation when expressed in COS7 cells (Supplementary Figure 12). In Supplementary Figures 11 and 12 we have included close ups of mutated drebrin residues (in cryo-EM density), now including the side chains of interacting F-actin residues and hydrogen bonds reported by ChimeraX software.

Comparison of the structure with the Alphafold-generated model of the F-actin-drebrin complex (Extended Fig. 1) would be also helpful. The related text needs to be revised.

As requested by another reviewer, we have performed a new Alphafold3 prediction of drebrin-E^{1-431} with F-actin and included the result, alongside original predictions of drebrin-E without F-actin, in Supplementary Figure 9. Panel c now includes comparisons of secondary structure determined with cryo-EM with Alphafold3 predictions in the presence or absence of F-actin. Expanded comparisons of

predicted and experimental structures are now included in the revised text under the new results section 'Comparison of predicted and experimental structures'.

Validation of the structure would be important especially since there are multiple modes of binding. I disagree with the use of filopodia formation in COS7 cells as a readout of drebrin binding (Extended Data Fig. 7d-I). Filopodia formation is a complex process, and drebrin was shown to influence multiple proteins potentially involved in this process (fascin, Arp2/3, formins). Thus, in my opinion, alterations of the filopodia phenotype may not necessarily report on inhibition of drebrin-F-actin interaction. Microscopy or biochemical assays comparing a WT drebrin construct with the site-specific mutants designed to weaken the interface would strengthen the paper. I do find the Extended Data Fig. 7d-I interesting, especially the results with the R221A mutant However, in my opinion, it would be important to compliment this cellular work with the in vitro assays.

The fact that constructs containing either or both ABDs, but not constructs of regions outside the ABDs, lead to filopodial induction (PMID: 23979715) suggests F-actin association is critical to filopodial induction. However, we agree that filopodia formation is complex and may not completely reflect F-actin association propensity of drebrin and its mutants. We have complemented the cellular work and further validated the interface by making double point mutants of drebrin-$E^{125-256}$ and drebrin-$E^{256-355}$ targeting conserved F-actin interfacing residues, then performing F-actin co-sedimentation assays (new Supplementary Figure 11). We also both expanded the number of mutants analysed in the cellular filopodial induction analyses and have additionally performed co-localisation analyses between F-actin (phalloidin) and our eYFP-tagged drebrin mutants (Supplementary Figure 12).

General comments:
Introduction provides an extensive survey of drebrin functions and activities. However, the available low resolution structural information on drebrin-F-actin complex is not mentioned (Ref 2). Authors should revise the Introduction to include the available structural information including the previously reported multiple binding modes.

This study is now briefly included towards the end of the introduction, as well as being compared to our data in the discussion.

I'd like to bring to the authors' attention an issue of the binding stoichiometry which is ~1:5 (full length drebrin : actin) based on data from multiple groups. Citing Ref 55 in context of the 1:3 (drebrin:actin) binding stoichiometry is not factual (the stoichiometry 1:5 was reported in that study for the endogenous drebrin E). This means that a part of F-actin-drebrin interaction interface is still unknown. The authors should discuss how this affects their main conclusions.

We have now included in the discussion section a brief comparison of our data with previous data in regard to binding stoichiometries, including all the literature we could find on the subject. The reviewer is right that generally for full length drebrin purified from rodent brain or expressed in bacteria recombinantly, the reported stoichiometry for actin binding is ~1:5. The 1:3 binding stoichiometry for drebrin to actin reported previously was for a 1-300 drebrin-A construct recombinantly

expressed in bacteria (PMID: 20347847). This construct contains both ABDs, except for the last 3 poorly conserved residues of ABD2 (Figure 2) which are unlikely to contribute significantly to F-actin interaction. The reason for discrepancies in calculated stoichiometries are unclear and may indeed indicate drebrin has additional unpredicted F-actin binding sites outside the ABDs, or additional regions that interfere with ABD-F-actin association, which have not been resolved in this study. However, it is possible some discrepancies could also result from the limitations/challenges in quantitatively interpreting binding stoichiometries from densitometric analyses of Coomassie-stained SDS-PAGE gels.

My understanding is that it is accepted that drebrin is not a strong actin bundling protein. However, it was suggested that drebrin's bundling activity is regulated in the phosphorylation-dependent manner. With the new structural information obtained, the authors should discuss the drebrin phosphorylation pattern in the context of their cryoEM structure. Are any of the phospho-regulated residues directly involved in actin-drebrin interaction? If so, will phosphorylation affect this interaction, potentially allowing only a subset of the possible binding states?

Following low-speed co-sedimentation assays (Figure 4 and Supplementary Figure 17), we have revised our interpretation of our data, concluding that drebrin's tandem ABDs separated by a long flexible loop do have some capacity to loosely bundle F-actin, in a manner which is not visualisable by cryo-EM. We discuss the implications of previously identified phosphorylation sites now being able to be structurally mapped to ABDs or proximal sites in a modified discussion paragraph.

Based on the biochemical data, drebrin has stronger effect on the barbed end of F-actin (compared to the pointed end). The model proposed in Fig. 7 is not consistent with biochemical data which needs to be discussed.

Our structural data only offer clear explanations for drebrin's F-actin stabilising properties, which is most relevant to the pointed end where actin protomers are lost. However, we have now expanded and improved our discussion of how our structures could be mechanistically informative towards previous findings from the Grintsevich group that; 1) drebrin is a permissive F-actin barbed end capper (slowing growth), 2) that the drebrin-A isoform is a stronger capper, due to a potential additional F-actin barbed end binding motif and 3) that drebrin interacts with formins and profilin at barbed ends to inhibit F-actin assembly (PMID: 39971265; PMID: 9473484; PMID: 30625038;PMID: 37898384). We have also included proposed models of drebrin's barbed end activities in Figure 5.

Fig. 3e - % occupancy for all the classes combined does not add up to 100%.

All class occupancies summing to 99% is just due to the rounding (to one significant figure) of component class occupancies, however we felt adding decimals to the class occupancy percentages is unnecessary. We have now rounded all class occupancies to one significant figure and included this information in the figure legends.

Fig. 4j (microtubule entry). Missing control. Although filopodia will be much less common in the cells transfected with eYFP, the authors should present the results of

the data analysis with eYFP alone. The frequency of the microtubule entry is not meaningful without a reference point.

We agree that we would ideally collect tomograms of filopodia in eYFP-transfected cells for comparison. However, filopodia in either untransfected COS7 cells or those transfected with eYFP control have hardly any filopodia (Supplementary Figure 12 and e.g PMID: 23979715). Therefore, when screening in the electron microscope, we found that due to their infrequency, limited transfection efficiency and the small proportion of the EM grid surface that is suitable for tomogram collection (due to ice thickness and distance from grid bars etc) cryo-ET of these targets was unfeasible.

One of my main points of criticism is that the paper (in my opinion) could be written more clearly. Many figure panels appear to be redundant, unnecessary or difficult to understand. Colored letters layered onto the structures obscure the structural features.
This manuscript is very difficult to follow. It needs to be restructured and edited for clarity to be accessible to the general audience. Some of the examples (among many others) are below:
P.3 Unclear. “Picked particles corresponding to decorated segments of F-actin filaments were subjected to image processing including focused classification such as to identify different conformations of drebrin-E135-256 or drebrin-E256-355 if present”
P.9 Unclear. “Furthermore, even within an F-actin protomer pair bound by an ABD, binding of a particular ABP can be sterically permitted only to one of the two protomers.”

We undertook a major rewrite (including the examples included above) and reorganisation of the paper to make it more concise, less deeply descriptive and more easily interpretable to a broad set of readers.

Reviewer 1:
I co-reviewed this manuscript with one of the reviewers who provided the listed reports. This is part of the Nature Communications initiative to facilitate training in peer review and to provide appropriate recognition for Early Career Researchers who co-review manuscripts.

Reviewer 2:
I think the authors' responses and the changes made to the manuscript and figures are appropriate, and I have no further comments or concerns regarding publication of this paper in Nature Communications.

Reviewer 3:
Thank you for sharing the authors' response. I have only two minor points that I would ask the authors to consider before publication.

First, regarding the response to points 8 and 9, I appreciate that the authors tested ModelAngelo model building and compared the single domain datasets with the deposited ABD1 and ABD2 models. However, I would still suggest modifying the figure presentation. In my view, it would be clearer to show all densities in the postprocessed form, since this corresponds to the input format used for ModelAngelo, and then fit the atomic model into the density while displaying the residues explicitly rather than using a cartoon representation. This would make the quality of the density and the model fit easier to assess. I agree that the comparison between the newly built or fitted models and the existing refined structures, ABD1a, ABD1b, and ABD2, is useful.

If I understand correctly, the reviewer here is suggesting showing sharpened densities with a full atomic stick representation for the models and maps shown in the figure we include in the rebuttal. This figure was not intended for inclusion in the manuscript and anyhow we feel showing stick representations and all side chains is inappropriate at the resolutions achieved for the single domain constructs bound to actin.

For the NMR section (16), I remain concerned that the CheZOD analysis may be misleading for readers if the authors did not use the full set of chemical shifts normally required for a meaningful CheZOD calculation. The authors themselves indicate that the relatively low scores likely arise from using only amide nitrogen and proton assignments rather than a full backbone assignment. I would therefore suggest that the CheZOD analysis be removed, and that the authors instead explicitly state that the alpha-helical section of drebrin was already estimated by Varga et al.

In the main text we have now explicitly stated that helix-α2 was already estimated by Varga et al., and have compared this to our experimental data. We have also removed the CheZOD analysis in figures and any mention of it in the text, as suggested.

Overall, these are minor points, but I believe addressing them would improve clarity and avoid potential overinterpretation of the density fitting and NMR analyses.

Reviewer 4:

This manuscript was extensively revised, and the data presentation was significantly improved. A few important points need to be addressed before the publication:
(1) Regarding the validation of the structural work, the biochemical data presented in Supplementary Fig. 11 is convincing. However, a subset of mutants tested in biochemical experiments (283A/286A and 180S/181S) is different from that tested in COS7 cells. Therefore, the statement "We further validated the drebrin-F-actin interface…" (line 199-200) should be removed and replaced with "We tested the functional importance of each ABD1 conformation for filopodial induction…" (line 199-200, P4) to make it factual. The presented cellular work has its own independent value but as the authors recognize in their response, filopodia formation is a complex process and its induction cannot serve as a direct validation of a structure. Thus, only the data shown in Supplemental Figure 11 is connected to such validation.

The suggested change has been made in the text.

(2) It should be clear from the text of the Discussion that this work was done with the truncated drebrin constructs. Thus, the authors should not use a term "drebrin" while describing their data with such constructs (examples include lines 317, 324, 329, 380, 410, and 424). Instead, they should indicate a construct utilized for a specific experiment (such as drebrin135-355 for the bundling data in Fig. 3h (line 380) etc).

As suggested by the reviewer, where appropriate in the discussion we've made clear the drebrin-E constructs used for specific experiments in this study.
